# Abstract

**DOI:** 10.1002/jcsm.12513

**Published:** 2019-12-10

**1-01**

**IL‐1R inhibition reduced tumour growth, inflammation, and fibrosis in cachectic tumour bearing**

**Joanna D.C.C. Lima** ^1^, Estefania Simoes^1^, Stephanie J. Melchor^2^, Marilia Seelaender^1^ and Sarah E. Ewald^2^

^1^ *Department of Cell Biology and Development at the Institute of Biomedical Science, University of Sao Paulo, Sao Paulo, Brazil;* ^2^ *Department of Microbiology, Immunology and Cancer Biology and the Carter Immunology Center, University of Virginia School of Medicine, Charlottesville, VA, USA*

**Background:** Cancer‐associated cachexia (CAC) is a multifactorial disorder characterized by muscle wasting with or without loss of adipose tissue. CAC remains a devastating syndrome affecting 50–80% of cancer patients, and it is responsible for the death of at least 20%. Cachexia is driven by a multiple association of metabolic changes including reduced food intake, enhanced energy expenditure, and inflammation leading to poor prognosis. Inflammatory mediators are derived from tumour cells and host interaction that induces catabolic actions. Commonly, tumour necrosis factor (TNF), IL‐6, transforming growth factor‐β (TGF‐β), IFN‐γ, and IL‐1 are implicated in mechanisms that include activation of inflammation, fibrosis, proteolysis, autophagy, and lipolysis. Interleukin 1 receptor 1 (IL‐1R) recognizes interleukin‐1 (IL‐1)α and IL‐1β, so‐called “alarmins” that are released from dead or damaged cells to initiate inflammatory responses and tissue remodelling and trigger behavioural changes like anorexia. Recently, IL‐1α blockade was shown to induce weight gain and improve quality of like in metastatic cancer patients. The aim of this study was to investigate the role of IL‐1 receptor in the tumour microenvironment of cachectic tumour bearing mice.

**Methods:** C57BL/6 wild type controls or IL‐1R^−/−^ mice (B6.129S7‐il1r tm1/mx/J) were obtained from The Jackson Laboratory. Five million LLCs were injected subcutaneously, and mice were euthanized at 21 days post‐tumour inoculation. *In vivo* bioluminescence of LLC‐GFP cells was measured using the IVIS® imaging system. The mRNA content was evaluated by real‐time PCR (2^−ΔΔCt^) and protein levels by ELISA assay.

**Results:** Weight loss was significantly higher in WT mice relative to IL‐1R^−/−^ mice after accounting for tumour mass (*P* = 0.0056). IL‐1R deficient mice had significantly smaller tumours than WT mice by weight (*P* = 0.015) and by LLC‐GFP signal intensity (*P* = 0.020). mRNA levels of IL‐1βin the tumour was decreased in IL‐1R^−/−^ compared to WT mice (*P* = 0.015). Hence, mRNA and protein levels of TNF‐α (*P* = 0.9914 and *P* =p 0.265, respectively) and IL‐6 (*P* = 0.140 and *P* = 0.389, respectively) were not significantly different between the groups. There were also no differences between groups in IFN‐γ in tumour microenvironment (*P* = 0.337). Furthermore, gene expression of fibronectin 1 was decreased in the tumour of IL‐1R^−/−^ compared to WT mice (*P* = 0.014). Expression of collagen 3, collagen 1, and MMP2 were not significantly altered in tumour of IL‐1R^−/−^ mice comparing with WT.

**Conclusions:** The IL‐1 axis is necessary for LLC growth and cancer‐associated weight loss. The reduction in tumour growth was not due to altered TNF‐α, IL‐6, and IFN‐γ in the tumour environment. The tumour microenvironment showed some evidence of IL‐1R dependent fibrotic tissue remodelling. These experiments suggest that inhibiting the IL‐1 signalling axis may provide a novel target for tumour progression and cancer cachexia treatment.

**1-02**

**Reservatrol inhibits body weight and skeletal muscle mass losses, decreases pro‐myogenic factors, decreases low‐degree systemic chronic inflammation, delays the onset of cachexia, and improves cancer‐related survival in C57BL/6 mice bearing syngeneic tumour**

Otávio Cardoso‐Filho^1^, Magda Mendes Vieira^1^, Amanda Rodrigues da Silva^1^, Valéria Couto Quintão^1^, Lorrane Katherine Martins Pereira^1^, Maria Isabela Alves Bernardo^1^, Vinicius Dias Rodrigues^1,2^, Gefter Thiago Batista Corrêa^3^, Erivelton Pereira dos Santos^1^, Amanda Souto Machado^1^, Ludmilla Regina de Souza David^1,4^ and **Alfredo Mauricio Batista De‐Paula** ^1^

^1^ *Nucleus of Epidemiologic and Molecular Research Catrumano (Nupemoc), Health Research Laboratory, Post‐graduate Programme in Health Sciences, Universidade Estadual de Montes Claros, Unimontes, Montes Claros, Minas Geras, Brazil;* ^2^ *Department of Physical Education, Universidade Estadual de Montes Claros, Unimontes, Montes Claros, Minas Gerais, Brazil;* ^3^ *Department of Dentistry, Faculdades Independentes do Nordeste, Fainor, Vitória a Conquista, Bahia, Brazil;* ^4^ *Department of Dentistry, Universidade Estadual de Montes Claros, Unimontes, Montes Claros, Minas Gerais, Brazil*

**Background:** Cancer‐related cachexia (CRC) is a paraneoplastic syndrome characterized as progressive, systemic physical consumption state of individual during cancer progression. Trans‐resveratrol (3,4,5‐trans‐trihydroxystilbene; Resv) is a naturally occurring polyphenol which modulates inflammatory responses that are usually found in a number of low‐grade, systemic chronic inflammatory diseases. We investigated the effects of Resv administration on inflammatory plasma biomarkers, anthropometrical parameters, skeletal muscle (SM) mass, volume and strength, and survival of C57BL/6 mice bearing a syngeneic cutaneous‐melanoma model (SCMM).

**Methods:** Murine B16F10 cells were injected into flank of the 58 female C57BL/6 mice in order to establish a SCMM. CRC diagnosis was individually established for each animal using as parameter weight loss ≥5%. Resv was administered in concentrations of 200 and 400 mg/kg body weight using oral gavage in both control and experimental mice. Measurements of water and food consumption, body weight, and tumour size were daily performed. High‐sensitive C‐reactive protein (CRP) plasma level was measured by an enzyme immunoassay. SM strength, volume, and mass were assessed using a grip strength meter, a high‐frequency ultrasound, and an analytical balance, respectively. SM samples were collected and posteriorly submitted to morphometric and gene expression (GAPDH, Myog, IGF1, IGF2, FBXO32, TRIM63, and TRIM55) analysis by using real‐time PCR. Control and experimental mice were submitted to cancer‐related survival (CRS) analysis. This study was approved by an ethics committee in animal well‐being and experimentation (CEEBEA/Unimontes No. 131/2017).

**Results:** Mice treated with Resv significantly reduced plasma concentration of CRP, exhibited a delayed in CRC occurrence, showed gain of body weight, and improved CRS rate. Resv administration increased SM tissue mass, volume, and muscle strength. Moreover, mice treated with Resv showed a higher quantity of SM fibres and higher pro‐myogenic factors. The most of these effects promoted by Resv were dose‐dependent.

**Conclusions:** Resv administration promoted a plethora of anticachectic effects on plasma and SM tissues in C57BL/6 mice bearing SCMM, which might influence CRS improvement in CRS.

**1-03**

**Resveratrol negatively modulates pro‐adipogenic gene expression in visceral white adipose tissue of C57BL/6 mice bearing syngeneic cutaneous melanoma**

Ludmilla Regina de Souza David^1^, Otávio Cardoso‐Filho^1^, Andréia de Souza Brito^1^, Bruna Macedo Lima^1^, Gefter Thiago Batista Corrêa^2^, Magda Mendes Vieira^1^, Lorrane Katherine Martins Pereira^1^, Maria Isabela Alves Bernardo^1^, Gabriel Donner Oliveira^1^ and **Alfredo Mauricio Batista De‐Paula** ^1,3^

^1^ *Nucleus of Epidemiologic and Molecular Research Catrumano (Nupemoc), Health Research Laboratory, Post‐graduate Programme in Health Sciences, Universidade Estadual de Montes Claros, Unimontes, Montes Claros, Minas Geras, Brazil;* ^2^ *Department of Dentistry, Faculdades Independentes do Nordeste, Fainor, Vitória da Conquista, Bahia, Brazil;* ^3^ *Department of Dentistry, Universidade Estadual de Montes Claros, Unimontes, Montes Claros, Minas Gerais, Brazil*

**Background:** During tumour progression, might occur cancer‐related cachexia (CRC), a paraneoplastic syndrome characterized as progressive, systemic physical consumption of individual with cancer which modulates inflammatory responses that are usually found in a number of low‐grade, systemic chronic inflammatory diseases. We investigated the effects of Resv administration on inflammatory plasma biomarkers, anthropometrical parameters, white adipose tissue (WAT), and survival of C57BL/6 mice bearing a syngeneic cutaneous‐melanoma model (SCMM).

**Methods:** Murine B16F10 cells were injected into flank of the 58 female C57BL/6 mice in order to establish a SCMM. CRC diagnosis was individually established for each animal using as parameter weight loss ≥5%. Resv was administered in concentrations of 200 and 400 mg/kg body weight using oral gavage in both control and experimental mice. Measurements of water and food consumption, body weight, and tumour size were daily performed. High‐sensitive C‐reactive protein (CRP) plasma level was measured by an enzyme immunoassay. SM strength, volume, and mass were assessed using a grip strength meter, a high‐frequency ultrasound, and an analytical balance, respectively. Visceral WAT samples were collected and submitted to morphometric and pro‐adipogenic gene expression (PPAR‐γ and SREBF1) analysis. Control and experimental mice were submitted to cancer‐related survival (CRS) analysis. This study was approved by an ethics committee in animal well‐being and experimentation (CEEBEA/Unimontes No. 131/2017).

**Results:** Mice treated with Resv significantly reduced plasma concentration of CRP, exhibited a delayed in CRC occurrence, showed gain of body weight, and improved CRS rate. Resv administration reduced WAT relative weight, adipocyte area and number, and PPAR‐γ and SRE expression in WAT. The most of these effects promoted by Resv were dose‐dependent.

**Conclusions:** Although Resv administration negatively modulated WAT in C57BL/6 mice bearing SCMM, positive effects of Resv on CRC and CRS seem to be caused by its systemic anti‐inflammatory effects.

**1-04**

**Tumour‐derived Upd3 cytokine coordinates self‐growth and host wasting**

Guangming Ding^1,2^, Xiaoxiang Xiang^1,2^, Yanhui Hu^3^, Yuchen Chen^1,2^, Richard Binari^3^, Aram Comjean^3^, Elisabeth Rushworth^2^, Zhenming Fu^1^, Stephanie Mohr^3^, Norbert Perrimon^3,4^ and **Wei Roc Song** ^1,2^

^1^ *Department of Oncology, Renmin hospital of Wuhan University, Wuhan, Hubei Province, China;* ^2^ *Medical Research Institute, School of Medicine, Wuhan University, Wuhan, Hubei Province, China;* ^3^ *Department of Genetics, Harvard Medical School, Boston, MA, USA;* ^4^ *Howard Hughes Medical Institute, Boston, MA, USA*

Yki‐induced fly gut tumours are associated with host wasting, including muscle dysfunction and lipid loss, a condition resembling human cancer cachexia. Previously, using this model, we identified two tumour‐derived ligands involved in host wasting. However, how yki‐gut tumours simultaneously coordinate tumour growth and host wasting is still not fully understood. To search for additional pathways involved in interorgan communication, we developed PathON, a software that comprehensively analyzes canonical Drosophila signalling pathways and their matched ligands. Using PathON, we found that the ligand Upd3 and the targets of JAK/STAT signalling are greatly up‐regulated in the tumours and muscle, respectively, of flies bearing yki‐gut tumours. We demonstrate that Upd3 is required for both overproliferation of yki‐gut tumours and host wasting and that Upd3/JAK/STAT signalling impairs energy balance in both muscle and adipose tissues via suppression of insulin response. Altogether, our results demonstrate that yki‐gut tumours produce a single ligand, Upd3, coupling self‐growth and host wasting.

**1-05**

**^18^F‐FDG PET imaging in pre‐clinical tumour‐bearing mouse models of cachexia suggests that Fn14 plays a role in glycolytic changes in the tumour occurring during cancer cachexia**

**Ingrid J. Burvenich** ^1,2^, Laura D. Osellame^3^, Amelia J. Johnston^3^, Angela Rigopoulos^1^, Sylvia J. Gong^4^, Graeme J. O'Keefe^4^, Sze Ting Lee^1,4^, Nicholas J. Hoogenraad^3^ and Andrew M. Scott^4,5,6^

^1^ *Olivia Newton‐John Cancer Research Institute, Melbourne, Australia;* ^2^ *School of Cancer Medicine, La Trobe University, Melbourne, Australia;* ^3^ *Department of Biochemistry and Genetics, La Trobe Institute for Molecular Science, La Trobe University, Melbourne, Australia;* ^4^ *Department of Molecular Imaging and Therapy, Austin Health, Melbourne, Australia;* ^5^ *Department of Medical Oncology, Austin Health, Heidelberg, Melbourne, Australia;* ^6^ *Department of Medicine, University of Melbourne, Melbourne, Australia*

**Introduction:** Cachexia is a syndrome characterized by unintentional weight loss, progressive muscle wasting, and loss of appetite, seen in up to 80% of cancer patients. An antibody (002) that targets the TWEAK receptor (Fn14) has been shown to reverse the symptoms of cachexia in syngeneic tumour‐bearing mouse models and extend the lifespan of mice by restoring their body weight. Here, we investigated via positron emission tomography (PET) imaging the glucose changes in tumour‐bearing mouse models of cachexia, to explore whether Fn14 plays a role in the metabolic changes occurring during cancer cachexia.

**Methods:** ^18^F‐FDG PET was performed in non‐cachectic MEF H‐Ras V12 versus cachectic MEF H‐Ras V12 hFn14 tumour‐bearing Nod SCID gamma mice (NSG) expressing human Fn14. Secondly, ^18^F‐FDG PET imaging was performed in cachectic C26 tumour‐bearing NSG mice treated with anti‐Fn14 002 antibody versus vehicle control treated mice. In the C26 model, 002 therapy was commenced before versus after symptoms of cachexia were measurable.

**Results:** ^18^F‐FDG PET imaging demonstrated increased glucose uptake over time in cachectic versus non‐cachectic tumour‐bearing mice. This was observed both in the MEF H‐Ras V12 hFn14 model as well as in the C26 model. Targeting Fn14 with 002 was able to prevent increased ^18^F‐FDG uptake in C26 tumours, but more importantly, tumours of cachectic C26 mice with high ^18^F‐FDG uptake showed reduced ^18^F‐FDG after 2 days of therapy with 002.

**Conclusions:** Our results demonstrate that cachexia associated with Fn14 signalling is associated with increased tumour glucose metabolism and that ^18^F‐FDG PET imaging could be used to monitor patient response to mAb 002 cachexia treatments in clinical trials.

**1-06**

**Feeding regulation of skeletal muscle 4E‐BP1 is disrupted in female tumour bearing mice initiating cachexia**

**Brittany R. Counts** and James A. Carson

*Integrative Muscle Biology Laboratory, Division of Rehabilitation Sciences, College of Health Professions, University of Tennessee Health Science Center, Memphis, TN, USA*

**Background:** The mechanistic target of rapamycin complex 1 (mTORC1) integrates skeletal muscle's response to nutrients involving protein translation, ribosome biosynthesis, autophagy, and oxidative metabolism. The phosphorylation of S6K1, rpS6, and 4E‐BP1 by mTORC1 regulates protein translation and ribosome biosynthesis. Furthermore, these proteins have differential sensitivity to mTORC1 activation, and 4E‐BP1 phosphorylation can regulate muscle oxidative metabolism. While cancer can suppress basal muscle mTORC1, gaps remain in our understanding of how the feeding regulation of mTORC1 is impacted by cancer cachexia. We examined the effect of feeding and fasting on skeletal muscle mTORC1 signalling in female *Apc* ^*Min/+*^ (MIN) mice.

**Methods:** Female C57BL/6 (B6) and MIN mice were fasted for 12 h during the light cycle. Following the 12 h fast, mice were given access to a food pellet for 1 h (B6 FED:N = 7, MIN FED:N = 8) or fasted for another hour (B6 FAST:N = 6, MIN FAST:N = 7). Blood glucose, plasma IL‐6, and insulin were measured. Gastrocnemius muscle was homogenized for protein analysis.

**Results:** MIN mice were initiating cachexia at the end of the study. The MIN FED mice were not different from the MIN FAST for per cent body weight change, polyp number, plasma IL‐6, and hindlimb muscle mass. Feeding increased stomach mass, blood glucose, and circulating insulin levels in FED B6 and MIN mice. Compared to FAST mice, FED mice increased S6K1 and rpS6 phosphorylation and was positively associated to insulin levels in both B6 and MIN mice. While feeding increased 4E‐BP1 phosphorylation in B6 mice, this response was disrupted in the MIN. Additionally, insulin was associated to 4E‐BP1 phosphorylation in the B6, but not in the MIN.

**Conclusions:** The feeding regulation of muscle 4E‐BP1 is disrupted by the cancer environment during the initiation of weight loss, and further investigation is warranted to determine if this is an earlier driver of cancer‐induced skeletal muscle metabolic dysfunction.

**Acknowledgement:** NCI R01‐CA121249

**1-07**

**Skeletal muscle and liver gene reprogramming during cancer cachexia in *Apc*^*Min/+*^ mice: potential role of glucocorticoids**

**Agnès Martin** ^1^, Josiane Castells^1^, François B. Favier^2^, Cindy Zolotoff^1^, Valentine Allibert^1^, Yann S. Gallot^3^, Anne‐Cécile Durieux^1^, Christophe Hourde^4^ and Damien G. Freyssenet^1^

^1^ *Laboratoire Interuniversitaire de Biologie de la Motricité, Univ. Lyon, Université Jean Monnet Saint Etienne, Saint Etienne, France;* ^2^ *Dynamique Musculaire et Métabolisme, Univ. Montpellier, INRA, Montpellier, France;* ^3^ *Laboratoire de Biologie de l'Exercice pour la Performance et la Santé, Univ. Evry, Université Paris‐Saclay, Evry, France;* ^4^ *Laboratoire Interuniversitaire de Biologie de la Motricité, Université Savoie Mont Blanc, Chambéry, France*

**Introduction:** Cancer cachexia is characterized by a marked loss of skeletal muscle mass that strongly contributes to reduce patients' quality of life and ultimately patients' lifespan. Skeletal muscle is a metabolic partner to the liver, and perturbations in muscle‐liver axis can contribute to cancer cachexia. As glucocorticoids exert metabolic action on both tissues, the purpose of this study was to investigate the potential role of glucocorticoids on skeletal muscle and hepatic metabolisms during cancer cachexia.

**Methods:** *Apc* ^*Min/+*^ mice were used as cancer cachexia model. Quadriceps muscle, liver, and blood samples were removed from 13‐ (beginning of cachexia) and 23‐ (advanced cachexia) week‐old *Apc* ^*Min/+*^ mice and C57Bl6/J wild‐type littermates.

**Results:** *Apc* ^*Min/+*^ mice recapitulated main features of cancer cachexia, i.e. body mass loss, adipose tissue, and skeletal muscle mass loss and decreased muscle force. Cancer cachexia was associated with an imbalance in skeletal muscle proteostasis toward a reduction in proteosynthesis and an increase in proteolysis. In liver, cancer cachexia was associated with a complete gene reprogramming characterized by an increased expression of genes involved in neoglucogenesis and a decreased expression of genes involved in ketogenesis and lipid synthesis. Corticosterone concentration was significantly increased in the serum, quadriceps muscle, and liver of 23‐week‐old *Apc* ^*Min/+*^ versus wild type mice. The transcriptional signature in quadriceps muscle and liver of 23‐week‐old *Apc* ^*Min/+*^ mice was almost completely reproduced in mice treated with dexamethasone, a glucocorticoid analog. Preventing skeletal muscle mass loss by Myostatin gene invalidation in *Apc* ^*Min/+*^ mice restored corticosterone levels and abolished skeletal muscle and hepatic gene reprogramming.

**Conclusions:** Our data strongly suggest that glucocorticoids act systematically during cancer cachexia to drive a transcriptional programme that co‐ordinately regulate skeletal muscle wasting and hepatic metabolic reprogramming.

**1-09**

**Astaxanthin contribute to ameliorating insulin resistance and muscle remodelling**

**Allah Nawaz** ^1,2^, Yasuhiro Nishida^1^, Tomonobu Kado^1^, Muhammad Bilal^1^, Shiho Fujisaka^1^, Yagi Kunimasa^1^, Takashi Nakagawa^2^ and Kazuyuki Tobe^1^

^1^ *First Department of Internal Medicine, University of Toyama, Sugitani, Toyama‐shi, Toyama, Japan;* ^2^ *Department of Metabolism and Nutrition, University of Toyama, Sugitani, Toyama‐shi, Toyama, Japan*

**Introduction:** Astaxanthin (AX), a natural antioxidant, has been shown to ameliorate insulin resistance in animals. Although much is known about the effects of AX on the liver under the high‐fat‐diet (HFD) condition, the effects of AX in the skeletal muscle of mice reared on a HFD mice are still poorly understood.

**Methods:** We used 6‐week‐old male C57BL/6J mice fed with normal chow (NC) or NC supplemented with AX (NC + AX) and HFD or HFD supplemented with AX (24 weeks). We assessed effect of AX on various parameters including insulin sensitivity, glucose uptake, inflammation, kinase signalling, gene expression, and mitochondrial function in muscle. Analysis of energy metabolism in intact C2C12 cells treated with AX was performed using the Seahorse XF96 Extracellular Flux Analyzer. Oxidative stress was evaluated by measuring the production of malondialdehyde and thiobarbituric acid‐reactive substances.

**Results:** Herein, we showed that AX activated AMPK in the muscle and up‐regulated the expressions of a transcriptional coactivator and transcriptional factors, thereby inducing mitochondrial remodelling, including increased mitochondrial oxidative phosphorylation and FFA metabolism, independently of its antioxidant activities. A hyperinsulinaemic–euglycaemic clamp study also revealed that AX improved glucose metabolism by improving glucose incorporation into peripheral target tissues, such as the skeletal muscle, rather than by suppressing gluconeogenesis in the liver of the HFD mice; this was considered to be attributable to activation of the AMPK pathway in the skeletal muscle, leading to enhanced mitochondrial activity and FFA utilization in the HFD mice. In fact, flux analyzer analysis revealed that AX treatment enhanced the mitochondrial oxy‐phosphorylation capacity and FFA utilization in C2C12 myotubes. Both *in vivo* and *in vitro* studies revealed that AX administration enhanced AMPK activation in muscle.

**Conclusions:** We concluded that AX treatment stimulated mitochondrial biogenesis and significantly ameliorated insulin resistance through activation of AMPK pathway in the skeletal muscle.

**1-10**

**Leucine nutritional supplementation improved the basal metabolic rate and muscle metabolomic profile in an experimental model of cachexia**

Rogerio Willian dos Santos, Bread Cruz, Lais Rosa Viana and **Maria Cristina Cintra Gomes‐Marcondes**

*Laboratory of Nutrition and Cancer, Department of Structural and Functional Biology, Biology Institute, University of Campinas (UNICAMP), Brazil*

**Introduction:** Cancer‐cachexia is an important clinical problem, reducing life expectancy, as tumour evolution leads to skeletal muscle mass wasting, mediated by the proteolysis and/or reduced protein synthesis. Leucine can maintain lean body mass by stimulating muscle protein synthesis and inhibiting proteolysis. We evaluated the basal metabolic rate and the skeletal muscle metabolomic profile under leucine and/or tumour effects.

**Methods:** Wistar rats were distributed in four groups: Control (C, *n* = 5), Walker tumour‐bearing (W, *n* = 7), leucine‐rich diet (L, *n* = 5) and tumour‐bearing subjected to leucine‐rich diet (LW, *n* = 9), and morphometric and basal metabolic rate were measured on 21st day of tumour evolution. After euthanasia, the metabolomic profile in gastrocnemius muscles was accessed by ^1^H‐NMR spectroscopy, following the bioinformatics tool using http://www.metaboanalyst.ca.

**Results:** We observed that W group had a deep decrease in carcass‐to‐initial body weight ratio (29% lower; *P* = 0.0003), intense reduction in muscle mass (27%; *P* < 0.0001), higher cachexia index, and also reduced basal metabolic rate (25%; *P* = 0.0575) when compared to C group. The leucine‐rich diet (LW group) minimized these parameters being different compared to W group. The analyses showed around 44 metabolites in gastrocnemius muscles, and 12 enrolled to energy metabolism when compared both tumour‐bearing groups with the controls, independently of nutritional supplementation. However, the leucine supplementation led to changes in tumour‐induced muscle damage, since the LW group had an increase in muscle G6P and pyruvate, whereas reduced in W group, indicating mainly glycolytic activity, as the most impacted pathways were glycolysis and gluconeogenesis and branched‐chain amino acids metabolism. Even though the muscle 3‐methylhistidine, the main biomarker of skeletal muscle wasting, enhanced in both tumour‐bearing groups, independent of nutritional scheme.

**Conclusions:** Our study showed beneficial effects of leucine supplementation (metabolomic analyses and basal metabolic rate), improving the muscle glycolytic metabolic pathways under tumour‐induced damages.

**Acknowledgements:** Supported by FAPESP; CNPq; FAEPEX.

**1-11**

**Effects of low and high doses of fenofibrate on protein, amino acid, and energy metabolism in rat muscle**

**Milan Holeček** and Melita Vodeničarovová

*Department of Physiology, Charles University, Faculty of Medicine in Hradec Kralove, Czech Republic*

**Introduction:** A feared adverse effect of dyslipidaemia therapy by fibrates is myopathy, pathogenesis of which is unknown. The focus of our study was the effect of fenofibrate (FF) on protein and amino acid metabolism in muscles.

**Methods:** Rats received daily by oral gavage with a low (50 mg/kg, LFFD) or high (300 mg/kg, HFFD) dose of FF or vehicle for 10 days. At the end of the study, the animals were euthanized, and samples of blood plasma, liver, and muscles of different content of red and white fibres were collected for analysis.

**Results:** The FF‐treated rats developed hepatomegaly associated with greatly increased hepatic carnitine content (23‐fold after LFFD; 27‐fold after HFFD), decreased protein breakdown in proteasomes, and decreased concentrations of DNA and triglycerides. HFFD increased plasma alanine aminotransferase and aspartate aminotransferase activities. The weight and protein content of all muscles in the HFFD group was lower compared with controls. In LFFD group, decreased mass was found only in the extensor digitorum longus. FF decreased carnitine and altered concentrations of citric cycle intermediates and adenine nucleotides in muscles. In the LFFD group, increased fumarate and adenosine triphosphate (ATP) and decreased adenosine monophosphate (AMP); in the HFFD group, decreased citrate and increased AMP‐to‐ATP ratio. In both groups of FF‐treated animals, increased glycine and decreased arginine in blood plasma and muscles. After HFFD, decreased also branched‐chain amino acids (BCAA; valine, leucine, and isoleucine), methionine, and lysine in blood and muscles with a high content of white fibres; in blood plasma, homocysteine was increased and branched‐chain keto acids (BCKA) were decreased.

**Conclusions:** We conclude that FF exerts protein‐anabolic effects on the liver and catabolic ones on muscles. HFFD causes signs of hepatotoxicity, impairs energy and protein balance in muscles, and decreases BCAA, methionine, and lysine.

**Acknowledgement:** Supported by PROGRES Q40/02 program.

**1-12**

**β‐Hydroxybutyrate replacement therapy does not protect against cachexia in mice with lung adenocarcinoma**

Seo‐Kyoung Hwang, Rahul Grover, Roger Liang, Shakti Ramsamooj, Lewis C. Cantley and **Marcus D. Goncalves**

*Weill Cornell, New York, USA*

**Introduction:** The cancer‐associated cachexia syndrome (CACS) is a systemic metabolic disorder characterized by wasting of body tissues that store nutrients. CACS is particularly prevalent in patients with advanced lung adenocarcinoma where over 80% of people are affected. We have shown that the *Kras* ^*G12D/+*^ *;Lkb1* ^*flox/flox*^ (KL) mouse model accurately reproduces the clinical features of lung cancer‐induced CACS. In this model, ketogenesis is disrupted in the liver of cachexic mice due to a lack of PPAR‐α activity. Treatment with fenofibrate, a PPAR‐α agonist, restores ketogenesis and prevents the onset of CACS. We hypothesized that the loss of β‐hydroxybutyrate (BHB), the major blood ketone, is necessary for the development of CACS in KL mice, and designed dietary strategies to replace BHB.

**Methods:** In two randomized, controlled, dietary intervention studies, cohorts (*N* = 21–24) of KL mice were randomized to a normal chow (NC) diet or one of two diets: a ketogenic diet (KD) or a NC diet infused with a 1,3‐butanediol acetoacetate diester (KE).

**Results:** The KD effectively increased serum BHB as compared to a NC diet (1.7 ± 0.3 vs. 0.1 ± 0.0, *P* = 0.0001). The incidence and timing of CACS were similar in both arms of the KD study (6/8 mice on KD and 4/7 mice on NC). There was a trend for the KD to worsen overall survival (7.6w vs. 9.6w, *P* = 0.07), increase lung mass (420.1 ± 103.7 vs. 669.6 ± 109.5, *P* = 0.12), and reduce gastrocnemius mass (111.5 ± 3.6 mg vs. 122.5 ± 4.4, *P* = 0.0649). The KE diet had no effect on any of these parameters.

**Conclusions:** These findings suggest that BHB does not protect against CACS and may actually worsen survival in this model. We conclude that BHB is only a biomarker of a more complex change in hepatic and systemic metabolism and does not play a pathophysiologic role in lung adenocarcinoma‐induced CACS.

**1-13**

**Blockade of ACVR2B preserves skeletal and cardiac muscle function in the presence of metastatic colorectal cancer**

**Joshua R. Huot** ^1,2^, Leah J. Novinger^3^, Fabrizio Pin^2,4^, Ashok Narasimhan^1^, Alyson L. Essex^4^, Teresa A. Zimmers^1,2,3,6,7^, Monte S. Willis^4,5,6,7^ and Andrea Bonetto^1,2,3,5,6,7^

^1^ *Department of Surgery;* ^2^ *IUPUI Center for Cachexia Research, Innovation and Therapy;* ^3^ *Department of Otolaryngology—Head & Neck Surgery;* ^4^ *Department of Anatomy, Cell Biology and Physiology;* ^5^ *Department of Pathology;* ^6^ *Indiana Center for Musculoskeletal Heath;* ^7^ *Simon Cancer Center, Indiana University School of Medicine, Indianapolis, IN, USA*

**Introduction:** Advanced colorectal cancer (CRC), a leading cause of death worldwide, is often accompanied by the development of liver metastases (LM), as well as skeletal muscle (SKM) wasting, i.e. cachexia. Activin receptor type 2B (ACVR2B)‐mediated signalling participates in causing SKM wasting in several disease conditions, and its inhibition restores SKM mass and prolongs survival in cancer cachexia. Despite plaguing a majority of CRC patients, cachexia remains understudied and uncured. Moreover, only a single model of LM associated with CRC has been developed for the study of cachexia. We aimed to generate and characterize a new model of CRC and investigate whether systemic blockade of ACVR2B signalling could preserve skeletal and cardiac muscle function in the presence of metastatic cancer.

**Methods:** NSG male mice (8 weeks old) were injected intrasplenically with HCT116 human CRC cells (mHCT116), while sham‐operated animals received saline (*n* = 5–10 per group). Sham and tumour‐bearing mice received weekly injections of ACVR2B/Fc, an inhibitor of ACVR2B, and were monitored weekly for skeletal muscle function and body composition. Conscious ultrasound echocardiography was performed the day before euthanasia.

**Results:** mHCT116 hosts presented significant losses in body weight (−7%), SKM mass (quadriceps: −23%) and strength (−21%), along with impaired cardiac function (EF%: −14%, FS%: −14%). Conversely, administration of ACVR2B/Fc completely preserved SKM mass (quadriceps: +31%) and strength (+29%) in mHCT116 hosts. Interestingly, cardiac function was also completely restored in mHCT116 hosts receiving ACVR2B/Fc (EF%: +14%; FS%: +13%).

**Conclusions:** Our model recapitulates the cachectic phenotype of metastatic CRC by displaying reduced SKM mass and strength in mHCT116 hosts. Additionally, we showed that HCT116 LM severely affects cardiac function, supporting the development of cardiac cachexia. ACVR2B antagonism fully preserved SKM mass, strength, and cardiac function, further dictating that activin signalling represents a promising therapeutic target for preservation of skeletal and cardiac muscle size and function in cancer cachexia.

**1-14**

**APLN and OSMR are altered due to elevated cytokines and cancer cachexia in the heart**

Max C. McGill^1,2^, Angie M.‐Y. Shum^1,2^, Theo Mahendradatta^1,2^, Nicholas L. Bentley^1,2^, Enoch Chan^1,2^, Zhiliang Wu^3^, Marc Wilkins^3^, Timothy C. Tan^1,2,4^ and **Patsie Polly** ^1,2^

^1^ *Mechanisms of Disease and Translational Research;* ^2^ *Department of Pathology, School of Medical Sciences;* ^3^ *Systems Biology Initiative, Ramaciotti Centre for Genomics, School of Biotechnology and Biomolecular Sciences; UNSW, Sydney, NSW, Australia;* ^4^ *Department of Cardiology, Blacktown Hospital, NSW, Australia*

**Introduction:** Cancer cachexia is a chronic inflammatory syndrome defined by greater than 5% body mass loss with underlying malignancy. It affects more than 50% of advanced cancer patients, contributing to approximately 20% of cancer patient deaths, primarily due to cardiac wasting and failure. The heart undergoes maladaptive remodelling in cancer cachexia, leading to altered function. Molecular mechanisms underlying this cardiopathology are relatively unknown. The aim of this project was to determine potential candidate genes altered in response to elevated cytokines in cardiomyocytes.

**Methods:** Analysis of RNA‐sequencing data from cytokine treated HL‐1 cardiomyocytes, to emulate cancer cachexia, was performed to identify potential candidate genes altered in response to elevated cytokines. Analysis for differential gene expression was performed using EdgeR and DeSeq2 statistical software and applying two fields, an FDR < 0.1, and a log_2_ fold‐change deviation of ±0.2 from untreated control levels. Differentially expressed genes that were identified were then validated in cytokine‐treated HL‐1 cardiomyocytes and colon 26 (C26) carcinoma mouse hearts using qRT‐PCR, western blotting, and immunohistochemistry.

**Results:** We have identified alterations in gene expression of key signalling pathways involving Apelin receptor (APLNR) and Oncostatin M receptor (OSMR) in C26 hearts, raising the hypothesis that altered expression of these key signalling molecules may modulate this observed cardiopathology. RNA sequencing results revealed a down‐regulation in APLNR gene and protein expression and an increase in OSMR gene and protein expression in both the cytokine‐treated HL‐1 cardiomyocytes. qRT‐PCR, western blotting experiments, and immunohistochemistry showed consistent results in the hearts of C26 mice with cachexia compared to non‐tumour bearing controls.

**Conclusions:** The down‐regulation of Aplnr, which is implicated in signalling cascades modulating cardiac contractility, and Osmr, which plays a role in signalling cascades involved in the regulation of sarcomeric protein expression, provide new insights into the pathophysiology of cancer induced cardiac cachexia seen in C26 hearts.

**1-15**

**Cardiac cachexia is an independent predictor of survival in dogs with heart failure**

Deanna L. Ineson, **Lisa M. Freeman** and John E. Rush

*Cummings School of Veterinary Medicine, Tufts University, North Grafton, MA, USA*

**Background:** Heart disease affects 10–15% of all pet dogs and can result in heart failure, which is often associated with cardiac cachexia (defined as loss of muscle). In humans, cardiac cachexia is associated with shorter survival, but this has not been reported in dogs. Therefore, the aim of this study was to evaluate the effect of cardiac cachexia on survival in dogs with heart failure.

**Methods:** Dogs with heart failure (Stage C or D) due to naturally occurring myxomatous mitral valve disease (MMVD) or dilated cardiomyopathy (DCM) and evaluated between 2015 and 2018 were eligible. Medical records data recorded included body weight, body condition score (BCS), and muscle condition score (MCS). Cachexia was defined as any muscle loss using the validated World Small Animal Veterinary Association scoring system of normal muscle condition or mild, moderate, or severe muscle loss.

**Result**s: Median age of the dogs (*n* = 269) was 11.0 years (range, 1.8–17.1 years); 54% were male. Mean body weight was 7.9 kg (range, 2.1–82.0 kg). Only 12 dogs were underweight (4.5%), 157 were ideal weight (58.6%), and 99 were overweight (36.9%). Cachexia was present in 48.3% of dogs: mild muscle loss: 101/269 (37.6%), moderate muscle loss: 22/269 (8.2%), and severe muscle loss: 7/269 (2.6%). Dogs with cachexia had a median survival time from onset of heart failure of 233 days (range, 0–1200 days) compared to dogs without cachexia (321 days, range 1–1264 days; *P* = 0.036). On multivariable analysis, cachexia, presence of arrhythmia or azotaemia, and being under‐ or overweight were independent risk factors for shorter survival time.

**Conclusions:** Cardiac cachexia was present in nearly half of all dogs with heart failure and was associated with a shorter survival time.

**2-01**

**Autocrine activin A signalling regulates secretion of interleukin 6, autophagy, and cachexia**

**Kristine Pettersen** ^†, 1,2^, Sonja Andersen^1^, Anna van der Veen^2^, Unni Nonstad^2^, Shinji Hatakeyama^6^, Christian Lambert^6^, Estelle Lach‐Trifilieff^6^, Siver Moestue^3^, Jana Kim^3^, Bjørn Henning Grønberg^4,5^, Alain Schilb^6^, Carsten Jacobi^6^ and Geir Bjørkøy^1,2^

^1^ *Department of Biomedical Laboratory Science, Faculty of Natural Sciences, NTNU‐Norwegian University of Science and Technology, Trondheim, Norway;* ^2^ *Centre of Molecular Inflammation Research, Department of Clinical and Molecular Medicine, NTNU‐Norwegian University of Science and Technology, Trondheim, Norway;* ^3^ *Department of Circulation and Medical Imaging, Faculty of Medicine, NTNU‐Norwegian University of Science and Technology, Trondheim, Norway;* ^4^ *Department of Cancer Research and Molecular Medicine, NTNU‐Norwegian University of Science and Technology, Trondheim, Norway;* ^5^ *Clinic of Oncology, St. Olavs Hospital, Trondheim University Hospital, Trondheim, Norway;* ^6^ *Novartis Institutes for BioMedical Research Basel, Musculoskeletal Disease Area, Novartis Pharma AG, Basel, Switzerland*

^†^These authors contributed equally.

Our understanding of the underlying mechanisms that cause cancer cachexia is limited. Several factors and inflammatory mediators released from the tumour have been suggested to contribute to weight loss in cachectic patients. However, inconsistencies between studies are recurrent. Activin A and interleukin 6 (IL‐6) are among the best studied factors that seem to be important. Several studies support their individual role in cachexia development. We show that activin A acts in an autocrine manner to promote the synthesis and secretion of IL‐6 from cancer cells. By inhibiting activin A signalling, using biological, chemical, or genetic approaches, the production of IL‐6 from the cancer cells is reduced by 40–50%. Inhibiting activin signalling also reduces the ability of the cancer cells to accelerate autophagy in non‐cancerous cells (up to 43% reduced autophagy flux). In line with the *in vitro* data, the use of an anti‐activin receptor 2 antibody in cachectic tumour‐bearing mice reduces serum levels of cancer cell‐derived IL‐6 by 62%, and importantly, it reverses cachexia and counteracts loss of all measured muscle groups. Our data support a functional link between activin A and IL‐6 and indicate that interference with activin A‐induced IL‐6 secretion from the tumour has therapeutic potential for cancer‐induced cachexia.

**2-02**

**The mechanical stimulation of myotubes counteracts the effects of tumour‐derived factors through IL‐4 secretion and the modulation of the activin/follistatin ratio**

**Dario Coletti** ^1,2,3^, Alexandra Baccam^1,2,3^, Alexandra Benoni^1,2,3^, Marco Rocch^4^, Viviana Moresi^1,2^, Marilia Seelaender^5^, Zhenlin Li^2^, Caterina Gargano^1^, Sergio Adamo^1,3^ and Zhigang Xue^2^

^1^ *Biology of Adaptation and Aging (B2A), Sorbonne Université, UMR8256—INSERM ERL U1164, Paris, France;* ^2^ *Section of Histology, Department of Anatomical, Histological, Forensic and Orthopedic Sciences, Sapienza University of Rome, Rome, Italy;* ^3^ *Interuniversity Institute of Myology, Rome, Italy;* ^4^ *Department of Biomolecular Sciences, University of Urbino, Urbino, Italy;* ^5^ *Institute of Biomedical Sciences, Faculdade de Medicina, University of São Paulo, São Paulo, Brazil*

Exercise counteracts cachexia, but it is unclear to which extent the exercise‐dependent mechanical stimulation of muscle *per se* plays a role in exercise beneficial effects. To study the mechanisms underlying mechanical stimulation, we cultured C2C12 myotubes in the absence or in the presence of a cyclic mechanical stretching stimulus (MS) and in the absence or presence of C26 tumour‐derived factors (C26‐CM), so as to mimic the mechanical stimulation of exercise and cancer cachexia, respectively. We found that C26‐CM contains activin and induces activin release by myotubes, further exacerbating its negative effects, consisting in myotube atrophy and in hampering myoblast recruitment and fusion into myotubes. A high level of circulating activin is an adverse prognostic factor in cancer patients, and our *in vitro* results demonstrate that activin may be a direct player and not just a marker of cachexia. We also found that MS is sufficient to counteract the adverse tumour‐mediated effects on muscle cells, in association with an increased follistatin/activin ratio in the cell culture medium, indicating that myotubes actively release follistatin upon stretching. In addition, MS induces IL‐4 secretion by muscle cells. Recombinant follistatin counteracts C26 tumour effects on myotubes exclusively by rescuing fusion index, while recombinant IL‐4 ameliorates fusion index, as well as the myotube size, both in terms of myotube diameter and number of nuclei per myotube. Our results indicate that tumour cells negatively affect muscle cells by releasing soluble factors and that MS is sufficient to counteract these effects, by affecting the muscle secretome with autocrine/paracrine pathways. Activin and Act‐R ligands are becoming increasingly important as triggers of muscle wasting and as pharmacological targets to treat cachexia; however, since follistatin alone is incapable to entirely block the C26‐CM effects, the development of novel activin‐targeted approaches should consider the existence of further significant tumour‐secreted factors mediating cachexia.

**2-03**

**Generation of reporter cell lines to identify and characterize cachexia‐inducing factors**

**Zhipeng Cao**, Laura D. Osellame, Irvin Jose, Hamsa Puthalakath and Nick J. Hoogenraad

*Department of Biochemistry and Genetics, La Trobe Institute for Molecular Science, La Trobe University, Melbourne, Australia*

**Introduction:** Cancer cachexia is mainly characterized by weight loss attributed to loss of muscle and adipose tissue, which can be induced by multiple factors, either derived from the tumour or the host. Many of these factors have been identified; however, more still remain to be discovered. In cancer cachexia, some genes are constitutively activated in target tissues, such as MuRF1 (muscle RING finger‐containing protein 1) and atrogin‐1 in muscle, CIDEA (cell death‐inducing DFFA‐like effector A) in adipose tissue. Utilizing promoters from MuRF1, Atrogin‐1, and CIDEA, we have generated reporter cell lines that are capable of detecting cachexia‐inducing factors released by tumour.

**Methods:** The promoters of genes encoding MuRF1, Atrogin‐1, and CIDEA were cloned into a vector which drives the reporter genes, luciferase and GFP. Constructs were stably integrated into C2C12 myoblasts and 3T3‐L1 pre‐adipocytes, which when differentiated into myotubes and white adipose adipocytes, express receptors for soluble cachexia‐inducing factors. Cells were differentiated and tested using various cachexia‐inducing factors.

**Results:** Dexamethasone, activin A, and myostatin can activate C2C12 myotubes reporter cells. Thiazolidinediones can activated 3T3‐L1 adipocytes. Similarly, serum from cachectic mice was also able to activate these reporter cells, compared to serum from non‐cachectic mice which could not.

**Conclusions:** These reporter cell lines can react to the stimulation of cachexia‐inducing factors. Additionally, they can also be used as a potential diagnostic tool by detecting cachectic factors in the serum or plasma of animals and patients.

**2-04**

**Cachexia induced by non‐bone metastatic cancers is accompanied by bone, cartilage, and bone marrow destruction**

**Fabrizio Pin** ^1^, Lynda F. Bonewald^1,3^ and Andrea Bonetto^1,2,3,4^

^1^ *Department of Anatomy, Cell Biology and Physiology;* ^2^ *Department of Surgery;* ^3^ *Indiana Center for Musculoskeletal Health;* ^4^ *Simon Cancer Center, Indiana University School of Medicine, Indianapolis, IN, USA*

**Background:** Cancer‐induced cachexia resulting in defects in skeletal muscle mass and function is well described, although little is known about the effects of non‐bone metastatic cancer on bone, particularly in the absence of metastases. In this study, we investigated the effects of two well‐characterized models of cancer cachexia, namely, the mice bearing C26 adenocarcinoma and ES‐2 ovarian cancer.

**Methods and Results:** Even though both C26 and ES‐2 tumours resulted in comparable body and muscle wasting, the ES‐2 hosts showed severe bone loss by microCT analysis, whereas only a modest bone loss was observed in the C26‐bearing mice. Histomorphometry analysis showed increased osteoclast numbers in femoral trabecular bone from ES‐2 hosts, while no significant effects were observed in the C26‐bearing mice at time of sacrifice. Von Kossa staining of femur sections showed severe reduction of organized osteoblasts as well as osteoid of trabecular bone. Interestingly, both models showed dramatic increase in osteocyte death and empty lacunae, likely due to secreted tumour factors. This was also validated by *in vitro* experiments showing increased death of MLO‐Y4 osteocyte‐like cells when exposed to ES‐2 or C26 conditioned media. Notably, dramatic depletion of fat vacuoles within the bone marrow, as well as dystrophic mineralization, likely consequence of massive bone marrow cell death, were observed in both models. In addition, the growth plate was also severely affected showing absence of hypertrophic chondrocytes and cartilage degeneration zone in both tumour models.

**Conclusions:** Here, we showed for the first time that bone destruction accompany muscle depletion in two models of non‐metastatic cancer cachexia. Overall, this study suggests the importance of monitoring bone quality in cancer patients, even in the absence of bone metastases. Moreover, our ongoing studies will define whether anti‐resorptive treatments will preserve bone mass while also improving muscle size and function in cachexia.

**2-05**

**Exploring the contribution of the liver to cancer cachexia development**

**Claudia‐Eveline Molocea**, Søren Fisker Schmidt, Natalie Krahmer, Stephan Herzig and Mauricio Berriel Diaz

*Helmholtz Center Munich, Institute for Diabetes and Cancer (IDC), Neuherberg, Germany*

Traditionally, skeletal muscle and adipose tissue have been the focus of research on cancer cachexia. Recent studies suggest that cancer cachexia (CC) is a multi‐organ syndrome which involves not only the direct effects of tumour‐derived cachexia‐inducing factors on target organs but also the crosstalk of these dysfunctional organs. Despite its central role in other metabolic diseases, the liver has received little attention in the context of CC. However, CC is associated with systemic inflammation, and the activation of the hepatic acute phase response (APR) is well‐documented in cachectic patients. Serum amyloid A 1 and 2 (SAA 1/2) are classical APR proteins that are mainly produced by the liver and strongly induced in response to inflammatory cytokines. To assess the potential contribution of SAA to tissue wasting *in vitro*, we treated 3T3‐L1 adipocytes and C2C12 myotubes with recombinant SAA1. While lipolysis rates remained unaffected, we found a significant reduction in myotube diameter in response to SAA1 treatment, indicative of an atrophic effect. Next, we addressed the contribution of SAA 1/2 to cachexia development *in vivo* using a liver‐specific knock‐down (KD) approach. For this purpose, we injected an AAV‐miRNA targeting SAA1/2 or an AAV‐control‐miRNA into C26 tumour bearing mice. Despite a four‐fold reduction in circulating serum levels in the SAA1/2 KD group, SAA1 was still highly up‐regulated in tumour bearing mice and no differences were observed in cachexia progression. In order to define novel liver‐secreted factors that can potentially impact cachexia development, we integrated hepatocyte specific RNAseq and serum proteome analyses of C26 cachectic animals and observed a high degree of overlap. We are currently functionally characterizing a panel of these hepatocyte‐secreted proteins in order to investigate their potential adipocyte lipolysis and/or myotube atrophy‐mediating properties. These studies might contribute to a better understanding of the pathophysiology of CC and the liver‐specific contribution to its development.

**2-06**

**The use of proton pump inhibitors might increase symptoms of cachexia in patients with chronic illness**

**Paulien Vinke** ^1,2^ and Klaske van Norren^1^

^1^ *Nutritional Biology, Division of Human Nutrition and Health, Wageningen University, Wageningen, The Netherlands;* ^2^ *University Clinic of Cardiology, Heart Center Leipzig, Leipzig, Germany*

**Introduction:** Long‐term use of proton pump inhibitors (PPIs, e.g. omeprazole), is becoming increasingly common in patients with cachexia‐related chronic diseases, as cancer, COPD, and heart failure.

**Methods:** To determine mechanistically, with a literature search, whether the use of PPIs may affect the development of cachexia or the health status of cachectic patients.

**Results:** PPIs increase the pH in the lumen, which changes the microbiota composition and decreases the activity of TRPM6, a major transporter in magnesium metabolism. Decreased TRPM6 activity may lead to lower magnesium absorption from the gut. Low free levels of magnesium, in turn, compromise several processes that are relevant in cachexia. For example, magnesium is necessary for activation of vitamin D. A deficiency in active vitamin D is linked to musculoskeletal and immune function. Hypomagnesaemia can also cause muscle weakness, muscle cramps, and fatigue. Moreover, a PPI‐induced change in the microbiome composition may lead to a dysfunctional lipid and energy metabolism. Finally, both microbiota dysfunction and hypomagnesaemia can increase inflammation.

**Conclusions:** Based on a thorough literature search, it can be concluded that PPIs can contribute to hypomagnesaemia, vitamin D deficiency, inflammation, and a change in microbiota. These are properties that all can have an impact on the development of cachexia. Therefore, more research on potential unwanted side‐effects of PPI use in diseases related to development of cachexia is necessary. In these studies, it is recommended to screen for PPI‐induced nutritional deficiencies and to study the effect of treating those deficiencies.

**2-07**

**Mitochondrial respiratory rates in abdominal muscle tissue of colon cancer patients**

**Miranda van der Ende** ^1,2^, Sander Grefte^1^, Renger F. Witkamp^2^, Jaap Keijer^1^ and Klaske van Norren^2^

^1^ *Human and Animal Physiology, Wageningen University & Research, Wageningen, The Netherlands;* ^2^ *Division Human Nutrition, Wageningen University & Research, Wageningen, The Netherlands*

During extensive literature research, we found that gene and protein expression of muscle mitochondrial dynamics are altered during cancer cachexia in animal models [1]. Therefore, we will measure the oxygen consumption of the rectus abdominis in a subset of the COMUNEX study, an observational study designed to gain insight in changes in muscle gene expression, body composition, muscle function, and muscle metabolism of colon cancer patients. To date, 21 primary colon cancer patients (age 68 ± 7.01) and 3 liver metastasized patients (age 58 ± 15.56) were included. For the most recently included patients of this population (*N* = 8) oxygen consumption was measured. Tissue of the rectus abdominis was collected in BIOPS buffer during surgery. Oxygen consumption was measured in permeabilized muscle tissue (saponin 50 μg/mL 30 min) using the OROBOROS respirometer. Injection protocol (end concentrations): 5 mM pyruvate, 2 mM malate, 10 mM glutamate, 5 mM ADP + Mg^2+^, 10 μM cytochrome C, 10 mM succinate, 5 mM ADP + Mg^2+^, 5 μM oligomycin, titration with 1 μL steps of 1 mM CCCP, 2.5 μM rotenone, and 5 μM antimycin A. No control patients were included, and therefore, no comparison with ‘healthy’ patients could be made at this time point of the study. On average, the patients showed a 16% ± 12% increase in oxygen consumption after cytochrome C injection. A typical trace is shown in Figure 1A. Additionally, Figure 1B depicts the average of different states and shows that the ET‐NS state is lower than the OXPHOS‐NS state which is remarkable. We hypothesize that respiration of the rectus abdominis muscle tissue of cancer patients will be overall lower than the respiration of the tissue from controls (*N* = 8, scheduled coming months). Nonetheless, currently, it is too early to draw any conclusions.

**Figure 1:**
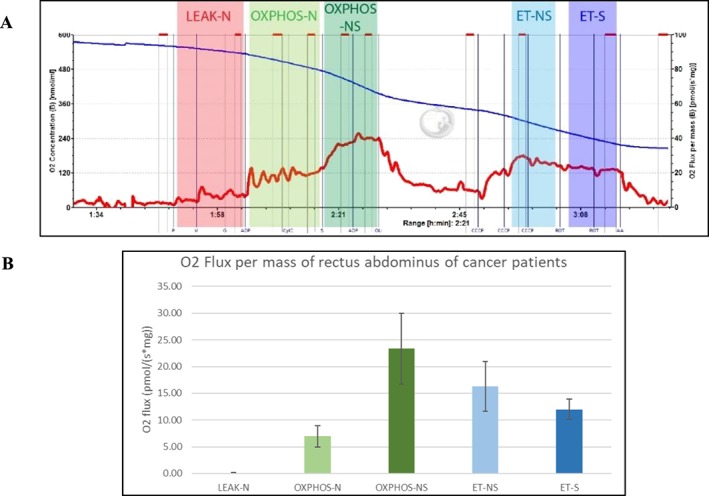
O2 Flux per mass of rectus abdominis of cancer patients. (A) a typical trace obtained by the OROBOROS respirometer. (B) the average of O2 flux of different states. *N* = 8 all measurements are in duplo. Data are visualized as mean with SD.

**References**

1. van der Ende, M., et al., Mitochondrial dynamics in cancer‐induced cachexia. *Biochim Biophys Acta*, 2018.

**2-08**

**Tumour organoids as a novel model to study cancer‐induced cachexia in pancreatic cancer**

**Rianne D.W. Vaes** ^1^, Ramon Langen^2^, Merel R. Aberle^1,3^, Jorne Ubachs^1,4^, David P.J. van Dijk^1^, Tessa T.J. Welbers^1^, Lara Heij^5^, Steven W.M. Olde Damink^1,5^ and Sander S. Rensen^1^

^1^ *Department of Surgery and NUTRIM School of Nutrition and Translational Research in Metabolism, Maastricht University, Maastricht, The Netherlands;* ^2^ *Department of Respiratory Medicine and NUTRIM School of Nutrition and Translational Research in Metabolism, Maastricht University, Maastricht, The Netherlands;* ^3^ *Department of Pharmacology and Toxicology and NUTRIM School of Nutrition and Translational Research in Metabolism, Maastricht University, The Netherlands;* ^4^ *Department of Obstetrics and Gynaecology and GROW—School of Oncology and Developmental Biology, Maastricht University Medical Centre, Maastricht, The Netherlands;* ^5^ *Department of General, Visceral and Transplantation Surgery, RWTH University Hospital Aachen, Aachen, Germany*

**Background:** The majority of patients with pancreatic cancer develop cachexia, which is characterized by progressive muscle loss. However, the mechanisms underlying cancer cachexia development and progression remain elusive. Pancreatic tumour organoids are *in vitro* 3D organ‐like structures that retain specific structures and pathophysiological characteristics of the original tumour. We aimed to establish pancreatic tumour organoids from well‐phenotyped cachectic patients to study their cachexia‐inducing properties.

**Methods:** Organoids were established from tumour tissue of cachectic (*n* = 5) and non‐cachectic (*n* = 3) pancreatic cancer patients. A comprehensive pre‐operative assessment of cachexia‐related parameters was performed (i.e. nutritional status, physical performance, body composition, and inflammation). Tumour‐ and cachexia‐related characteristics of the tumour organoids were analysed using histological stainings, sequencing, and RT‐qPCR. C2C12 myoblasts were exposed to organoid conditioned medium (CM) and mRNA expression of myogenic markers and myosin heavy chain (MyHC) isoforms was analysed by RT‐qPCR.

**Results:** Organoids presented typical features of malignancy similar to the primary tumour (i.e. nuclear enlargement, nucleoli, mitosis, apoptosis, and mutated *KRAS* and/or *TP53*). mRNA expression of known cachexia‐related factors was highly variable among the organoid lines. Strikingly, *IL‐8* (1.4‐fold, *P* = 0.01), *LIF* (1.9‐fold, *P* = 0.0043), *GDF15* (2.3‐fold, *P* < 0.001), and *MIF* (3.0‐fold, *P* < 0.001) expressions were significantly increased in organoids from cachectic versus non‐cachectic patients. Unexpectedly, exposure of C2C12 myoblasts to organoid CM accelerated myogenic differentiation, as evident from reduced expression of *PAX7* (2.0‐fold) and *CCND1* (1.7‐fold), and increased expression of *MYOG* (1.6‐fold), *MYMK* (2.8‐fold), and *MCK* (3.9‐fold) early after initiation of differentiation. Subsequent myotube formation was accompanied by increased expression of MyHC‐2b (8.7‐fold) and decreased expression of MyHC1 (1.3‐fold), consistent with the shift from slow‐ to fast‐twitch muscle fibres often observed in cachectic patients.

**Conclusions:** Tumour organoid‐derived factors from cachectic patients with pancreatic cancer affect both differentiation and maturation of C2C12 myoblasts. Organoids represent a promising model to uncover mechanisms underlying cancer‐induced cachexia.

**3-01**

**Elevated plasma growth differentiation factor‐15: a powerful prognostic factor that associates with skeletal muscle loss in patients with metastatic non‐small cell lung cancer**

**Sean Kazemi** ^1^, Harald Becher^2^, Charles Butts^3^, Naveen S. Basappa^3^, Michael Smylie^3^, Anil Abraham Joy^3^, Randeep Sangha^3^, Andrea Gallivan^1^, Peter Kavsak^4^, Quincy Chu^3^ and Vickie E. Baracos^1^

^1^ *Department of Oncology, Division of Palliative Care Medicine;* ^2^ *Department of Medicine, Division of Cardiology, Alberta Cardiovascular and Stroke Research Centre;* ^3^ *Department of Oncology, Division of Medical Oncology, Cross Cancer Institute, University of Alberta, Edmonton, Canada;* ^4^ *Department of Pathology and Molecular Medicine, McMaster University, Hamilton, Canada*

**Introduction:** Growth differentiation factor‐15 (GDF‐15) is a putative cancer cachexia and prognostic biomarker. We aimed to assess GDF‐15 versus C reactive protein (CRP), a conventional cachexia biomarker in patients with non‐small cell lung cancer (NSCLC).

**Methods:** Treatment naïve patients [*n* = 46, 64.5 ± 7.7 years old, 22 male, body mass index (BMI) = 26.4 ± 5.7 kg/m^2^, 19 patients >5% weight loss] with metastatic NSCLC were studied. All patients were candidates for first‐line carboplatin‐based therapy. Plasma collection, computed tomography imaging, and tumour progression analysis were performed before chemotherapy and 2–4 weeks after 4th cycle of chemotherapy (i.e. 112 ± 6 days afterwards).

**Results:** BMI and CT‐defined skeletal muscle area decreased over time [26.4 ± 5.7 kg/m^2^ to 25.5 ± 5.3 kg/m^2^, *P* < 0.001 and 130.2 ± 37.2 cm^2^ to 120.6 ± 30.6 cm^2^, *P* < 0.001, respectively). GDF‐15 (ng/mL) [median, IQs: 3.8 (2.5–6.3) increased to 5.9 (3.4–12.5), *P* < 0.001] and CRP (mg/L) [8.5 (3.5–24.1) increased to 13.8 (4.6–38.7), *P* = 0.007] elevated over time. Baseline CRP > 10 mg/L and baseline GDF‐15 > median value (3.8 ng/mL) were selected as cut‐off values. Of 23 patients with GDF‐15 ≤ 3.8 ng/mL, 17 patients had CRP ≤10 (74%); however, 65% of patients with GDF‐15 > 3.8 ng/mL had concurrent CRP > 10 (*P* = 0.017). Baseline GDF‐15 (ng/mL) in sarcopenic patients (*n* = 25) was higher compared to non‐sarcopenic patients [4.8 (3.7–8.9 vs. 3.2 (1.8–4.2), *P* < 0.001]; however, CRP was not different by sarcopenia status (*P* = 0.28). In univariate analysis, tumour progression (HR = 3.8, *P* < 0.001), GDF‐15 > 3.8 ng/mL (HR = 10.4; *P* < 0.001) and CRP > 10 mg/L (HR = 3.0, *P* = 0.003) were related to overall survival (OS); however, in multivariate analysis, GDF‐15 > 3.8 ng/mL had the strongest independent effect (CRP > 10 mg/L, HR = 1.3, *P* = 0.6; GDF‐15 > 3.8 ng/mL, HR = 9.5, *P* < 0.001; tumour progression, HR = 3.5, *P* = 0.003). Median OS of patients with GDF‐15 > 3.8 ng/mL versus patients with GDF‐15 ≤ 3.8 ng/mL: 177 days versus 617 days (*P* < 0001).

**Conclusions:** Elevated GDF‐15 is associated with hyper‐inflammation, skeletal muscle loss, and poor OS in metastatic NSCLC patients.

**3-02**

**Early changes in muscle and adipose tissue in stage IV non‐small cell lung cancer treated with immune‐checkpoint inhibitors**

**Juliette H.R.J. Degens** ^1^, Anna C.H. Willemsen^1,2^, Hester Gietema^3^, Lizza Hendriks^4^, Anne‐Marie C. Dingemans^5,6^ and Annemie W.J. Schols^1^

^1^ *Department of Respiratory Medicine, NUTRIM School of Nutrition and Translational Research in Metabolism, Maastricht University Medical Center, Maastricht, The Netherlands;* ^2^ *Department of Medical Oncology, GROW—School of Oncology and Developmental Biology, Maastricht University Medical Center, The Netherlands;* ^3^ *Department of Radiology, Maastricht University Medical Center, Maastricht, The Netherlands;* ^4^ *Department of Respiratory Medicine, Maastricht University Medical Center, Maastricht, The Netherlands;* ^5^ *Department of Respiratory Medicine, Erasmus Medical Center, Rotterdam, The Netherlands;* ^6^ *Department of Respiratory Medicine, GROW—School for Oncology and Developmental Biology, Maastricht University Medical Center, Maastricht, The Netherlands*

**Introduction:** Early loss of skeletal muscle (SM) during chemotherapy has shown to be poor prognostic for overall survival (OS) in stage IV non‐small cell lung cancer (NSCLC) [1]. Treatment options have expanded with immune checkpoint inhibitors (ICI). Data on changes of SM and adipose tissue (AT) during treatment with ICI in stage IV NSCLC are lacking.

**Methods:** This was a single centre study. All patients were treated with ICI between June 2015 and December 2018. SM, subcutaneous adipose tissue (SAT), and visceral adipose tissue (VAT) were characterized at the first lumbar level on CT images in stage IV NSCLC. CT scans of 130 patients were obtained before the start of ICI treatment (baseline) and after 6 and 12 weeks. The contribution of changes in different body compartments to OS was assessed.

**Results:** Between baseline and 12 weeks, a significant loss was observed of SM cross‐sectional area (CSA) (−2.4 ± 9.8%, *P* = 0.003), VAT CSA (−9.5 ± 30%, *P* = 0.001), and SAT CSA (−10 ± 26.3%, *P* = 0.001) but not in muscle attenuation. Mean weight change was −0.2% (±4.9, NS). Median OS was significantly shorter among patients losing VAT (14 vs. 25 months; HR 2.19, 95% CI 1.26–3.81, *P* < 0.05) and SAT (11 vs. 20 months; HR 2.91, 95% CI 1.53–5.55, *P* < 0.05). In the subgroup of 90 patients without progression of disease within 12 weeks, multivariate analysis showed that loss of VAT and SAT CSA remained significantly associated with poor OS, independent from age, gender, smoking‐status, WHO‐PS, and pre‐existent weight loss.

**Conclusions:** Loss of adipose tissue in stage IV NSCLC patients treated with immune checkpoint inhibitors is associated with poor prognosis.

**References**

1. Degens J, Sanders KJC, de Jong EEC, Groen HJM, Smit EF, Aerts JG, Schols A, Dingemans AC: The prognostic value of early onset, CT derived loss of muscle and adipose tissue during chemotherapy in metastatic non‐small cell lung cancer. *Lung Cancer* 2019, **133**:130‐135.

**3-03**

**Dietary intake and weight management by patients with stage I–III colorectal cancer receiving systemic anticancer treatment and at risk of cachexia: mixed methods research**

**Jane B. Hopkinson** ^1^, Catherine Kazmi^1^, Sally Wheelwright^2^, Clare Shaw^3^ and Jayne Elias^4^

^1^ *Cardiff University, Cardiff, Wales, UK;* ^2^ *University of Southampton, Southampton, UK;* ^3^ *Royal Marsden Hospital, London, UK;* ^4^ *Velindre University NHS Trust, Cardiff, UK*

**Introduction:** This research investigated factors affecting patients' self‐management of dietary intake during systemic anti‐cancer treatment for stage I–III colorectal cancer.

**Methods:** The research was a mixed‐methods study conducted at a cancer centre in the UK. A questionnaire to assess eating problems, which included the Patient‐Generated Subjective Global Assessment (PG‐SGA), was administered to patients with stage I–III colorectal cancer in treatment (*n* = 92). Telephone interviews were then conducted with a maximum variation sample of these patients (*n* = 20), to explore factors influencing nutritional self‐care. The survey data were analysed using descriptive statistics and the interview data using framework analysis. New insights into patients' nutritional self‐care during treatment were then generated by synthesis of results.

**Results:** The survey was completed by 52 patients (response rate 56.5%). A majority were assessed to be at nutritional risk [34/52 (65%)] of whom one third [19/52 (37%)] had lost more than 5% body weight over a 6 month period, thus met criteria for cachexia. Despite this, only a minority were concerned about their dietary intake (19%) or weight (21%). Eleven of those who had lost more than 5% body weight [11/19 (58%)] had lost weight during treatment. At interview, patients often spoke about the association of processed meat and/or obesity with colorectal cancer. Many had cut processed meat from their diet. However, they were ambivalent about or resistant to nutritional advice offered by oncology staff, particularly if they were gaining weight. Uncertainty was expressed about how to respond to contradictory sources of dietary advice, and justifications were provided for abdication of responsibility for nutritional self‐care during treatment.

**Conclusions:** The majority of study participants did not recognize or act on the risk of malnutrition and cachexia. There is therefore potential for psychoeducation to activate nutritional self‐care with implications for treatment tolerance and outcomes in patients with colorectal cancer.

**3-04**

**A sarcopenia‐based predictive model of complications after resections of the colon**

**Vladimir K. Lyadov** ^1,2^ and Vitalii G. Polushkin^3^

^1^ *City Clinical Cancer Hospital N1, Moscow, Russia;* ^2^ *Chair of Oncology, Russian Medical Academy of Continuous Professional Education, Russia;* ^3^ *Moscow Center of Rehabilitation Medicine, Russia*

**Introduction:** Colorectal cancer is one of the major contributors to cancer mortality with surgery being the most efficient treatment modality. However, colorectal resections are associated with high post‐operative morbidity and mortality, especially in the elderly. We aimed to develop a predictive formula in order to decrease the morbidity rate.

**Methods:** We analysed the results of 347 elective resections of the colon, performed between 2009 and 2015 in the Department of Surgical Oncology (State Medical and Rehabilitation Center, Moscow, Russia), including 96 in the elderly group (75 years and older, 28%) and 164 (47%) in patients in the age of 60–74. Our primary endpoint was the number of severe post‐operative complications (stage III–V according to Clavien–Dindo classification). Univariate analysis included sex, age, BMI, sarcopenia, or sarcopenic obesity (evaluated by CT scan on the L3 level analysis with Slice‐O‐Matic software), nutritional deficiency according to NRS2002 score, Charlson and CIRS co‐morbidiy scores, ASA, ECOG, laparoscopic surgery, surgeon experience, OP‐duration, blood loss, and enhanced recovery protocol. We used age specific cut‐offs for sarcopenia: 52.4 cm^2^/m^2^ in men and 39.5 cm^2^/m^2^ in women. Factors found to be predictive in the univariate analysis were included in the multivariate analysis to develop a nomogram.

**Results:** Three factors appeared to predict severe complications in the multivariate analysis: male sex, the presence of sarcopenia and age. Every decade of life after 40 added 1 point to the developed formula. The model afforded to predict the probability of severe complications in the range of <5% to >45% in male patients and <5% to >33% in female patients.

**Conclusions:** Sarcopenia, elderly age, and male sex appeared to predict the development of grade III–V complication after elective resections of the colon in cancer patients. An external validation of the model is necessary.

**3-05**

**International validation of the European Organisation for Research and Treatment of Cancer (EORTC) QLQ‐CAX24, a questionnaire to assess health‐related quality of life in cancer patients with cachexia**

**Sally Wheelwright** ^1^, Jane B. Hopkinson^2^, Kim Cocks^3^, Anne‐Sophie Darlington^1^, Deborah Fitzsimmons^4^, Samantha Sodergren^1^, Tora S. Solheim^5^, Trude R. Balstad^5^ and Colin Johnson^1^ and on behalf of the EORTC Quality of Life Group and QLQ‐CAX24 Module Development Collaborators

^1^ *University of Southampton, UK;* ^2^ *Cardiff University;* ^3^ *Adelphi Values;* ^4^ *Swansea University;* ^5^ *NTNU—Norwegian University of Science and Technology & Trondheim University Hospital, Norway*

**Introduction:** A validated health related quality of life (HRQOL) questionnaire is vital for the assessment of new treatments for cancer cachexia and could also aid clinical management. The QLQ‐CAX24 questionnaire was developed to supplement the QLQ‐C30 (core EORTC questionnaire, applicable to all cancer patients) to assess HRQOL in people with cancer cachexia. It includes five sub‐scales (food aversion, eating and weight loss worry, eating difficulties, loss of control, and physical decline) and single items covering the HRQOL issues identified as the most important and relevant by people with cancer cachexia. This multi‐centre, international study validates the QLQ‐CAX24 and assesses the acceptability to patients.

**Methods:** Patients from 13 countries, with any cancer diagnosis, completed the QLQ‐C30, QLQ‐CAX24, a nutrition impact symptom checklist and a debriefing interview. Questionnaires were repeated 1 week later by 52 clinically stable patients (test–retest analysis) and 1 month or later by 119 patients (responsiveness to change analysis). All debrief interviews were reviewed. Standard psychometric analyses for questionnaire validation, following EORTC guidelines, were performed.

**Results:** The sample included 609 patients (mean age 61 years, 346 with cachexia syndrome, 152 refractory cachexia, 68 no weight loss at baseline, 43 unknown cachexia status). The debriefing interviews demonstrated that the QLQ‐CAX24 was acceptable to patients. Multi‐trait scaling showed all items correlated sufficiently with their hypothesized subscales, and internal reliability was acceptable. Test–retest reliability and responsiveness to change were adequate. The QLQ‐CAX24 differentiated between cachexia syndrome and refractory cachexia groups and those with many versus few nutrition impact symptoms.

**Conclusions:** The QLQ‐CAX24 is a validated HRQOL questionnaire, available in 14 languages, for patients across the cachexia continuum. The questionnaire can now be used in clinical trials. Its use in routine clinical practice, to help identify and address important HRQOL concerns in cancer patients with cachexia, should be evaluated.

**3-06**

**Current knowledge about eating‐related distress among advanced cancer patients with cachexia and their family members and expected effectiveness of integration of palliative, supportive, and nutritional care for their distress**

**Koji Amano** ^1^, Vickie E. Baracos^2^ and Jane B. Hopkinson^3^

^1^ *Department of Palliative Medicine, Osaka City General Hospital, Osaka, Japan;* ^2^ *Division of Palliative Care Medicine, Department of Oncology, University of Alberta, Cross Cancer Institute, Edmonton, Alberta, Canada;* ^3^ *School of Healthcare Sciences, College of Biomedical and Life Sciences, Cardiff University, Cardiff, Wales, UK*

**Introduction:** Many advanced cancer patients with cachexia and their family members suffer from eating‐related distress, which encompasses patients' struggle to eat, psychosocial consequences of eating problems, and disturbance in family relationships. Negative impacts of cachexia can be mitigated with nutritional care, as well as symptom management. Additionally, patients and family members require psychosocial support and education to understand and cope with such impacts. An integrated approach by health care professionals can alleviate eating‐related distress, improve quality of life, reduce interpersonal conflicts, and alter perceptions of nutritional neglect for patients and family members. However, there is a paucity of studies investigating eating‐related distress.

**Methods:** Narrative review to describe what is known about their eating‐related distress and the role of integrated palliative, supportive, and nutritional care.

**Results:** We categorized knowledge of eating‐related distress in patients and family members as (i) nutrition impact symptoms, (ii) eating‐related distress and perceived need for nutritional support in patients, (iii) eating‐related distress and perceived need for nutritional support in family members, (iv) conflicts over food between patients and family members, (v) role of palliative and supportive care, (vi) role of nutritional care, and (vii) integration of palliative, supportive, and nutritional care.

**Conclusions:** We found limited information about eating‐related distress among patients and family members. There are no well‐developed educational tools or psychosocial interventions to alleviate their distress. There is a lack of validated tools for the clinical assessment of eating‐related distress and its response to interventions. More robust evidence regarding nutrition impact symptoms and their association with distress is needed. To inform and support patients and family members to cope effectively, health care professionals must appreciate the complexities of eating‐related distress. We propose that this will be facilitated by close coordination of palliative, supportive, and nutritional care.

**3-07**

**Sexual dimorphism in muscle depletion during end of life stage in cancer patients**

**Naoharu Mori** ^1^, Keisuke Maeda^1^, Tomoyuki Nonogaki^2^, Ryoko Kato^2^, Tomoko Kimura^3^ and Yuria Ishida^3^

^1^ *Palliative Care Center;* ^2^ *Department of Pharmacy;* ^3^ *Department of Nutrition, Aichi Medical University, Nagakute, Japan*

**Background:** It has been reported that muscle decline in cancer patients differs by gender. However, such decline during end of life stage in cancer patients is unclear. Here, we investigated sexual dimorphism in muscle decline of terminal cancer patients.

**Methods:** A retrospective study of all adult solid cancer patients referred to our palliative care team from July 2017 to April 2018 was conducted. We analysed associations between survival time and; muscle mass [psoas muscle index (PMI)]; density of psoas muscle; pinch grip strength (PGS); and inflammation [C‐reactive protein (CRP)], at first consultation. Cox's proportional hazards model was used to estimate the median mortality hazard ratio (HR) of each muscle variable.

**Results:** Of the 71 solid cancer patients (45% male, median age 68 years), median survival were 68 (range 35–291, 95% CI) and 117 (range 42–209, 95% CI) days for male and female, respectively. After adjustment for age, gender, and CRP as potential confounders, loss of PMI (HR 0.997, range 0.995–0.999, 95% CI, *P* = 0.009) and PGS (HR 0.613, 0.437–0.859, 95% CI, *P* = 0.004) independently predicted overall survival time. In gender‐based subgroup analysis, PMI was associated with longer survival in male patients but not female patients (male HR 0.997, 0.995–0.999, 95% CI, *P* = 0.026; female HR 0.998, 0.995–1.002, 95% CI, *P* = 0.272). PGS was associated with longer survival in both (male HR 0.664, 0.471–0.934, 95% CI, *P* = 0.019; female HR 0.535, 0.312–0.916, 95% CI, *P* = 0.022).

**Conclusions:** In terminal cancer patients, sexual dimorphism in muscle decline was observed. Decreased muscle mass is a prognostic factor of survival length in male patients but not female patients. On the other hand, muscle weakness is a prognostic factor of survival length for advanced cancer in both sexes.

**3-08**

**A sexual dimorphism linking cancer cachexia with obesity**

**Erin E. Talbert** ^1,2,3^, Kevin Zambrano^1^, Terence M. Williams^1,4^, Zarine Shah^1,5^, Carl R. Schmidt^1,6,7^, Mary E. Dillhoff^1,6^ and Denis C. Guttridge^1,2,3^

^1^ *Arthur G. James Comprehensive Cancer Center Cancer Cachexia Program;* ^2^ *Darby Children's Research Institute;* ^3^ *Department of Pediatrics and the Hollings Cancer Center, Medical University of South Carolina, Charleston, SC, USA;* ^4^ *Department of Radiation Oncology;* ^5^ *Department of Radiology;* ^6^ *Division of Surgical Oncology, The Ohio State University, Columbus, OH, USA;* ^7^ *Department of Surgery, West Virginia University, Morgantown, WV, USA*

**Introduction:** Despite decades of research, pancreatic adenocarcinoma remains a devastating disease. While the difficulty of detection and aggressive biology of this tumour are significant contributors to the high mortality rates of this disease, body weight loss, and particularly the loss of skeletal muscle mass, also contributes to poor survival of patients with pancreatic cancer.

**Methods:** Because pancreatic cancer incidence and average body weight losses are similar between men and women, little attention has been paid to how cachexia may differ between the sexes. We sought to investigate sex differences in the clinical and biological data contained in The Ohio State University Comprehensive Cancer Center Pancreatic Cancer Cachexia Biobank. Consistent with an international consensus, patients were classified as cachectic if they had lost greater than 5% of their pre‐illness body weight.

**Results:** Consistent with previous reports, we found cachectic patients (*n* = 59) had higher pre‐illness body mass indices (BMI) than weight‐stable cancer patients (*n* = 30). However, when men and women were assessed separately, only cachectic women tended to have higher pre‐illness BMIs than their weight‐stable counterparts. Importantly, cachectic men and women had similar changes in BMI. To further characterize this potential sex difference, CT scans were analysed for a subset of patients. While there were no differences between cachectic and weight stable men or women in estimated fat mass, we did identify an interaction effect in fat free mass, suggesting a sex‐dependent effect of cachexia in pancreatic cancer patients. RNA sequencing data from muscle support this dissimilarity between men and women, as clear gene expression clustering is present in male patients, yet absent in female patients.

**Conclusions:** While there are caveats to this work, including the limited power and cross‐sectional nature of the study, these data provide strong rationale for consideration of sex as a biological variable in future cancer cachexia studies.

**3-09**

**Inflammatory pathways involved in the gut‐brain axis in cachexia**

**Xiaolin Li**, Mieke Poland, Tosca Holtrop and Klaske van Norren

*Wageningen University, Wageningen, The Netherlands*

**Background:** Cancer‐related cachexia is characterized by skeletal muscle wasting and systemic inflammation. The gut and hypothalamus have been indicated to play a key role. However, the mechanistic details remain largely unexplored. To determine the mechanisms behind the cross talk between the tumour, the gut, and the hypothalamus that lead to muscle wasting in cachexia.

**Methods:** To imitate the influence of decreased integrity of the gut, sub‐inflammatory levels (31.6 ng/mL) of the gut‐derived bacterial compound lipopolysaccharides (LPS) were added to hypothalamic (HypoE‐N46) cells. These cells were incubated with the secretome of cachexia inducing tumour cells from murine colon (C26). Subsequently, hypothalamic cells were exposed for 24 h to LPS in combination with tumour‐secretome or a mimic of this secretome (110 pg/mL IL6, 30 000 pg/mL MCP‐1, 100 pg/mL LIF, 6500 pg/mL PGE2). Effects on inflammation were assessed by measuring Il‐6, MCP‐1, and LIF secretion using a commercial ELISA.

**Results:** The IL6‐release of hypothalamic cells reached 1100 pg/mL when incubated with the combination of the C26 tumour‐secretome and 31.6 ng/mL of LPS. This was several magnitudes higher than the release of hypothalamic cells incubated with C26 tumour‐secretome or LPS separately (50 and 365 pg/mL, respectively, *P* < 0.05). A similar finding was observed with the C26 tumour‐secretome mimic (separately: 26 pg/mL, combination LPS and tumour‐secretome mimicking medium: 940 pg/mL, *P* < 0.05). The MCP‐1 and LIF release of the hypothalamic cells showed no or less additive effects of the tumour‐secretome and LPS, indicating that for secretion of these inflammatory mediators, different pathways might be involved.

**Conclusions:** These data indicate that the inflammatory hypothalamic response to a tumour can be significantly more pronounced in the presence of low levels of gut‐derived bacterial compounds like LPS. This increased activation seems to be associated with the inflammatory mediators secreted by the tumour.

**3-10**

**SRF role as a mechano‐transductor in response to exercise in cancer cachexia**

**Hassani Medhi** ^1,2^, Benoni Alexandra^1^, Xue Zhigang^2^, Sotiropoulos Athanassia^3^, Parlakian Ara^2^, Li Zhenlin^2^, Adamo Sergio^1^ and Coletti Dario^1,2^

^1^ *DAHFMO Unit of Histology and Medical Embryology, and Interuniversity Institute of Myology, Sapienza University of Rome, Italy;* ^2^ *Department of Biological Adaptation and Ageing B2A (CNRS UMR 8256—INSERM ERL U1164—UPMC P6), Sorbonne University Paris 6, France;* ^3^ *Institut National de la Santé et de la Recherche Médicale, U810, and Faculté de Médecine Necker‐Enfants Malades, Université Paris 5, France*

**Introduction:** Recent studies showed that physical activity increased survival in cancer patient and animal models of cancer cachexia. The underlying mechanisms, however, are still largely unknown.

**Methods:** To identify signalling pathways involved in exercise‐dependent maintenance of muscle mass and function in cachexia, we investigated the role of serum response factor (SRF)—a transcription factor playing a pivotal a role in muscular growth, differentiation and regeneration—in C26‐bearing mice in the absence or presence of voluntary exercise (wheel running).

**Results:** SRF levels are decreased at protein level in cachexia. Consistently, a decrease in the expression of SRF target genes such as MyoD and SK‐actin occurs in C26‐bearing mice, suggesting a decrease of SRF transcriptional activity. These tumour effects were counteracted by wheel running and associated to the rescue of muscle mass and function. However, a minimum amount of exercise (2 km/day) is necessary to keep SRF levels elevated in cachexia over a threshold which is necessary to exert beneficial effects. SRF levels inversely correlate with wasting in mice, suggesting that SRF play a role in maintaining body mass (mostly accounted for by muscle mass). We also observe the recruitment of nuclei within the muscle fibres in response to exercise, which could contribute to muscle homeostasis and is consistent with the previously observed opposite effects of tumour and exercise on MyoD and Pax7 expression.

**Conclusions:** Our results suggest that physical activity rescues SRF expression as well as its transcriptional activity, highlighting the importance of genetic activation induced by skeletal muscle activity for muscle rescue and homeostasis. These effects could be extended to the fibre microenvironment, including myogenic stem cell activity.

**3-11**

**Toward the identification of receptor for advanced glycation end‐products (RAGE) as a muscle biomarker of cancer cachexia**

**Aleksandra Vukasinovic** ^‡, 1,4^, Sara Chiappalupi^1,4" noteRef="jcsm12513-subcmp-0034-note-0002^, Guglielmo Sorci^1,4^, Laura Salvadori^1,4^, Maurizio Muscaritoli^2^, Paola Costelli^3,4^, Rosario Donato^1,4" noteRef="jcsm12513-subcmp-0034-note-0001^ and Francesca Riuzzi^1,4" noteRef="jcsm12513-subcmp-0034-note-0001^

^1^ *Department of Experimental Medicine, University of Perugia, Perugia, Italy;* ^2^ *Department of Department of Clinical Medicine; Sapienza University of Rome, Italy;* ^3^ *Department of Clinical and Biological Sciences; University of Torino, Italy;* ^4^ *Interuniversity Institute of Myology (IIM), Perugia, Italy*

^†^These authors equally contributed to the work.

^‡^These authors shared senior authorship.

**Introduction:** Cachexia is a debilitating syndrome affecting more than 50% of patients with advanced cancer. Its major clinical feature is skeletal muscle atrophy leading to pronounced weight loss, reduced quality of life, and poor prognosis. The identification of valuable biomarkers of early cachexia is of great importance to identify the patients at risk of cachexia and to treat patients in the reversible phase of the disease (Porporato *et al*., *Oncogenesis* 2016, Loumaye‐Thissen, *Clin Biochem* 2017). RAGE (receptor for advanced glycation end‐products) signalling concurs to skeletal muscle development and homeostasis (Riuzzi *et al., JCSM* 2018); however, in cancer conditions, RAGE hyperstimulated by high levels of its ligands leads to muscle wasting, sustains inflammation, and reduces survival of mice (Chiappalipi *et al*., submitted). Here, we investigated whether RAGE might represent a biomarker of cachexia.

**Methods:** We analysed RAGE expression in muscle tissue of different tumour‐bearing mice in the absence or presence of endurance exercise and correlated it with myofiber CSA and hallmarks of atrophy (body and muscle weights, protein degradation, and activation of the proteolytic systems). We performed RAGE expression analysis in muscle biopsies of cancer patients.

**Results:** We found that (i) Lewis lung carcinoma (LLC)‐bearing C57Bl/6 mice and colon adenocarcinoma (C26‐ADK)‐bearing BALB/c mice express RAGE in myofibres in coincidence with reduced body and muscle weight and induction of proteolysis; (ii) an inverse relationship exists between RAGE expression in muscles, tumour masses, and the beneficial effects of endurance exercise in LLC‐bearing mice; (iii) RAGE expression increases in muscles during cachexia progression; (iv) LLC or melanoma A375 cells injected in athymic‐nude mice are not able to induce neither cachexia nor RAGE expression in muscle; and (v) muscles of cachectic patients express higher amounts of RAGE than non‐cachectic subjects.

**Conclusions**: RAGE might represent a muscle biomarker of the cachectic stage.

**3-12**

**Musclin, a myokine induced by aerobic exercise, retards muscle atrophy during cancer cachexia**

**Andrea D. Re Cecconi** ^1^, Mara Forti^1^, Michela Chiappa^1^, Luigi Cervo^1^, Luca Beltrame^2^, Sergio Marchini^2^ and Rosanna Piccirillo^1^

^1^ *Department of Neurosciences, Mario Negri Institute for Pharmacological Research IRCCS, Milan, Italy;* ^2^ *Department of Oncology, Mario Negri Institute for Pharmacological Research IRCCS, Milan, Italy*

Physical activity improves the prognosis of cancer patients, partly by contrasting the associated muscle wasting (cachexia), through still unknown mechanisms. Since aerobic exercise seems to be the most effective to preserve muscles during cancer, we asked whether it promotes secretion of proteins by muscles (i.e. myokines) that may contrast cachexia. Media conditioned by PGC1‐α‐expressing myotubes, reproducing some metabolic adaptations of aerobic exercise, as increased mitochondrial biogenesis and oxidative phosphorylation, restrained caFoxO3‐induced proteolysis. Microarray analysis identified AREG, NppB, musclin, and FGF18 as myokines highly induced by PGC1‐α. Notably, only musclin tended to be low in muscle of mice with a rare human renal carcinoma, and it was reduced in plasma and in muscles of C26‐bearing mice and in atrophying myotubes, where PGC1‐α expression is impaired. So we electroporated tibialis anterior of C26‐bearing mice with musclin or (its receptor) Npr3‐encoding plasmids and found preserved fibre area, as a result of restrained proteolysis. Running protected C26‐bearing mice from cachexia, unchanging tumour growth, and rescued the C26‐induced down‐regulation of musclin in muscles and plasma. Musclin expression did not change in overloaded plantaris of mice, recapitulating partly muscle adaptations to anaerobic exercise. Furthermore, the expression of musclin, but not its receptor Npr3, was reduced in sarcopenic muscles of aged mice. Musclin might therefore be beneficial to cancer and perhaps also to sarcopenic old patients who cannot exercise for various reasons and may be added to the mechanisms by which aerobic exercise alleviates muscle wasting.

**3-13**

**Mechanisms of tumour metabolic reprogramming by Fn14 as a driver of cancer cachexia**

**Laura D. Osellame** ^1^, Ingrid J. Burvenich^2,3^, Zhipeng Cao^1^, Pierre Faou^1^, Harinda Rajapaksha^1^, Andrew M. Scott^2,3^ and Nicholas J. Hoogenraad^1^

^1^ *Department of Biochemistry and Genetics, La Trobe Institute for Molecular Science, La Trobe University, Melbourne, Victoria, Australia;* ^2^ *Olivia Newton‐John Cancer Research Institute, Melbourne, Victoria, Australia;* ^3^ *School of Cancer Medicine, La Trobe University, Melbourne, Victoria, Australia*

**Introduction:** Cachexia is a multifactorial metabolic syndrome characterized by weight loss, distal muscle, and fat wasting and is seen in ~50% of cancer patients. The molecular mechanisms of cachexia are poorly understood with no FDA approved treatments available. We have developed an antibody that targets the TWEAK receptor Fn14, on the tumour surface, which is able to abrogate symptoms of cachexia in syngeneic mouse models (Johnston *et al*., 2015, *Cell*). This anti‐Fn14 antibody (mAb 002) prolongs life by blocking body weight loss associated with cancer cachexia. We have shown that ^18^F‐FDG PET imaging links Fn14 to glucose metabolic pathways in tumours that induce cachexia. Here, we aim to further understand the role of Fn14 in tumour metabolism to drive cancer cachexia as well as the therapeutic effect of mAb 002.

**Methods:** Tumours from either vehicle or mAb 002 treated cachectic mice were subjected to large‐scale transcriptomic and proteomic profiling followed by gene ontology and pathway enrichment analysis. Metabolic assays were used to measure serum and tumour lactate levels. Digital droplet PCR was employed to assess metabolic response to cachexia in muscle and white/brown adipose tissue.

**Results:** ^18^F‐FDG PET imaging demonstrated increased glucose uptake in cachectic versus non‐cachectic tumour‐bearing mice, suggesting a higher glycolytic activity in tumours of cachectic mice. This occurs alongside an up‐regulation of pentose phosphate and nucleotide salvage pathways and a concomitant decrease in oxidative phosphorylation due to down‐regulation of mitochondrial ribosome subunits. Treatment of cachectic mice with mAb 002 prevents increased ^18^F‐FDG uptake, reduces tumour and plasma lactate levels, and blocks weight loss.

**Conclusions:** These results suggest that cachexia is driven by specific reprogramming of tumour metabolism, with downstream signalling events responsible for distal muscle and fat loss. This increase in glycolytic activity, body weight loss, and tumour growth can be reversed with anti‐Fn14 antibody treatment.

**3-14**

**Small‐molecule inhibition of MuRF1 attenuates skeletal muscle atrophy and dysfunction in tumour cachexia**

Volker Adams^1^, Victoria Gußen^1^, Sarah Werner^2^, Axel Linke^1^ and **Siegfried Labeit** ^3^

^1^ *Laboratory of Molecular and Experimental Cardiology, Heart Center Dresden, TU Dresden, Dresden, Germany;* ^2^ *University Clinic of Cardiology, Heart Center Leipzig, Leipzig, Germany;* ^3^ *Institut für Anästhesiologie und Operative Intensivmedizin, Universitätsklinikum Mannheim, Mannheim, Germany*

**Background:** Skeletal muscle atrophy is the most prominent phenotypic feature of cancer cachexia contributing to a reduced life expectancy and affecting quality of life. During tumour progression, the activation of catabolic processes, including the activation of muscle RING finger 1 (MuRF1), is well established. The Aim of the present study was to test the effect of a recently identified MuRF1 inhibitor, MCEMBL#205, on the development of muscle atrophy/dysfunction and molecular alterations.

**Methods:** Male C57BL/6N mice (*n* = 30) were included into the study. At an age of 6 to 8 months, the animals were randomized into the following groups (*n* = 10 in each group): (i) control group (Con), normal chow, no tumour; (ii) tumour group (Tu): inoculation with tumour cells and normal chow; (iii) tumour group on MCEMBL#205 (Tu‐205): inoculation with tumour cells and chow with MCEMBL#205. The chow containing Embl205 was started 3 days after inoculation with tumour cells (5*10^5^ cells per animal in 0.1 mL) into the right thigh. After 3 weeks, muscle force was determined, and tissue was harvested for molecular analysis.

**Results:** Functional analysis revealed impairment of muscle strength, which was attenuated by MCEMBL#205. This goes along with muscle mass. Molecular analysis showed increased expression of MuRF1, markers for oxidative stress (Nox2, UCP3, ubiquitin, and nitrotyrosine) and autophagy (LC3). In addition, actin content was decreased. These molecular effects were reversed by MCEMBL#205.

**Conclusions:** Tumour development is associated with functional impairment and muscle atrophy related to increased ROS and autophagy. MCEMBL#205 might be a future pharmacological treatment to prevent functional decline in tumour cachexia patients.

**3-15**

**The effects of a combination of bisoprolol and erythropoietin in experimental cancer cachexia**

Sandra Palus^1^, Kai Hartmann^1^, Tanja Braun^1^, Stephan von Haehling^2^, Wolfram Doehner^1^, Stefan D. Anker^1^ and **Jochen Springer** ^1^

^1^ *Division of Cardiology and Metabolism, Department of Cardiology (CVK); and Berlin Institute of Health Center for Regenerative Therapies (BCRT) Charité Universitätsmedizin Berlin, Germany;* ^2^ *Department of Cardiology and Pneumology, University Medical Centre Göttingen, Göttingen, Germany*

We have previously shown that 5 mg/kg/g bisoprolol and 100 U/kg/day erythropoietin (EPO) significantly improve outcome in experimental cancer cachexia. In this study, we investigated the influence of a 25% or 75% combination of both compounds versus effective doses of bisoprolol, EPO, or placebo in the Yoshida hepatoma cancer cachexia model. Bisoprolol (HR: 0.37; 95% CI: 0.21–0.68, *P* = 0.0033), EPO (HR: 0.47; 95% CI: 0.27–0.81, *P* = 0.0112), the 25% combination (HR: 0.54; 95% CI: 0.31–0.98, *P* = 0.0515), and the 75% combination (HR: 0.41; 95% CI: 0.23–0.74, *P* = 0.0076) improved survival compared to placebo. The 25% and 75% combinations reached HR: 1.49; 95% CI: 0.58–3.85, *P* = 0.34 and HR: 1.22; 95% CI: 0.45–3.33, *P* = 0.67) versus 5 mg/kg/day bisoprolol, respectively, and versus 100 U/kg/day EPO HR: 1.13; 95% CI: 0.46–2.79, *P* = 0.77 and HR: 0.90; 95% CI: 0.34–2.63 *P* = 0.83, respectively. High dose combination therapy shows significant attenuation of fat, lean, and weight loss, which is reflected in the tissue weights.

**Table:** 

	Placebo	5 mg/kg/day bisoprolol	100 U/kg/day EPO	25% combination biso + EPO	75% combination biso + oxy
*n*	78	23	20	15	15
Δ Body weight, g	−53.7 ± 1.8	−21.9 ± 10.5^***^	−30.6 ± 9.6^***^	−48.1 ± 9.4	−22.7 ± 11.7^***^
Δ Fat mass, g	−12.4 ± 0.4	−5.9 ± 1.9^***^	−8.0 ± 1.7^***^	−11.0 ± 2.2	−7.1 ± 2.5^***^
Δ Lean mass, g	−39.8 ± 1.6	−16.7 ± 7.7^***^	−23.7 ± 7.4^**^	−37.3 ± 6.8	−18.0 ± 9.2^***^
Gastrocnemius, mg	729 ± 12	840 ± 66^***^	854 ± 42^***^	808 ± 40^*^	937 ± 59^***^
Soleus, mg	69.4 ± 0.9	74.1 ± 3.8	77.1 ± 2.9^**^	72.5 ± 2.9	81.4 ± 3.8^***^
EDL, mg	64.5 ± 1.1	72.8 ± 4.1^**^	75.4 ± 3.0^***^	69.2 ± 3.6	81.1 ± 4.5^***^
WAT, mg	112 ± 19	519 ± 119^***^	329 ± 116^**^	201 ± 122	417 ± 128^***^
BAT, mg	87.6 ± 2.9	119.4 ± 12.2^**^	92.3 ± 9.6	106.5 ± 16.7	180.7 ± 48.7^***#^
Food intake day, 11 g/24 h	4.3 ± 0.5	10.9 ± 2.0^***^	7.9 ± 1.7^**^	13.3 ± 6.4^**^	10.6 ± 2.0^**^
Activity day, 11 counts/24 h	29 509 ± 1775	43 755 ± 3741^***^	38 550 ± 4412^**^	40 235 ± 6336^**^	46 406 ± 6293^***^

^*^ *P* < 0.05.

^**^ *P* < 0.01.

^***^ *P* < 0.001 versus placebo.

^#^ *P* < 0.05 versus 100 U/kg/day EPO.

Taken together, these results suggest that a combination of bisoprolol and EPO allows the reduction of the respective dose to 75% of mono‐therapy in experimental cancer cachexia.

**3-16**

**The effects of a combination of bisoprolol and oxypurinol in experimental cancer cachexia**

Sandra Palus^1^, Kai Hartmann^1^, Tanja Braun^1^, Stephan von Haehling^2^, Wolfram Doehner^1^, Stefan D. Anker^1^ and **Jochen Springer** ^1^

^1^ *Division of Cardiology and Metabolism, Department of Cardiology (CVK); and Berlin Institute of Health Center for Regenerative Therapies (BCRT) Charité Universitätsmedizin Berlin, Germany;* ^2^ *Department of Cardiology and Pneumology, University Medical Centre Göttingen, Göttingen, Germany*

We have previously shown that 5 mg/kg/g bisoprolol and 4 mg/kg/day oxypurinol significantly improve outcome in experimental cancer cachexia. In this study, we investigated the influence of a 25% or 75% combination of both compounds versus effective doses of bisoprolol, oxpurinol, or placebo in the Yoshida hepatoma cancer cachexia model. Bisoprolol (HR: 0.37; 95% CI: 0.21–0.68, *P* = 0.0033), oxypurinol (HR: 0.21; 95% CI: 0.11–0.40, *P* = 0.0008), the 25% combination (HR: 0.53; 95% CI: 0.24–1.17, *P* = 0.15), and the 75% combination (HR: 0.24; 95% CI: 0.12–0.47, *P* = 0.0025) improved survival compared to placebo. The 25% and 75% combinations reached HR: 1.84; 95% CI: 0.47–7.25, *P* = 0.27 and HR: 0.77; 95% CI: 0.22–2.63, *P* = 0.67) versus 5 mg/kg/day bisoprolol, respectively, and versus 4 mg/kg/day oxypurinol HR: 2.30; 95% CI: 0.49–10.69, *P* = 0.23 and HR: 1.11; 95% CI: 0.22–5.52, *P* = 0.89, respectively. The combination therapy shows significant attenuation of fat, lean, and weight loss, but less pronounced than the respective mono‐therapy.

**Table:** 

	Placebo	5 mg/kg/day bisoprolol	4 mg/kg/day oxypurinol	25% combination biso + oxy	75% combination biso + oxy
*n*	78	23	11	8	11
Δ Body weight, g	−53.7 ± 1.8	−21.9 ± 10.5^***^	−15.4 ± 14.3^***^	−40.4 ± 15.7	−38.2 ± 11.1^*^
Δ Fat mass, g	−12.4 ± 0.4	−5.9 ± 1.9^***^	−6.8 ± 2.6^***^	−9.0 ± 2.8^*^	−9.9 ± 2.2
Δ Lean mass, g	−39.8 ± 1.6	−16.7 ± 7.7^***^	−10.6 ± 10.4^***^	−33.0 ± 12.6	−28.5 ± 8.3^*^
Gastrocnemius, mg	729 ± 12	840 ± 66^***^	907 ± 79^***^	818 ± 82	881 ± 50^***^
Soleus, mg	69.4 ± 0.9	74.1 ± 3.8	80.6 ± 5.6^**^	63.7 ± 4.8	79.3 ± 4.1^**^
EDL, mg	64.5 ± 1.1	72.8 ± 4.1^**^	85.3 ± 6.8^***^	70.6 ± 6.5	76.4 ± 4.4^**^
WAT, mg	112 ± 19	519 ± 119^***^	506 ± 156^***^	217 ± 164	312 ± 156^*^
BAT, mg	87.6 ± 2.9	119.4 ± 12.2^**^	123.6 ± 17.2^***^	115.8 ± 25.5^*^	92.5 ± 14.1
Food intake day, 11 g/24 h	4.3 ± 0.5	10.9 ± 2.0^***^	13.0 ± 2.9^***^	5.5 ± 2.6	7.2 ± 2.3
Activity day, 11 counts/24 h	29 509 ± 1775	43 755 ± 3741^***^	48 343 ± 7724^***^	30 327 ± 6732	36 772 ± 6437

^*^ *P* < 0.05.

^**^ *P* < 0.01.

^***^ *P* < 0.001 versus placebo.

Taken together, these results suggest that a combination of bisoprolol and oxypurinol allows the reduction of the respective dose to 75% of mono‐therapy in experimental cancer cachexia.

**3-17**

**The combination of bisorpolol and the tissue protective molecule ARA 284 shows synergistic effects**

Yulia Elkina^1^, Sandra Palus^1^, Tanja Braun^1^, Stephan von Haehling^2^, Wolfram Doehner^1^, Stefan D. Anker^1^, Anthony Cerami^3^, Michael Brines^3^ and **Jochen Springer** ^1^

^1^ *Division of Cardiology and Metabolism, Department of Cardiology (CVK); and Berlin Institute of Health Center for Regenerative Therapies (BCRT) Charité Universitätsmedizin Berlin, Germany;* ^2^ *Department of Cardiology and Pneumology, University Medical Centre Göttingen, Göttingen, Germany;* ^3^ *ARAIM Pharmaceuticals, Tarrytown, New York, USA*

We have previously shown that 5 mg/kg/g bisoprolol and 1.7 μg/kg/day ARA 284 significantly improve outcome in experimental cancer cachexia. In this study, we investigated the influence of a 25% or 75% combination of both compounds versus effective doses of bisoprolol, ARA 284, or placebo in the Yoshida hepatoma cancer cachexia model. Bisoprolol (HR: 0.37; 95% CI: 0.21–0.68, *P* = 0.0033), ARA 284 (HR: 0.54; 95% CI: 0.31–0.92, *P* = 0.0255), the 25% combination (HR: 0.19; 95% CI: 0.10–0.35, *P* = 0.003), and the 75% combination (HR: 0.08; 95% CI: 0.04–0.17, *P* = 0.0002) improved survival compared to placebo. The 25% and 75% combinations reached HR: 0.52; 95% CI: 0.16–1.66, *P* = 0.29 and HR: 0.24; 95% CI: 0.06–0.93, *P* = 0.12) versus 5 mg/kg/day bisoprolol, respectively, and versus 1.7 μg/kg/day ARA284 HR: 0.34; 95% CI: 0.12–0.98, *P* = 0.073 and HR: 0.15; 95% CI: 0.05–0.49, *P* = 0.033, respectively. Loss of fat and lean mass were attenuated leading to a reduced loss in body weight, which is reflected in tissue weights (see table).

**Table:** 

	Placebo	5 mg/kg/day bisoprolol	1.7 μg/kg/day ARA 284	25% combination Biso + ARA	75% combination Biso + ARA
*n*	78	23	22	12	9
Δ Body weight, g	−53.7 ± 1.8	−21.9 ± 10.5^***^	−24.9 ± 9.6^***^	−39.3 ± 14.8	−14.78 ± 18.1^***^
Δ Fat mass, g	−12.4 ± 0.4	−5.9 ± 1.9^***^	−7.6 ± 1.7^***^	−12.5 ± 2.1	−7.35 ± 3.1^**^
Δ Lean mass, g	−39.8 ± 1.6	−16.7 ± 7.7^***^	−18.6 ± 7.5^***^	−35.6 ± 10.6	−11.5 ± 14.1^***^
Gastrocnemius, mg	729 ± 12	840 ± 66^***^	833 ± 49^***^	841 ± 66^**^	1001 ± 89^***#^
Soleus, mg	69.4 ± 0.9	74.1 ± 3.8	74.0 ± 3.1	75.2 ± 3.9^*^	83.0 ± 5.4^***^
EDL, mg	64.5 ± 1.1	72.8 ± 4.1^**^	67.9 ± 3.9	74.9 ± 5.4^**^	83.0 ± 7.6^***#^
WAT, mg	112 ± 19	519 ± 119^***^	487 ± 121^***^	323 ± 128^**^	449 ± 181^***^
BAT, mg	87.6 ± 2.9	119.4 ± 12.2^**^	97.8 ± 10.1	113.5 ± 18.8^*^	135.5 ± 22.1^***#^
Food intake day, 11 g/24 h	4.3 ± 0.5	10.9 ± 2.0^***^	9.9 ± 2.8^**^	7.83 ± 2.33^*^	13.6 ± 2.9^***^
Activity day, 11 counts/24 h	29 509 ± 1775	43 755 ± 3741^***^	41 145 ± 5422^**^	35 233 ± 5767	47 386 ± 6537^**^

^*^ *P* < 0.05.

^**^ *P* < 0.01.

^***^ *P* < 0.001 versus placebo.

^#^ *P* < 0.05 versus ARA284.

Taken together, these results suggest that a combination of bisoprolol and ARA 284 shows synergistic effects in the treatment of experimental cancer cachexia.

**3-18**

**Targeting angiotensin‐II signalling in experimental cancer cachexia**

Sandra Palus^1^, Cathleen Drescher^2^, Wolfram Döhner^1^, Stephan von Haehling^3^, Stefan D. Anker^1^ and **Jochen Springer** ^1^

^1^ *Division of Cardiology and Metabolism, Department of Cardiology (CVK); and Berlin Institute of Health Center for Regenerative Therapies (BCRT) Charité Universitätsmedizin Berlin, Germany;* ^2^ *German Institute of Human Nutrition, Potsdam, Germany;* ^3^ *Department of Cardiology and Pneumology, University Medical Centre Göttingen (UMG), Göttingen, Germany*

**Background:** Angiotensin‐II has been shown to be up‐regulated in catechetic states. Angiotensin‐II predominantly elicits AT1 mediated responses, because of the predominant expression of the AT1 receptor. However, the ACE‐inhibitor imidapril had no effect on survival in the Yoshida hepatoma model (Springer *et al*. *Eur Heart J*. 2013). Here, we tested the AT1 receptor antagonists olmesartan and telmisartan and the AT2 agonist coumpound‐21 in the Yoshida hepatoma cancer cachexia model.

**Results:** The effects of oral treatment olmesartan and telmisartan (both at 1 or 5 mg/kg/day) and compound‐21 (C‐21 at 0.2 or 1 mg/kg/day) on survival, body weight, and body composition were tested in approximately 200 g male Wistar rats versus placebo. Both sartans had no significant beneficial effect on survival: 1 mg/kg/day olmesartan (*n* = 12; HR: 1.98; 95% CI: 0.82–4.79; *P* = 0.13), 5 mg/kg/day olmesartan (*n* = 16; HR: 2.34; 95% CI: 1.06–5.18; *P* = 0.0357), 1 mg/kg/day telmisartan (*n* = 114; HR: 0.62; 95% CI: 0.30–1.29; *P* = 0.20), and 5 mg/kg/day telmisartan (*n* = 14; HR: 4.76; 95% CI: 1.85–12.3; *P* = 0.0012); all versus placebo (*n* = 44). C‐21 was effective in the lower dose (HR: 0.45; 95% CI: 0.22–0.92, *P* = 0.0275, *n* = 15), but not in the high dose (HR: 2.08; 95% CI: 0.85–5.11, *P* = 0.014, *n* = 10). Rats showed no difference in baseline body weight. While weight loss was attenuated by olmesartan (1 mg/kg/day: −32 ± 5 g; 5 mg/kg/day: −30 ± 6 g vs. placebo: −51 ± 2 g, both *P* < 0.05), C‐21 at 0.2 mg/kg/day significantly reduced weight loss (−28 ± 10 g; *P* = 0.003) while 1 mg/kg/day had no effect (−55.2 ± 4.3 g). Telmisartan had no effect on weight loss. Olmesartan (1 mg/kg/day: −17.3 ± 4.4 g; 5 mg/kg/day: −9.2 ± 2.9 g) and 5 mg/kg/day telmisartan (−26.4 ± 8.0 g) reduced wasting of lean body mass compared to placebo (−33.1 ± 1.9 g, all *P* < 0.05). Low dose C‐21 reduced lean mass wasting (−28.7 ± 4.9 g). Placebo‐treated rats lost 12.7 ± 0.5 g fat mass, which was significantly reduced by 1 mg/kg/day (−7.3 ± 1 g, *P* < 0.001) or 5 mg/kg/day olmesartan (−7.1 ± 0.8 g, *P* < 0.001), while telmisartan had no effect on fat mass wasting. C‐21 reduced fat loss somewhat (−10.8 ± 1.1 g).

**Conclusions:** Taken together, olmesartan and telmisartan had very limited effects in the Yoshida hepatoma model, and the reduction of wasting may be due to an earlier death of the animals. Treatment with low dose C‐21 improved survival and reduced wasting.

**3-19**

**The erythropoietin‐derived peptide ARA 284 reduces tissue wasting and improves survival in a rat model of cancer cachexia**

Yulia Elkina^1^, Sandra Palus^1^, Tanja Braun^1^, Stephan von Haehling^2^, Wolfram Doehner^1^, Stefan D. Anker^2^, Anthony Cerami^3^, Michael Brines^3^ and **Jochen Springer** ^1^

^1^ *Division of Cardiology and Metabolism, Department of Cardiology (CVK); and Berlin Institute of Health Center for Regenerative Therapies (BCRT) Charité Universitätsmedizin Berlin, Germany;* ^2^ *Department of Cardiology and Pneumology, University Medical Centre Göttingen, Göttingen, Germany;* ^3^ *ARAIM Pharmaceuticals, Tarrytown, New York, USA*

Cancer cachexia is a severe complication during the last stages of the disease, which is characterized by the substantial loss of muscle and fat mass. At the present time, there is no effective treatment of cancer cachexia. Erythropoietin is a hormone that plays tissue protective role in different tissues. Based on the structure of erythropoietin, small peptides have been synthesized which are not erythropoietic but activate the tissue protective signalling pathways. In this study, we investigated the influence of the tissue protective peptide ARA 284 on cancer cachexia in rats. We show that administration of this compound (1.7 μg/kg/day) counteracted the loss of total body weight (12.46 ± 4.82% ARA 284 vs. 26.85 ± 0.88% placebo, *P* = 0.0058), fat mass (*P* = 0.027), and lean mass (*P* = 0.012), as well as significantly improved the spontaneous activity of the ARA 284 treated animals. Further, gastrocnemius muscle mass was shown to be increased (13.2% ARA 284 vs. placebo, *P* = 0.015) in association with a higher level of activated Akt (*P* = 0.0038), and a decrease in activated p38 MAPK, GSK‐3β, and myostatin (all *P* < 0.0001). These results suggest that an induction of anabolic pathways underlies the increase of muscle tissue. At the same time, we observed the significant increase in the survival of animals (*P* < 0.05). Taken together, these results suggest that ARA 284 can be considered as a prospective drug to improve conditions of patients with cancer cachexia.

**4-01**

**CASC‐IN: a new tool to diagnose pre‐cachexia in cancer patients**

**Sílvia Busquets** ^1,2^, Francisco J. López‐Soriano^1,2^, Marta Castillejo, Cristina Moreno and Clelia Madeddu^3^ and Roberto Serpe^3^ and Josep M. Argilés^1,2^

^1^ *Cancer Research Group, Department of Biochemistry and Molecular Biology, Faculty of Biology, University of Barcelona, Barcelona, Spain;* ^2^ *Institute of Biomedicine (IBUB), Barcelona, Spain;* ^3^ *Department of Medical Oncology, University of Cagliari, Cagliari, Italy*

**Introduction:** Diagnose is particularly important in those patients that are not yet cachectic but suffer from pre‐cachexia, a potential early stage of cachexia. Pre‐cachectic patients should be screened particularly since this group may be the target of multimodal intervention trials; therefore, a clear, easy to use, diagnostic tool is clearly needed. The aim of the present investigation has been to develop an easy‐to‐use tool (CASC‐IN) for the evaluation of pre‐cachectic cancer patients.

**Methods**: The CASC‐IN score has been designed with two very clear objectives: first, as a means to quantitatively assess pre‐cachexia in cancer patients, and second, as a tool to discriminate the patients where cachexia staging is relevant. From this point of view, the CASC‐IN tool is tightly linked with the so‐called CAchexia SCOre (CASCO) previously described [1] and validated [2] by our research team.

**Results:** The results presented here classify a population of cancer patients into non‐cachectic, pre‐cachectic or cachectic. Using a population of 179 cancer patients, affected by a different tumour types, the frequencies observed have been: non‐cachectic: 58 (32.4%), pre‐cachectic: 7 (3.9%), and cachectic: 114 (63.7%).

**Conclusions:** CASC‐IN not only permits the identification of those patients that are pre‐cachectic but also serves to discriminate those patients which are cachectic and, therefore, can be included in staging determination by means of either CASCO or MiniCASCO.

**References**

1. Argilés JM, López‐Soriano FJ, Toledo M, Betancourt A, Serpe R, Busquets S. The cachexia score (CASCO): a new tool for staging cachectic cancer patients. *J Cachexia Sarcopenia Muscle* 2011;**2**:87–93.

2. Argilés JM, Betancourt A, Guàrdia‐Olmos J, Peró‐Cebollero M, López‐Soriano FJ, Madeddu C *et al*. Validation of the CAchexia SCOre (CASCO). *Staging cancer patients: the use of miniCASCO as a simplified tool. Front Physiol* 2017;**8**:92.

**4-02**

**A new diagnostic biomarker for cachexia: the role of serum creatinine/cystatin C ratio**

**Takayuki Tsuneda** ^1^, Masanobu Takata^2^, Hidehiko Nagasawa^1^, Atsuhiro Shimakura^1^, Yoshinobu Hinoue^1^ and Takeshi Yamashita^3^

^1^ *Department of Cardiology, Toyama Machinaka Hospital, Toyama, Japan;* ^2^ *Department of Internal Medicine, Toyama Nishi General Hospital, Toyama, Japan;* ^3^ *Department of Cardiovascular Medicine, The Cardiovascular Institute, Tokyo, Japan*

**Introduction:** Cachexia is known as a complex metabolic syndrome with chronic illness. It is characterized by accelerated loss of skeletal muscle and losing body weight. Recent reports have shown that low serum creatinine/cystatin C (SCr/CysC) ratio indicated muscle wasting with physical disability. Therefore, we evaluated retrospectively the role of SCr/CysC ratio as a biomarker of cachexia, defined by Evans' criteria.

**Methods:** We enrolled 444 patients with chronic illness in our hospital (81 ± 12 years old; men, 44%), and collected SCr, CysC, blood urea nitrogen, albumin, haemoglobin, and C‐reactive protein. Cachexia was defined by Evans' criteria in 2008. For assessment of muscle wasting, we quantified the area of bilateral psoas muscle normalized by height (Total Psoas Index: TPI, mm^2^/m^2^), using computed tomography. The cut‐off values of TPI in Asian adults were used as 636 and 392 mm^2^/m^2^ in male and female, respectively. Decreased muscle strength was translated by grade 3 and 4 in ECOG‐performance status.

**Results:** Ninety‐four patients were diagnosed as cachexia and showed lower SCr/CysC ratio than non‐cachectic patients (0.054 ± 0.014 vs. 0.074 ± 0.021, respectively). The lower SCr/CysC ratio predicted cachexia with 84% (69–93%) sensitivity and 72% (64–78%) specificity in male and with 68% (55–80%) sensitivity and 71% (64–77%) specificity in female [area under the curve: 0.83 (0.76–0.89) and 0.75 (0.68–0.82)], using receiver operating characteristic analysis. Cut‐off values of SCr/CysC ratio were 0.071 in male and 0.058 in female with cachexia. According to multivariate logistic regression analysis, predictors for cachexia were anorexia, fatigue, and lower SCr/CysC ratio [odds ratios: 19.8 (8.3–43.4), 2.7 (1.4–5.2), and 5.1 (2.6–10.2), respectively].

**Conclusions:** Our data indicated that lower SCr/CysC ratio, in addition with cachectic symptoms, was closely related to cachexia with chronic illness in Japanese patients. Therefore, SCr/CysC ratio might be one of the most efficient diagnostic biomarker for cachexia.

**4-03**

**Cachexia in individuals with head and neck squamous cell carcinoma**

Luciana Mara Pereira^1^, Lucas Augusto Pereira Souto^1^, Magda Mendes Vieira^1^, João Lucas Rodrigues dos Santos^1^, Amanda Mota Lacerda^1^, Bruna Nathália Santos^1^, Walter de Freitas Filho^1^, Lorrane Katherine Martins Pereira^1^, Gefter Thiago Batista Corrêa^2^ and **Alfredo Mauricio Batista De‐Paula** ^1,3^

^1^ *Nucleus of Epidemiologic and Molecular Research Catrumano (Nupemoc), Health Research Laboratory, Post‐graduate Program in Health Sciences, Universidade Estadual de Montes Claros, UNIMONTES, Montes Claros, Minas Geras, Brazil;* ^2^ *Department of Dentistry, Faculdade Independente do Nordeste, FAINOR, Vitória da Conquista, Bahia, Brazil;* ^3^ *Department of Dentistry, Universidade Estadual de Montes Claros, Unimontes, Montes Claros, Minas Gerais, Brazil*

**Introduction:** Head and neck squamous cell carcinoma (HNSCC) is the most prevalent cancer that affects the upper aerodigestive tract mucosa. During HNSCC progression, individual frequently exhibits anorexia, dysphagia, an increased catabolic activity in skeletal muscle and white adipose tissues, and a low‐grade, chronic systemic inflammatory state that characterize the paraneoplastic syndrome known as cancer related‐cachexia (CRC). The present study aims to investigate the association between CRC stages (pre‐cachectic and cachectic) in individuals with HNSCC and a number of clinical, anthropometrical, plasma biochemical parameters.

**Methods:** This study evaluated 70 individuals with HNSCC (male : female ratio: 3/1; age: 60.5 ± 8.9) and controls (male: female ratio: 3/1; age: 59.6 ± 8.77). Individuals with HNSCC were evaluated for unintentional body weight loss over a period of 6 months and were categorized as non‐cachectic, pre‐cachectic, and cachectic (Fearon *et al*., 2011). The anthropometric and nutritional assessed were arm circumference (AC), triceps skinfold thickness (TCT), and body mass index (BMI). Serum high‐sensitive C‐reactive protein (CRP) and albumin levels were used to calculate modified Glasgow (mGPS) and high‐sensitivity mGPS (HS‐mGPS) prognostic scores. Moreover, individuals were submitted to the assessment of the muscle strength using a handgrip dynamometer. This study was approved by a human research ethics committee (CONEPE No. 19462/2019).

**Results:** Individuals with HNSCC in cachectic stage significantly exhibited higher unintentional body weight loss and dysphagia. However, individuals with CRC exhibited lower AC, TCT, BMI, handgrip strength, and concentration of plasmatic albumin. Individuals with HNSCC in cachectic stage showed higher CRP plasma levels and HS‐mGPS scores.

**Conclusions:** CRC in individuals with HNSCC is associated with occurrence of low‐grade, chronic inflammatory state, and a higher declining physical functioning. Early diagnosis of CRC in individuals with HNSCC might be encouraged in order to obtain a better prognosis.

**4-04**

**Systemic inflammation markers resulting in cardiovascular risk**

Estefanía Simoes^1^, Joanna Darck Carola Correia Lima^1^, Gabriela Salim de Castro^1^, Silvio Pires Gomes^1^, Fang Chia Bin^4^, José Pinhata Otoch^1,2,3^, Paulo Sergio Martins de Alcantara^2^, Alessandro Laviano^5^ and **Marilia Seelaender** ^1,3^

^1^ *Cancer Metabolism Research Group, University of São Paulo, Brazil;* ^2^ *Department of Clinical Surgery, University Hospital, University of São Paulo, Brazil;* ^3^ *Faculdade de Medicina, University of São Paulo, Brazil;* ^4^ *Santa Casa de Misericórdia de São Paulo;* ^5^ *Department of Clinical Medicine, Sapienza University of Rome, Italy*

**Background:** Cancer cachexia is a devastating syndrome that involves systemic inflammation with multiorgan consequences. The syndrome leads to exacerbated muscle protein degradation, resulting in an increased level of circulating amino acids that sustain the acute‐phase response proteins (APRPs) synthesis in the liver. Constant production of APRPs causes hypermetabolism, angiogenesis, and reduced energy intake. APRPs, such as C‐reactive protein (CRP) and albumin, have been used as cachexia prognosticators. Discovery of new consistent markers may benefit cachexia diagnosis. Moreover, the interaction between APRPs with intravascular content may explain other cachexia related symptoms. To elucidate new APRPs for cachexia diagnosis and possible correlations of APRPs with the biochemical status of cachectic patients and cardiovascular risk.

**Methods:** Patients with colorectal cancer were divided into Weight‐Stable Cancer (WSC, *n* = 17) and Cachectic Cancer (CC, *n* = 11) groups. Blood sampling was performed after signature of the informed consent form. Protein content was quantified with Luminex®xMAP.

**Results:** CC patient presented higher levels of CRP (*P* = 0.0004) and lower levels of haemoglobin (*P* = 0.0003) and albumin (*P* = 0.0011). Moreover, lower levels of ADAMTS13 were detected in the serum samples of cachectic patients relative to WSC (*P* = 0.0026). D‐Dimer (*P* = 0.034), NGAL (*P* = 0.043), and SAA (*P* = 0.0139) were significantly increased in CC patients. No differences were observed when we evaluated myoglobin, MPO, P‐selectin, and sVCAM. Also, CC presented correlations between Hb and CRP (*P* = 0.019, *r* = 0.721); CRP and SAA (*P* = 0.049, *r* = 0.603); albumin and ADAMTS13 (*P* = 0.0023, *r* = 0.815); albumin and weight loss (*P* = 0.029, *r* = −0.65); NGAL and weight loss (*P* = 0.003, *r* = 0.80). No significant correlations were found for WSC.

**Conclusions:** Low levels of ADAMTS13 and high levels of D‐Dimer and NGAL are evidences of intravascular risk and are associated to increased risk of thrombotic disease, weight loss, and systemic inflammation. Moreover, acute‐phase response proteins, such as ADAMTS13, D‐Dimer, NGAL, and SAA could be considered new markers of systemic inflammation and cachexia diagnosis.

**4-05**

**Dysregulation of small non‐coding RNAs in the skeletal muscle and in plasma‐derived microvesicles during experimental cancer cachexia**

**Marc Beltrà** ^1,2^, Lorena García‐Castillo^1,2^, Fabrizio Pin^1,2^, Serena De Lucia^1,2^, Riccardo Ballarò^2^, Giovanni Birolo^3,4^, Barbara Pardini^3,4^, Giuseppe Matullo^3,4^, Fabio Penna^1,2^ and Paola Costelli^1,2^

^1^ *Department of Clinical and Biological Sciences, Experimental Medicine and Clinical Pathology Unit, University of Turin, Italy;* ^2^ *Interuniversity Institute of Myology (IIM), Urbino, Italy;* ^3^ *Italian Institute for Genomic Medicine, IIGM (formerly Human Genetics Foundation, HuGeF), Turin, Italy;* ^4^ *Department of Medical Sciences, University of Turin, Turin, Italy*

**Introduction:** MicroRNAs (miRNAs) are endogenous short RNA molecules that modulate the expression of specific genes by inhibiting their translation, thus exerting essential regulatory functions. In this regard, increased muscle protein catabolism, including that occurring in cancer cachexia, has been shown to correlate with miRNAome alterations (1, 2). Furthermore, miRNAs can be secreted in the blood, free or clotted in extracellular vesicles (microvesicles and exosomes), and distributed throughout the whole organism. Evidences demonstrate that these miRNA‐containing vesicles can be up‐taken by cells and contribute to signalling (3). Similar to muscle, circulating miRNAs are found modulated in many pathologies, therefore being good potential candidates for diagnosis and treatment.

**Methods:** In the attempt to characterize the miRNA pattern in a setting of cancer cachexia, we isolated total RNA from *gastrocnemius* muscle and plasma‐derived microvesicles of both healthy and cachectic mice bearing the transplantable C26 colon carcinoma. As for plasma, samples of two mice were pooled, and consecutive centrifugations were applied for microvesicle isolation. Next‐generation sequencing (Illumina) was used to sequence whole miRNA transcriptome. We also extended the alignment analysis of the remaining unmapped sequences to other candidate small non‐coding RNAs.

**Results:** About 30 out of the 304 detected miRNAs were differentially regulated in the skeletal muscle of C26‐bearing mice. By contrast, miRNAs extracted from plasma‐derived microvesicles appear poorly modulated in the C26 hosts; only miR‐181a‐5p, miR‐375‐3p, and miR‐455‐5p were found dysregulated from a total of 118. Finally, the analysis of the remaining unmapped sequences revealed that muscle from mice bearing the C26 had eight up‐regulated snoRNAs, while a general down‐regulation of mt‐tRNAs was observed (12 out of the 21 detected).

**Conclusions:** These results bring new insight into the modulation of muscle and circulating miRNA expression during cancer cachexia, as well as new data concerning the involvement of other types of small non‐coding RNAs.

**References**

1. Narasimhan A et al., J Cachexia Sarcopenia Muscle., 2017

2. Soares RJ et al., J Biol Chem., 2014

3. He WA et al., Proc Natl Acad Sci U S A., 2014

**4-06**

**Automated CT‐derived skeletal muscle mass determination in mice using a 3D U‐Net deep learning network**

**Wouter R.P.H. van de Worp** ^†, 1^, Brent van der Heyden^†, 2^, Ardy van Helvoort^1,3^, Jan Theys^4^, Annemie M.W.J. Schols^1^, Ramon C.J. Langen^1^ and Frank Verhaegen^2^

^1^ *Department of Respiratory Medicine, NUTRIM—School of Nutrition and Translational Research in Metabolism, Maastricht University Medical Center, Maastricht, The Netherlands;* ^2^ *Department of Radiation Oncology (MAASTRO), GROW—School for Oncology and Developmental Biology, Maastricht University Medical Centre, Maastricht, The Netherlands;* ^3^ *Danone Nutricia Research, Nutricia Advanced Medical Nutrition, Utrecht, The Netherlands;* ^4^ *Department of Precision Medicine, GROW—School for Oncology and Developmental Biology, Maastricht University Medical Center, Maastricht, The Netherlands*

^†^Both authors contributed equally.

**Introduction:** The loss of skeletal muscle mass is recognized as a complication of several chronic diseases and is associated with increased mortality and a decreased quality of life. Relevant and reliable animal models in which muscle wasting can be monitored longitudinally are instrumental to investigate and develop new therapies. However, the non‐invasive follow‐up of time‐dependent changes in muscle mass is time consuming and challenging due to technical requirements. In this work, we developed a fully automatic deep learning algorithm for segmentation of micro cone beam CT (μCBCT) images of the lower limb muscle complex in mice and subsequent muscle mass calculation.

**Methods:** Whole body μCBCT images were obtained from anaesthetized mice using a X‐RAD 225Cx image‐guided biological irradiator system, and the lower hind leg musculature was manually segmented using SmART‐ATP software. A deep learning algorithm was constructed (two‐step 3D U‐Net model) and trained on manually segmented data from 32 mice. Muscle wet mass measurements were obtained of 47 mice and served as a dataset for model validation and the reverse model validation.

**Results:** The automatic algorithm performance was approximately 150 times faster than manual segmentation. Reverse validation of the algorithm showed high quantitative metrics, i.e. a Dice Similarity Coefficient (DSC) of 0.93 and a submillimeter precision in Hausdorff distance and center of mass displacement, substantiating the robustness and accuracy of the model. A high correlation (*R* ^2^ = 0.92) was obtained between the CT‐derived muscle mass measurements and the muscle wet masses. Longitudinal follow‐up revealed time‐dependent changes in muscle mass that separated control from lung tumour bearing mice, which was confirmed as cachexia.

**Conclusions:** This deep learning model for automated assessment of the lower limb muscle complex provides highly accurate non‐invasive longitudinal evaluation of skeletal muscle mass. Furthermore, it facilitates the workflow and increases the amount of data derived from mouse studies, while reducing the animal numbers.

**5-01**

**Longitudinal characterization of muscle alterations in a rodent model of NAFLD**

**Héloïse Louvegny** ^1^, Maxime Nachit^1,2^, Caroline Bouzin^3^, Jean‐Paul Thissen^4^, Yves Horsmans^5^, Greetje Vande Velde^2^ and Isabelle A. Leclercq^1^

^1^ *Laboratory of Hepato‐Gastroenterology, Institut De Recherche Expérimentale Et Clinique, Université Catholique De Louvain;* ^2^ *Department of Imaging & Pathology, KU, Leuven;* ^3^ *IREC Imaging Platform, Université Catholique De Louvain;* ^4^ *Endocrinology, Diabetology, and Nutrition Department, Institut De Recherche Expérimentale Et Clinique, Université Catholique De Louvain;* ^5^ *Service D'hépato‐Gastro‐Entérologie, Cliniques Universitaires Saint‐Luc, Brussels*

**Background:** Non‐alcoholic fatty liver disease (NAFLD) is the most common chronic liver disease. A substantial body of literature supports that a low muscle mass, low strength, or a higher muscle fatty infiltration are associated with NAFLD presence and severity. Here, the muscle compartment was evaluated longitudinally in a widely used NAFLD rodent model.

**Methods:** For 8 weeks, we followed C57BL6/J fed a high‐fat diet with choline deficiency (CDAA‐HFD) or supplemented in choline (CSAA‐HFD). Metabolic evaluation included oral glucose tolerance tests (OGTT), and fasting glycaemia were measured every 2 weeks. Dorsal muscles area and density (i.e. surrogates for muscle mass and myosteatosis) were assessed non‐invasively with micro‐computed tomography (micro‐CT) at weeks 0, 4, 6, and 8 (W0, 4, 6, 8). Muscle strength was evaluated every 2 weeks with a grip test. Quadriceps was harvested and weighted and liver examined by histology at W4 and W8.

**Results:** At W8, CSAA‐HFD developed glucose intolerance (Figure 1A) and mild liver steatosis with no inflammation or fibrosis. By contrast, CDAA‐HFD had normal fasting glycaemia (90.5 ± 9.8 mg/dL) and glucose tolerance (Figure 1A), but severe steatohepatitis (steatosis, severe inflammation) with pericellular fibrosis at W8. CDAA‐HFD had a significantly lower weight than CSAA‐HFD from W4 on (21.41 ± 1.36 g vs. 24.85 ± 1.27 g, *P* < 0.0001). While constant in CSAA‐HFD, liver density was strikingly lower in CDAA‐HFD from W4 on (0.91 ± 0.04 vs. −0.18 ± 0.12, *P* < 0.0001). Dorsal muscles area and quadriceps weight were lower in CDAA‐HFD than in CSAA‐HFD from W4 on (Figure 1B and 1C). Muscle density remained high in both groups, albeit significantly lower in CSAA‐HFD at W8 (0.80 ± 0.04 vs. 0.89 ± 0.05, *P* < 0.05). Muscle strength did not change over the study period in both groups.

**Figure 1:**
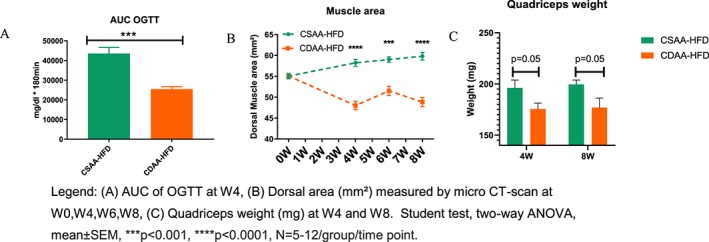
(A) AUC of OGTT at W4, (B) dorsal area (mm^2^) measured by micro CT‐scan at W0, W4, W6, and W8, (C) quadriceps weight (mg) at W4 and W8. Student's *t*‐test, two‐way ANOVA, mean ± SEM. ^***^ *P* < 0.001, ^****^ *P* < 0.0001, *N* = 5–12 per group per time point.

**Conclusions:** In CSAA‐HFD mice, mild myosteatosis develops together with mild liver steatosis and metabolic syndrome. In CDAA‐HFD, loss of muscle mass occurs together with severe steatohepatitis, but without myosteatosis or metabolic syndrome. Further studies are needed to decipher the respective contribution of dietary choline deficiency, metabolic alterations, and liver disease to changes in the muscle compartment.

**5-02**

**Micro‐CT imaging is an effective tool to detect muscle alterations non‐invasively**

**Maxime Nachit** ^1,2^, Maxime De Rudder^1^, Jean‐Paul Thissen^3^, Isabelle A. Leclercq^1^ and Greetje Vande Velde^2^

^1^ *Laboratory of Hepato‐Gastroenterology, Institut De Recherche Expérimentale Et Clinique, Université Catholique De Louvain, Belgium;* ^2^ *Department of Imaging & Pathology, KU, Leuven, Belgium;* ^3^ *Endocrinology, Diabetology, and Nutrition Department, Institut De Recherche Expérimentale Et Clinique, Université Catholique De Louvain, Belgium*

**Background:** Cross‐sectional clinical studies support that muscle alterations (i.e. low muscle mass, low strength, or a high muscle fatty infiltration) are associated to poor outcomes in almost all chronic diseases. To better understand how the muscle compartment influences disease progression, longitudinal studies in disease models are needed. To develop and validate a micro‐CT based methodology to measure muscle mass and fatty infiltration non‐invasively in small rodents.

**Methods:** Anaesthetized lean and obese mice, exhibiting a wide range of muscle alterations (i.e. low muscle mass and myosteatosis), were imaged using a Skyscan 1278 (Bruker, Belgium) at 50 μm voxel resolution. Whole body fat, lean (muscles and organs), and bone volumes were measured (Figure 1A) and transformed in grams to estimate total body weight. The surface (mm^2^) and density (in HU) of dorsal muscles (i.e. erector spinae/quadratus lumborum or E/Q, and psoas), were semi‐automatically measured at 4th and 5th lumbar levels (Figure 1B). A virtual liver biopsy was performed by placing a 3D cylindrical region of interest (±1.3 cm^3^) in the liver to measure the mean density (HU). Dorsal muscles and liver densities (Figure 1B) were respectively normalized to spleen density and expressed as a ratio. After scanning, mice were sacrificed, and muscles (i.e. E/Q, gastrocnemius, and quadriceps) and liver were harvested and weighted, and lipids were extracted for quantification. Correlations were tested with Pearson's coefficient.

**Results:** The procedure was feasible in 100% of tested mice, repeatable and well tolerated. Scanning time was 2.5 min. We found an excellent agreement between micro‐CT‐estimated and measured body weight (Figure 2A, *r* = 0.99, *n* = 69, *P* < 0.001). Dorsal muscle area was strongly correlated with both gastrocnemius (Figure 2B) and gastrocnemius + quadriceps weight (*r* = 0.85 and 0.83, respectively, *n* = 18, *P* < 0.001). Dorsal muscles density was strongly negatively correlated with dorsal muscles lipid content (Figure 2C, *r* = −0.83, *n* = 16, *P* < 0.0001). Liver density was strongly correlated with liver lipid content (*r* = −0.91, *n* = 69, *P* < 0.001).

**Conclusions:** Micro‐CT‐derived measurements accurately capture lean mass, muscle mass, and muscle and liver lipid content. This innocuous and high‐throughput methodology is readily applicable for longitudinal evaluation in pre‐clinical studies.

**5-03**

**Non‐invasive detection of myosteatosis predicts NASH in the context of metabolic syndrome and obesity**

**Maxime Nachit** ^1,2^, Greetje Vande Velde^2^, Wilhelmus J. Kwanten^3^, Olivier Schakman^4^, Jean‐Paul Thissen^5^, Maxime De Rudder^1^, Caroline Bouzin^6^, Bart Op De Beeck^7^, Yves Horsmans^8^, Luc Van Gaal^9^, Sven M. Francque^3,10^ and Isabelle A. Leclercq^1^

^1^ *Laboratory of Hepato‐Gastroenterology, Institut De Recherche Expérimentale Et Clinique, Université Catholique De Louvain;* ^2^ *Department of Imaging & Pathology, KU, Leuven;* ^3^ *Department of Gastroenterology and Hepatology, Antwerp University Hospital, Belgium;* ^4^ *Laboratory of Cell Physiology, Institute of Neuroscience, Université Catholique De Louvain;* ^5^ *Endocrinology, Diabetology, and Nutrition Department, Institut De Recherche Expérimentale Et Clinique, Université Catholique De Louvain;* ^6^ *IREC Imaging Platform, Université Catholique De Louvain;* ^7^ *Department of Radiology, Antwerp University Hospital, Antwerp, Belgium;* ^8^ *Service D'hépato‐Gastro‐Entérologie, Cliniques Universitaires Saint‐Luc, Brussels;* ^9^ *Laboratory of Experimental Medicine and Pediatrics, Division of Endocrinology, University of Antwerp, Belgium;* ^10^ *Laboratory of Experimental Medicine and Pediatrics, University of Antwerp, Belgium*

**Background:** A growing body of evidence links muscle alterations (i.e. loss of mass, strength, and myosteatosis) to the presence and severity of non‐alcoholic fatty liver disease (NAFLD). Here, we evaluated the muscle compartment with gold standard techniques, both longitudinally in a pre‐clinical NAFLD model and cross‐sectionally in a clinical cohort of morbidly obese patients.

**Methods:** For over 34 weeks (34W), we followed WT mice fed a standard chow as controls (Ctl), WT mice fed a high fat (HF) diet (60% fat) as a model of fatty liver (NAFL;WT HF) and foz/foz mice fed a HF diet as a model of progressive NASH (FOZ HF). We monitored muscle mass and density (i.e. myosteatosis) by micro‐CT and analysed muscles and liver histology at monthly intervals. We evaluated muscle mass and myosteatosis at fourth lumbar level with CT in a cohort of 185 morbidly obese patients with biopsy‐based liver histology (62.8%, 19.9%, and 17.3% had NASH, NAFL, and normal liver histology, respectively).

**Results:** Ctl had normal liver histology, WT HF developed obesity and isolated mild steatosis, and FOZ HF exhibited obesity and histologically proven NASH from 12W on. In FOZ HF at 34W, muscle size, strength, and density were significantly lower than in WT HF. Strikingly, muscle density was already significantly lower in FOZ HF after 4W of HF (0.79 ± 0.02) than in Ctl (0.91 ± 0.02) and further dropped in FOZ HF to reach a minimum at 12W (0.37 ± 0.05). Muscle density strongly negatively correlated with the NAFLD activity score (*r* = −0.87, *P* < 0.001, *n* = 67) and robustly discriminate NASH from NAFL (AUROC = 0.96, *P* < 0.0001, *n* = 67) (Figure 1A). In the cohort of 185 obese patients, muscle mass was unexpectedly significantly higher in patients with NASH than in those with NAFL or normal liver histology. By contrast, muscle density distinguishes NASH from NAFL with a high diagnostic power (Figure 1B, AUROC = 0.74, *P* < 0.0001) and independently from multiple confounders in these patients.

**Conclusions:** Myosteatosis strongly relates to NAFLD severity. We propose to further exploit this parameter to discern NASH from NAFL in NAFLD and monitor disease status.

**5-04**

**NMJ failure in aged C57BL/6J mice: defining the timeline and investigating potential therapeutics**

**W. David Arnold**, Deepti Chugh, Prameela Bobbili, Elizabeth Page, Michaiah Halley and Chitra Iyer

*The Ohio State University, Columbus, Ohio, USA*

**Background:** Sarcopenia is an important contributor to loss of physical function in older adults. Neurodegeneration has increasingly been recognized as a potential factor in the pathogenesis of sarcopenia. Prior *ex vivo* recordings of neuromuscular junction (NMJ) transmission have consistently demonstrated accentuated transmission rather than failure, but *ex vivo* recordings do not directly assess muscle fibre action potential generation. Single fibre electromyography (SFEMG) is a sensitive assessment of NMJ transmission routinely used in the clinic. Here, we investigated the following questions in aging C57BL/6J mice using the SFEMG technique: (i) Do aged mice show features of NMJ failure? (ii) At what ages does NMJ failure become evident? and (iii) Can therapeutic agents used in genetic and acquired NMJ disorders (myasthenic disorders) have a positive effect on NMJ transmission in aged mice?

**Methods:** Motor unit number estimation (MUNE), SFEMG, and contractility were obtained from the gastrocnemius muscle of mice aged 12, 20, 24, 27, and 29 months (*n* = 9–11 per age, balanced for sex). An additional cohort of aged mice (*n* = 7–8 per group) were treated with salbutamol 8 mg/kg daily or vehicle by intraperitoneal injection for 3 days and were compared using SFEMG, grip strength, and rotarod.

**Results:** MUNE and muscle contractility were significantly reduced at 20, 24, 27, and 29 compared to 12 months. In contrast, SFEMG jitter (variability of NMJ transmission) and blocking (failed NMJ transmission) were not significantly changed until 27 and 29 months. Salbutamol significantly improved SFEMG jitter (*P* < 0.0001) and blocking (*P* = 0.003), and rotarod (*P* = 0.046), but not grip (*P* = 0.074).

**Conclusions:** NMJ transmission failure occurred late in the lifespan of C57BL/6J mice. Salbutamol improved NMJ transmission and muscle performance in aged mice. Work is ongoing to investigate other potential therapeutics and exercise on NMJ failure. Future studies are needed to understand if similar NMJ defects are present in sarcopenic older adults.

**5-06**

**Anthropometric muscle mass measurements, mid‐arm muscle, and calf‐circumference as a mortality predictor in obese and non‐obese older adults: Cohort Elderly Project/Goiania**

**Erika Aparecida Silveira** ^1^, Cristina Camargo Pereira^1^, Annelisa Silva e Alves de Carvalho Santos^2^ and Valéria Pagotto^3^

^1^ *Health Sciences Graduate Program of the School of Medicine of the Federal University of Goiás;* ^2^ *Professor at Faculty Unida Campinas;* ^3^ *Nursing Graduate Program of the University Federal of Goiás, Brazil*

**Introduction**: Sarcopenia is associated with mortality however reaming unclear whether anthropometric measure of muscle mass such mid‐arm muscle circumference (MAMC) and calf circumference (CC) may be mortality predictors in older adults. We examined the impact of MAMC and CC in mortality in obese elderly and non‐obese older adults.

**Methods**: This is a longitudinal cohort study which data were extracted from the ‘Elderly Project/Goiania’. MAMC was calculated using the standard formula: MAMC = mid‐arm circumference − (0.314 × triceps skinfold thickness). We divided the MAMC into tertiles, and the lowest tertile was set as the reference group. Cut‐off points to define low CC were <34 cm for men and <33 cm for women. We used specific older adults body mass index (BMI) classification which obesity status was BMI >27.0 kg/m^2^. Data mortality was collected from the Brazilian Mortality Information System of the Health Ministry. We used Cox proportional hazards regression to estimate the hazard ratio (HR) of these anthropometric indices and mortality.

**Results**: Of the 416 participants, 66.1% were women, mean age 70.1 ± 7.1 years. During a mean 8.5‐year follow‐up period, we observed 144 all‐causes deaths (34.62% of the total cohort). Mean MAMC was 24.4 ± 3.12, and mean CC was 34.3 ± 3.4. Obesity prevalence was 49.3% (95% CI: 44.45–54.10). Men and women differed in their MAMC (25.8 ± 2.77 vs. 23.6 ± 3.0; *P* < 0.01, respectively) but not in CC measure (*P* = 0.299). The prevalence of low MAMC was higher among women than men (43.3%, 95% CI: 37.38–49.160 vs. 14.2%, 95% CI: 8.35–20.01; *P* < 0.01). Cox proportional regression analyses for all‐cause mortality in obese older adults with low MAMC was HR = 1.89, 95% CI: 1.01–3.55; *P* = 0.016, but not associated in non‐obese (*P* = 0.127). All‐cause mortality is associated with low CC in non‐obese (HR = 2.22, 95% CI: 1.33–3.69; *P* = 0.001) but not in obese older adults (*P* = 0.055).

**Conclusions**: These findings indicate that muscle mass by anthropometric measures has different impact in all‐cause mortality according to nutritional status. Low MAMC in obese and low CC in non‐obese are strong predictors of all‐cause mortality risk among non‐institutionalized older adults.

**5-07**

**Comparison between sarcopenia and frailty as predictors of post‐operative complications: a cohort study**

**Astrid Bicamumpaka Shema** ^1^, Maude Bouchard^1^, Laurence Bellemare^2^, Jonathan Afilalo^3^, Louis‐Antoine Mullie^1^, Marie‐France Forget^1^ and Han Ting Wang^4^

^1^ *Université de Montréal, Montreal, QC, Canada;* ^2^ *McGill University, Montreal, QC, Canada;* ^3^ *Division of Cardiology, Sir Mortimer B. Davis Jewish General Hospital, McGill University, Montreal, QC, Canada;* ^4^ *Department of Medicine, Division of Internal Medicine and Critital Care Medicine, Centre de Recherche Hôpital Maisonneuve Rosemont, Montreal, QC, Canada*

**Background:** Although sometimes used interchangeably, sarcopenia and frailty represent different concepts. Their correlation and whether one is a better predictor of post‐operative adverse outcomes have not been well studied. To evaluate the correlation between psoas muscle area (PMA), used as a surrogate for sarcopenia, and the Clinical Frailty Scale (CFS) in a cohort of non‐cardiac surgery patients. Our secondary objective is to evaluate the association between low PMA and the occurrence of post‐operative complications.

**Methods:** We conducted a retrospective observational cohort study. Patients 65 years and older undergoing elective non‐cardiac surgery in a tertiary academic centre were included. We collected post‐operative complications as defined by the National Surgical Quality Improvement Program. Right and left psoas areas were measured on CT scan at the level of the fourth lumbar vertebra body using the CORESLICER software. The total psoas area was calculated by adding left and right psoas areas and normalized for height. Patients were divided into tertiles according to PMA, the lowest tertile representing the low PMA group. Correlation between CFS and PMA was analysed using the Spearman correlation, and we performed a negative binomial regression analysis to assess the association between CFS and PMA and post‐operative complications.

**Results:** A total of 78 patients were included. The median value of corrected PMA was 7.15 [interquantile range (IQR): 2.23]. The median age was 72 years old (IQR: 7) and median Charlson Co‐morbidity Index was 6 (IQR: 3). The correlation coefficient between PMA and CSF was −0.256 (*P* = 0.031). In multivariate analysis, low PMA was significantly associated with increasing number of complications [incidence rate ratio (IRR): 3.53, 95% confidence interval (CI): 1.23–10.13, *P* = 0.019] while CFS was not (IRR: 0.22, 95% CI: 0.02–2.25, *P* = 0.199).

**Conclusions:** In our cohort, correlation between PMA and CFS was weak, and PMA was a better predictor of post‐operative complications than CFS.

**5-08**

**Sarcopenia as a mortality predictor in community‐dwelling older adults: a comparison of the diagnostic criteria of the European Working Group on Sarcopenia in Older People**

Nathalia P. Bachettini^1^, Renata M. Bielemann^2,3,4^, Thiago G. Barbosa‐Silva^4,5^, Ana Maria B. Menezes^4^, Elaine Tomasi^4^ and **M. Cristina Gonzalez** ^1,2,4^

^1^ *Post‐graduate Program in Health and Behavior, Catholic University of Pelotas, Pelotas, Brazil;* ^2^ *Post‐graduate Program in Nutrition and Foods, Federal University of Pelotas, Pelotas, Brazil;* ^3^ *Post‐graduate Program in Physical Education, Federal University of Pelotas, Pelotas, Brazil;* ^4^ *Post‐graduate Program in Epidemiology, Federal University of Pelotas, Pelotas, Brazil;* ^5^ *Department of Surgery, School of Medicine, Federal University of Pelotas, Pelotas, Brazil*

**Introduction**: The definition of sarcopenia remains a matter of discussion, and there is no globally accepted consensus for its diagnosis. The aim of this study was to assess the effect of sarcopenia diagnostic components on mortality, as well as to compare the associations between sarcopenia diagnosed via the 2010 and 2018 Consensuses of the European Working Group on Sarcopenia in Older People (EWGSOP) and mortality.

**Methods**: Prospective cohort study involving non‐institutionalized older adults aged ≥60 years. For the diagnosis of sarcopenia, the definitions proposed by the 2010 (EWGSOP) and 2018 (EWGSOP2) Consensuses were used. The diagnostic components corresponded to muscle mass, muscular strength, and physical performance. The associations of sarcopenia and its components with mortality were investigated using Cox proportional hazard regression models.

**Results**: The sample consisted of 1291 older adults. After an average of 2.6 years of follow‐up, 88 (6.8%) participants had died. The diagnosis of severe sarcopenia by both consensuses was associated with an increased risk of mortality. Severe sarcopenia was associated with an increased risk of death compared to that in people without sarcopenia when using EWGSOP [hazard ratio (HR) 3.15, 95% confidence interval (CI): 1.44–6.90] and EWGSOP2 (HR 4.11, 95% CI: 1.88–9.00). Older adults with decreased gait speed had a 76% higher risk of dying (*P* = 0.033). There was no statistically significant association between the other sarcopenia components and mortality risk.

**Conclusions**: Older adults with severe sarcopenia and those with changes in physical performance had an increased risk of death in the short term.

**5-09**

**SARC‐Calf as a predictive mortality tool for hospitalized elderly: results SarcDay 2018**

**M. Cristina Gonzalez** ^1,2,3^, Thiago Gonzalez Barbosa‐Silva^1,2,3^, Silvana Paiva Orlandi^2,3^, Letícia Fuganti Campos^3^ and Myrian Najas^4,5^

^1^ *Universidade Católica de Pelotas, RS, Brazil;* ^2^ *Universidade Federal de Pelotas, RS, Brazil;* ^3^ *Grupo de Estudos em Composição Corporal e Nutrição (COCONUT);* ^4^ *Universidade Federal do Paraná, PR, Brazil;* ^5^ *Universidade Federal de São Paulo, SP, Brazil*

**Introduction**: SarcDay is a project which intend to increase the awareness of the sarcopenia risk and its consequences in hospitalized elderly patients. In a 1‐day audit, all the elderly patients hospitalized in the last 72 h will have the sarcopenia risk assessed using SARC‐Calf and their outcome after 30 days. The objective of this study is to verify the association of SARC‐Calf and mortality.

**Methodology:** Multicentre observational study, with assessment of elderly patients (≥60 years) hospitalized in the last 72 h. Incapacity of answering the questions or calf circumference (CC) measurement was considered exclusion criteria. SARCF questions and low CC (<33 cm for male and <32 cm for female) were used to create SARC‐Calf^1^ (score ≥ 11 = sarcopenia risk). Other information, such as diagnostic and previous hospitalizations, were also collected. Outcome (mortality, still hospitalized, or hospital discharging) was verified after 30 days.

**Results:** A total of 162 patients was analysed (72.9 ± 10.2 years; 50.6% female). Among these patients, 18% had a previous hospitalization in the last 30 days, with a median length of stay of 7 days [interquartile range (IQR): 1–60] and 13.6% was bedridden at home. After 30 days, 133 patients were discharged (82%) from the hospital, and 17 patients (10.5%) died. Low CC and sarcopenia risk were found in 44% and 40% of the sample, respectively. Among the patients who died, 77% had SARC‐Calf ≥11. The mortality in patients with sarcopenia risk was five times higher than in patients without risk (20.3% vs. 4.1%; RR: 4.98, 95% CI: 1.70–14.59).

**Conclusions:** SarcDay showed that sarcopenia risk is already present in the first 72 h of hospitalization in elderly patients, and this risk is associated with a higher mortality. SARC‐Calf is a simple tool to identify the higher risk patients who will need an early intervention during the hospitalization.

**Reference**

1. Barbosa‐Silva TG, Menezes AM, Bielemann RM, Malmstrom TK, Gonzalez MC; Grupo de Estudos em Composição Corporal e Nutrição (COCONUT). Enhancing SARC‐F: Improving sarcopenia screening in the clinical practice. J Am Med Dir Assoc. 2016 Dec 1; **17**(12):1136‐1141.

**5-10**

**Impacts of cardiovascular disease risk on different status of sarcopenia in the community‐dwelling old adults**

**Tung‐Wei Kao**, Chung‐Ching Wang, Li‐Wei Wu and Wei‐Liang Chen

*Division of Geriatric Medicine, Department of Family and Community Medicine, Tri‐Service General Hospital; and School of Medicine, National Defense Medical Center, Taipei, Taiwan*

**Introduction:** Increasing evidences explored that sarcopenia was an important parameter for cardiometabolic risks. Different compositions of muscle quantity and muscle quality represented different status of sarcopenia. The objective of this study was to investigate whether different status of sarcopenia had different impacts on cardiovascular risk among male and female old adults in the community.

**Methods:** Old people aged 65 and more attended annually health examination from 2015 to 2018 in one medical centre in Taiwan with completion of a questionnaire, measurements of muscle mass, and muscle quality were recruited. The average value of the dominant handgrip strength was estimated three times by an analogue isometric dynamometer. Six‐meter distance walking time was recorded to calculate gait speed. Participants with normal muscle mass, but low muscle function was defined as dynapaenia. Definitions of pre‐sarcopenia and sarcopenia were based on the European consensus in 2010. The estimation of 10‐year Coronary Heart Disease Risk Scores (CHDRS) was calculated based on the Framingham score of Framingham Study. Logistic regression was used to estimate the odds of high CHDRS among different status of sarcopenia in both gender.

**Results:** A total of 709 elderly participants included (305 men). In old men with dynapaenia (*n* = 47) had 17.70 ± 5.08% CHDRS. In old women with sarcopenia (*n* = 74) had 7.74 ± 6.06% CHDRS. Participants with pre‐sarcopenia had the lowest CHRDS in both gender (15.41 ± 5.35% in men and 5.25 ± 3.70% in women). The CHDRS in the participants with dynapaenia was significant higher than sarcopenia in men [odds ratio (OR) = 2.52, 95% confidence interval (CI) = 1.04–6.09]. The CHDRS in the participants with sarcopenia was significant higher than dynapaenia in women (OR = 2.84, 95% CI = 1.11–7.25).

**Conclusions:** Old men with dynapaenia had higher risk of 10‐year coronary heart disease; nevertheless, this higher risk existed in old women with sarcopenia. Old adults with pre‐sarcopenia had the lowest coronary heart disease risk in both gender.

**5-11**

**Sarcopenic obesity and its association with frailty and protein‐energy wasting in haemodialysis patients: preliminary data from a single centre in Japan**

**Masakazu Saitoh** ^1,2^, Masumi Ogawa^2^, Hisae Kondo^2^, Kiichi Suga^2^, Haruki Itoh^3^ and Yoichiro Tabata^2^

^1^ *Department of Rehabilitation, Sakakibara Heart Institute, Tokyo, Japan;* ^2^ *Meiseikai Toyo Clinic Yachimata, Chiba, Japan;* ^3^ *Department of Cardiology, Sakakibara Heart Institute, Tokyo, Japan*

**Introduction:** This study investigated the prevalence of sarcopenia or sarcopenic obesity and their association with frailty and protein‐energy wasting (PEW) in haemodialysis patients.

**Methods:** The present study enrolled 117 adult haemodialysis patients (35% female, 64 ± 12 years old) from single units of a haemodialysis centre. The patients were divided into four groups: normal, obese, sarcopenia, and sarcopenic obesity. Sarcopenia was diagnosed by Asian Working Group for Sarcopenia (AWGS) criteria, and obesity was defined as an extensive per cent body fat mass greater than 40% in women and 30% in men. Skeletal muscle mass and per cent fat mass were evaluated by multifrequency whole‐body bioimpedance electrical analysis (seca 515, Japan) after a midweek dialysis session. Participants completed the Kihon Checklist and the criteria proposed by the International Society of Renal Nutrition and Metabolism expert panel to classify frailty and PEW. We performed multivariate logistic regression analysis to identify the clinical risk of frailty and PEW in patients with sarcopenia or sarcopenic obesity.

**Results:** Forty‐six (39.3%) patients were classified as normal; 18 (15.4%) as obese; 35 (29.9%) as having sarcopenia; and 18 (15.4%) as having sarcopenic obesity. In the multivariate analysis, the sarcopenic obesity group had a significantly higher risk of frailty than the normal group in the multivariate analysis after adjusting for age and gender (OR 4.518, 95% CI 1.218–16.752, *P* = 0.024). However, sarcopenic obesity was not associated with a higher likelihood of PEW, and sarcopenia imposed a significantly higher risk of PEW (OR 4.272, 95% CI 1.157–15.778, *P* = 0.029) than that in the normal group after adjusting for confounding factors.

**Conclusions:** Sarcopenic obesity was closely associated with frailty but not with PEW compared with the normal condition in haemodialysis patients.

**5-12**

**Differences of clinical and vascular parameters between dippers and non‐dippers in patients with maintenance haemodialysis**

**Su‐Hyun Kim** ^1^, Jung Ho Shin^1^, Jin Ho Hwang^1^, Hye Ryoun Kim^2^, Cheul Hong Min^3^, Seok Hui Kang^4^, Miyeun Han^5^, Ran‐hui Cha^6^ and Jun Chul Kim^7^

^1^ *Department of Internal Medicine, Chung‐Ang University Hospital, Seoul, Korea;* ^2^ *Department of Laboratory Medicine, Chung‐Ang University Hospital, Seoul, Korea;* ^3^ *Department of Internal Medicine, Sungae Hospital, Seoul, Korea;* ^4^ *Department of Internal Medicine, Yeungnam University Hospital, Daegu, Republic of Korea;* ^5^ *Department of Internal Medicine, Pusan National University Hospital, Pusan, South Korea;* ^6^ *Department of Internal Medicine, National Medical Center, Seoul, Korea;* ^7^ *Department of Internal Medicine, CHA Gumi Medical Center, CHA University, Gumi, Gyeongsangbuk‐do, Republic of Korea*

**Background:** Cardiovascular disease is the leading cause of death in ESRD patients. The cardiovascular complications of non‐dippers are known to be greater than the dippers in the stratification of hypertensive patients. Oxidative stress plays key roles in developing cardiovascular disease and non‐dippers related with higher oxidative stress markers and lower antioxidant levels. The aim of this study was to compare clinical parameters and oxidative stress between dippers and non‐dippers in patients with maintenance haemodialysis.

**Methods:** A total of 55 patients who were on maintenance haemodialysis were enrolled, and 49 patients were analysed. All the participants were performed 24 h ambulatory blood pressure (ABP) monitoring to divide the patients into two groups: dippers and non‐dippers. Non‐dippers were defined by a nocturnal reduction in daytime systolic blood pressure of less than 10%. Bioimpedance analysis (BIA) and oxidative stress marker (myeloperoxidase, MPO) were performed with routine laboratory tests for baseline study.

**Results:** Dippers and non‐dippers were 11 and 38 patients, respectively. Age, body mass index, proportion of abnormal results in ABI, IMT, and all the laboratory tests were not different between the two groups. The mean systolic BPs in daytime were not different between dippers and non‐dippers (131 vs. 136 mmHg, *P* = 0.320). However, the nocturnal mean systolic BPs were significantly different between the two groups (112 vs. 137 mmHg, *P* < 0.001). The fat free mass, skeletal muscle mass, appendicular muscle mass index, body mass index, and body cell mass were not different between dippers and non‐dippers. The level of MPO was significantly low in non‐dippers than in dippers (2.52 vs. 1.49 ng/mL, *P* = 0.018).

**Conclusions:** This study showed that skeletal muscle mass and appendicular muscle mass index were not associated with the non‐dipping BP pattern, and MPO was significantly associated with non‐dipping BP pattern in haemodialysis patients.

**5-13**

**Low muscle mass in patients receiving haemodialysis: correlation with noncoronary vascular calcification and incidence of repeat vascular intervention**

**Kim Seok‐hyung**, Eun Young Noe, Kwang‐Ho Choi, Jong‐Woo Yoon and Hyunsuk Kim

*Hallym University Medical Center, Chuncheon Sacred Heart Hospital, Chuncheon, South Korea*

**Introduction:** According to recent studies, vascular calcification was negatively correlated with sarcopenia. We aimed to investigate the correlation between sarcopenia and quantified vascular calcification score (VCS) of the arm including vascular access and whether low muscle mass (LMM) is associated with incidence of repeat vascular intervention.

**Methods:** Non‐contrast arm CT scan including vascular access was taken. Later, VCS was measured by using Agatston method. Skeletal muscle mass was estimated using bioelectrical impedance in supine position. LMM was defined as patients with skeletal muscle mass at both legs normalized to height‐squared less than the median. Vascular calcification (VC) group was assigned to patients with a VCS of 500 or higher. Statistical differences between the two groups were determined using the Mann–Whitney *U*‐test for continuous variables and the *χ* ^2^ test for categorical variables. Univariate and multivariate logistic regression analyses were used to determine the association between LMM and VC.

**Results:** We enrolled stable 75 HD patients. In the total 75 patients, there were 42 men (56.0%), and the median age was 64 (58–72) years. All but two patients met the diagnostic criteria for sarcopenia defined by a previous study on Korean population. The median vintage of HD was 49.4 (32.1–99.2) months. There were no differences between the two groups (LMM vs. non‐LMM) in sex, ESRD aetiology, and type of vascular access. However, age and HD vintage were significantly older in LMM group. LMM was significantly associated with VC [HR 3.562 (1.341–9.463), *P* = 0.011]. After adjusting age, sex, HD vintage, systolic blood pressure, and diabetes, LMM was independently associated with VC [HR 10.415 (2.357–46.024), *P* = 0.002]. Moreover, this study showed significant association between repeat vascular intervention and LMM [HR 3.652 (1.135–11.749), *P* = 0.03].

**Conclusions:** We quantified the VC and found for the first time that it is associated with LMM. LMM may be suggested as a potential predictor of VC. LMM increased a risk of repeat vascular intervention.

**5-14**

**Phase angle (PA) and Barthel score as prognostic tools for sepsis and mortality in critically ill older patients**

**Ricardo Schilling Rosenfeld** ^1,3^, Maria Cristina Gonzalez^2^ and Roberto Alves Lourenço^3^

^1^ *Nutrition Support Team, Casa de Saude Sao Jose, Associacao Congregacao de Santa Catarina, Rio de Janeiro, Brazil;* ^2^ *Post‐graduate Program in Health and Behavior, Catholic University of Pelotas, Pelotas, Brazil;* ^3^ *Post‐graduate Program in Medical Science, Rio de Janeiro State University, Rio de Janeiro, Brazil*

**Introduction:** Loss of lean body mass and functionality are characteristics of aging and disease severity and maybe associated with prognosis. The objective of this study was to verify PA at 1st day (PA_1_) correlation with other prognostic scores, including Barthel score, and their association with sepsis and mortality in 28 (D_28_) and 60 (D_60_) days after ICU admission in critically older patients.

**Methods:** Patients, older than ≥60 years, under mechanical ventilation ≥48 h, length of stay ≥3 days, haemodynamically stable were studied. PA_1_ was measured by a single frequency bioelectrical impedance (BIA) after haemodynamic stability. Severity (APACHE II, SOFA, and SAPS III) and functional scales (Barthel Index) were assessed in the first 24 h. Mortality at 28 (D28M) and 60 days (D60M) were also assessed.

**Results:** Seventy‐three older patients were enrolled (mean age 80 ± 8.8 years, 53% female). Sepsis was present in 36 patients (49.3%). The mortality incidence was 37% and 41% at D_28_ and D_60_, respectively. There was only a significant correlation between PA_1_ and Barthel score (0.43, *P* < 0.001). PA_1_ was significantly lower in septic patients than non‐septic (3.36 ± 0.78 vs. 3.74 ± 0.90, respectively) and in patients who died at D_60_ (3.26 ± 0.85 vs. 3.73 ± 0.81, respectively), but there is no significant difference in PA_1_ between survivors or not at D_28_. From the other prognostic scores, only Barthel scores are significantly different between survivors and non‐survivors at D_60_ [median 100 and interquartile range (IQR): 60–100 and median 60 and IQR: 45–100, respectively).

**Conclusions:** PA_1_ showed a significant correlation only with Barthel score. PA_1_ could differentiate septic from non‐septic patients. From all the prognostic scores, only PA_1_ and Barthel score could differentiate survivors from non‐survivors at D_60_, but none of them could be used as a prognostic tool to identify mortality at D_28_.

**5-15**

**Low phase angle is associated with cirrhosis and low muscle mass in chronic hepatitis C patients**

Nataly Lopes Viana^1,2^, Tatiana Bering^1,2^, Kiara Gonçalves Dias Diniz^1,2^, Marta Paula Pereira Coelho^1,2^, Maria Isabel Toulson Davidson Correia^3^, Rosangela Teixeira^1,2,4^, Gifone Aguiar Rocha^5^ and **Luciana Diniz Silva** ^1,2,4^

^1^ *Programa de Pós‐Graduação em Ciências Aplicadas à Saúde do Adulto;* ^2^ *Ambulatório de Hepatites Virais do Instituto Alfa de Gastroenterologia do Hospital das Clínicas;* ^3^ *Departamento de Cirurgia;* ^4^ *Departamento de Clínica Médica;* ^5^ *Laboratório de Pesquisa em Bacteriologia da Faculdade de Medicina da Universidade Federal de Minas Gerais (UFMG), Brazil*

**Introduction:** Although the use of electrical bioimpedance (BIA) is impaired when patients with hepatic cirrhosis have ascites, oedema, and electrolyte disturbances, the measurement of phase angle (PhA) in this population has been shown to be superior to anthropometric and biochemical methods for early detection of malnutrition. The PhA reflects the cellular integrity and normal values (according to sex and age) indicate preserved cellular activity. In patients with chronic hepatitis C (CHC), the role played by PhA has not been completely clarified. To evaluate the prevalence of low PhA and its association with demographic, clinical, and nutritional variables in CHC.

**Methods:** We prospectively included 222 patients [mean age, 53.7 ± 11.7 years; men, 116 (52.3%); diabetes mellitus, 40 (18.0%); hypertension, 91 (41.0%); cirrhosis, 87 (39.2%); underweight (BMI, <18.5 kg/m^2^ for adults and <22 kg/m^2^ for elderly), 9 (4.1%)]. The diagnosis and staging of liver disease were based on clinical, biochemical, histological, and radiological criteria. The PhA values were classified into percentiles according to the age/sex and the 5th percentile was adopted as cut‐off point. Low muscle mass was defined as <15th percentile for mid‐upper‐arm muscle area (MAMA). Data were analysed in logistic regression models.

**Results:** Low PhA and reduced MAMA were identified in 52 (23.4%) and 55 (24.8%) patients, respectively. The aspartate aminotransferase to platelet ratio index (APRI) in cirrhotic and non‐cirrhotic patients was 3.4 ± 2.8 and 0.8 ± 0.7, *P* ≤ 0.001, respectively. In the multivariate analysis, adjusted for age, body mass index, and gender, low PhA was significantly and independently associated with cirrhosis (OR = 3.74; 95% CI = 1.68–8.31; *P* = 0.001) and low MAMA (OR = 5.66; 95% CI = 2.56–12.68; *P* ≤ 0.001).

**Conclusions:** Low PhA is associated with negative conditions such as cirrhosis and low muscle mass. Reduced PhA is associated with poor clinical and nutritional prognosis in CHC patients.

**5-16**

**Predictive accuracy of muscle wasting is improved by combination of anthropometric indicators with nutritional status score in patients with heart failure**

**Satoshi Katano** ^1^, Toshiyuki Yano^2^, Suguru Honma^3^, Katsuhiko Ohori^2,4^, Takuya Inoue^1^, Yuhei Takamura^1^, Ryohei Nagaoka^1^, Akiyoshi Hashimoto^2,5^, Masaki Katayose^6^ and Tetsuji Miura^2^

^1^ *Division of Rehabilitation, Sapporo Medical University Hospital, Sapporo, Japan;* ^2^ *Department of Cardiovascular, Renal and Metabolic Medicine, Sapporo Medical University School of Medicine, Sapporo, Japan;* ^3^ *Department of Rehabilitation, Sapporo Cardiovascular Hospital, Sapporo, Japan;* ^4^ *Department of Cardiology, Hokkaido Cardiovascular Hospital, Sapporo, Japan;* ^5^ *Division of Health Care Administration and Management, Sapporo Medical University School of Medicine, Sapporo, Japan;* ^6^ *Second Department of Physical Therapy, Sapporo Medical University School of Health Sciences, Sapporo, Japan*

**Background:** We aimed to investigate whether the predictive accuracy of muscle wasting (MW) in CHF is improved by adding an index of nutritional status to anthropometric indicators.

**Methods:** Data for 314 patients [median of 77 years (interquartile range, IQR: 68–83 years), female 49%] admitted to our institute for diagnosis and treatment of CHF were analysed. All patients underwent DEXA scans and anthropometric measurements including calf circumferences (CC) and mid‐arm circumferences (MAC) as well as nutritional screening using mini nutritional assessment short form (MNA‐SF). According to the Japanese cut‐off value, MW was defined as DEXA‐measured ASM index (ASMI) <6.87 kg/m^2^ in male and <5.46 kg/m^2^ in female.

**Results:** The prevalence of MW was 65.6%. MNA‐SF score was lower in patients with MW than those without [median of 7 points (IQR: 5–9 points) vs. 10 points (IQR: 8–11 points), *P* < 0.01]. ASMI was significantly correlated with CC (ρ = 0.738, *P* < 0.01) and MAC (ρ = 0.498, *P* < 0.01), respectively. The analyses of the ability to predict MW showed that C‐statistics of logistic regression models, which were constructed by the use of CC or MAC, were significantly increased by adding MNA‐SF score to those models (CC: 0.796 vs. CC + MNA‐SF: 0.825, *P* = 0.046; MAC: 0.738 vs. MAC + MNA‐SF: 0.791, *P* < 0.01). The cut‐off values of CC or MAC, calculated by ROC curve analyses, to predict MW were as follows: CC ≤33.5 cm in male and CC ≤30.3 cm in female or MAC ≤27.0 cm in male and ≤26.5 cm in female. Inclusion of MNA‐SF score (≤7 points) exerted significant improvement in positive predictive values of CC or MAC for prediction of MW (CC: 85% vs. CC + MNA‐SF: 94%, *P* = 0.02; MAC: 80% vs. MAC + MNA‐SF: 90%, *P* = 0.02).

**Conclusions:** Accuracy of anthropometric indicators‐based prediction of MW in CHF can be improved by combination of anthropometric indicators with nutritional assessment using MNA‐SF.

**5-17**

**Impact of the obesity degree in the densitometric diagnosis of sarcopenia**

Tatiana Munhoz da Rocha Lemos Costa, Renata Gonçalves Pinheiro Correa, Letícia Guadanhim Sampaio, Janaina Marques, Ricardo Rasmussen Petterle and **Victoria Zeghbi Cochenski Borba**

*Serviço de Endocrinologia ne Metabologia do Hospital de Clinicas da Universidade Federal do Parana (SEMPR), Curitiba, Brazil*

**Introduction:** Skeletal muscle metabolism changes may occur in obesity and may lead to altered body composition with higher fat mass and substantial impairment of muscle mass and quality. The degree of obesity may influence on the prevalence of muscle mass loss. The study purpose was to evaluate the presence of pre‐sarcopenia and sarcopenia in individuals with different degrees of obesity and to correlate the diagnosis with anthropometric data and characteristics of obesity.

**Methods:** The study included obese patients (BMI ≥ 30 kg/m^2^), ≥18 years old, excluding those in use of medications or co‐morbidities that affect lean mass. Patients answered questionnaires to assess demographic data, physical activity level, and health status. Anthropometric data collection, total body densitometry examination by dual X‐ray emission, and handgrip strength were performed. Sarcopenia was diagnosed by dynamometry and FNIH (Foundation for the National Institutes of Health) criteria for densitometry, and classified as normal (two normal criteria), pre‐sarcopenia (altered FNIH only), and sarcopenia (altered dynamometry plus FNIH). Patients were classified in degrees of obesity, as obesity I (GI), II (GII), and III (GIII), and compared to a control group (CG).

**Results:** The study selected 121 patients, 92.3% women, mean age 46.8 ± 14 years. The mean BMI was 37.1 ± 5.0 kg/m^2^ being GI (36.6%), GII (38.3%), and GIII (24%). Pre‐sarcopenia was observed in 33 (27.2%) obese patients (35 women) compared to 4 (3.5%) individuals in CG, *P* < 0.001. There was a positive association between pre‐sarcopenia and degree of obesity (*P* < 0.001). Sarcopenia was present in 6 (4.9%) obese patients and none of CG, and it was correlated with the higher percentage of android fat (*P* = 0.04), with no correlation between BMI and age.

**Conclusions:** Sarcopenia was prevalent in all degrees of obesity, and there is still no well‐established criterion for the diagnosis of obesity sarcopenia in the literature.

**5-18**

**Body composition analysis in obese patients using bioelectrical impedance**

**Nadja Vasiljevic**, Dragana Davidovic, Branko Jakovljevic and Milos Maksimovic

*Institute of Hygiene and Medical Ecology, Faculty of Medicine, University of Belgrade, Dr Subotica 8, Belgrade, Republic of Serbia*

**Introduction:** The aim of this study was to evaluate body composition variables: fat mass—FM, fat free mass—FFM, fat mass index—FMI, fat free mass index—FFMI, per cent body fat—PBF in relation to sex and age, in obese patients.

**Methods:** The study involved 1001 subjects of both sexes—46% men and 54% women, who were treated for obesity at the Department of Nutrition. Body composition was evaluated by Tanita BC 418 MA bioelectric impedance.

**Results:** Results revealed the highest prevalence of obesity II and higher degree (BMI ≥35 kg/m^2^)—42.4% in men and obesity I degree (BMI 30–34.9 kg/m^2^) in women—35.7%. According to the different age categories BMI values are significantly different only among women (*P* < 0.004). Values of BMI and FM in the age range are homogenous among men, but only FM values are homogenous among women. FFM and FFMI are significantly higher in younger men and decreasing with advancing ages. Among women, FM, FMI, and PBF are increasing with age, while FFM is significantly decreasing (*P* < 0.001). Correlation analysis revealed that there is a positive association of FMI, FFMI, and PBF with obesity degree in relation to age. Contrary, among women, there is a significant correlation of FMI and PBF with obesity degree but not for FFMI.

**Conclusions:** Bioelectrical impedance assessment of body composition contributes to the precise determination of the body composition especially of FFM and FM. It should be applied at the beginning and during the monitoring of medical treatment for obesity in the aim of preservation of muscle mass and its function and the prevention of sarcopenic obesity.

**5-19**

**GDF‐15 circulating levels in cancer patients with anorexia and sarcopenia**

**Alessio Molfino** ^1^, Maria Ida Amabile^1,2^, Giuseppe Nigri^3^, Roberta Belli^1^, Cesarina Ramaccini^1^, Antonio di Renzo^1^, Giovanni Imbimbo^1^, Paola Costelli^4^ and Maurizio Muscaritoli^1^

^1^ *Department of Translational and Precision Medicine, Sapienza University of Rome, Rome, Italy;* ^2^ *Department of Surgical Sciences, Sapienza University of Rome, Rome, Italy;* ^3^ *Department of Medical‐Surgical Sciences and Translational Medicine, Sapienza University of Rome, Rome, Italy;* ^4^ *Department of Clinical and Biological Sciences, Interuniversity Institute of Myology, University of Turin, Turin, Italy*

**Introduction:** The pathophysiology of cancer anorexia and sarcopenia is not completely clarified. Different serum biomarkers, including some growth and differentiation factors, may be modulated during cancer and likely associated with an anorexic and sarcopenic phenotype. The aim of our study was to explore the potential association(s) between the serum levels of GDF‐15, anorexia, and sarcopenia in patients with gastrointestinal (GI) and lung cancer.

**Methods:** We considered patients with gastric, pancreatic, colorectal, and lung cancer, at their first diagnosis, with or without anorexia and sarcopenia, and healthy matched controls. The Functional Assessment of Anorexia/Cachexia Therapy (FAACT) questionnaire was administered to all patients to detect the presence of anorexia. Radiological evaluation of skeletal muscle mass by computed tomography scan (L3‐SMI) was used to diagnose sarcopenia in cancer patients and GDF‐15 serum levels were assessed by ELISA (enzyme‐linked immunosorbent assay) in all the participants. Parametric and non‐parametric tests were performed, appropriate. A value of *P* < 0.05 was considered statistically significant.

**Results:** In total, 38 cancer patients and 21 controls were enrolled (25 with GI cancer and 13 with lung cancer). GDF‐15 serum levels (Ln) resulted significantly higher in cancer patients with respect to controls (7.16 ± 0.74 vs. 6.58 ± 0.66; *P* = 0.002). Moreover, GI cancer patients showed higher GDF‐15 levels with respect to lung cancer patients (*P* = 0.001) and to controls (*P* < 0.001). The prevalence of anorexia (FAACT ≤ 30) in cancer patients was 73.7% (*n* = 28). Patients with a higher degree of anorexia (FAACT ≤ 21) were 34.2% (*n* = 13) and showed higher GDF‐15 levels with respect to those with FAACT > 21 (*n* = 25) (7.52 ± 0.91 vs. 6.97 ± 0.56; *P* = 0.03). The prevalence of sarcopenia in cancer patients was 52.6% (*n* = 20). Sarcopenic patients showed higher GDF‐15 levels compared to non‐sarcopenic cancer patients (7.42 ± 0.87 vs. 6.88 ± 0.43, *P* = 0.02). Patients with GI cancer had a lower FAACT score compared to lung cancer patients (23.38 ± 6.71 vs. 31.08 ± 6.16, *P* = 0.002) as well as a lower value of L3‐SMI (33.72 ± 8.96 vs. 49.22 ± 5.7, *P* < 0.001).

**Conclusions:** Our data suggest an involvement of GDF‐15 in the pathogenesis of cancer‐associated anorexia and sarcopenia. To confirm these data, it is necessary to conduct studies on larger populations of cancer patients.

**5-20**

**Plasma IL‐6 is a sarcopenia marker in patients with colorectal cancer**

**Simone Heisz** ^1^, Olga Prokopchuk^2^, Tanja Krauss^1^, Klaus‐Peter Janssen^2^, Melina Claussnitzer^3,4^, Claudine Seeliger^1^, Marc Martignoni^2^ and Hans Hauner^1^

^1^ *Else Kröner‐Fresenius‐Center of Nutritional Medicine, Technical University of Munich, Freising, Germany;* ^2^ *Department of Surgery, Klinikum rechts der Isar, Technical University of Munich, Munich, Germany;* ^3^ *Institute of Nutritional Science, University of Hohenheim, Stuttgart, Germany;* ^4^ *Broad Institute of MIT and Harvard, Cambridge, MA, USA*

**Introduction:** Cachexia is a multifactorial and multi‐organ wasting syndrome, which causes not only sarcopenia but also loss of fat mass. To characterize cancer cachexia and to obtain biomarkers, we focused on patients suffering from malign diseases of the gastrointestinal tract.

**Methods:** The patient cohort (*n* = 189) was recruited at the Klinikum rechts der Isar, TUM. For analysis, patients were stratified according to tumour entity, cachectic, and sarcopenic status. Cachexia was defined as 10% or more weight loss during 6 months preceding surgery. Sarcopenia was defined via CT scans by consensus thresholds as skeletal muscle area index (SMAI) < 52.4 cm^2^/m^2^ for men and <38.5 cm^2^/m^2^ for women (Prado, Lieffers et al. 2008). Blood plasma levels of circulating interleukin‐6 (IL‐6) were measured by ELISA and matched to clinical data.

**Results:** Out of 189 patients, 147 suffered from gastrointestinal carcinoma including pancreatic ductal adenocarcinoma, colorectal adenocarcinoma (CRC), stomach, or oesophageal cancer. Twenty‐five patients with benign diseases and 17 patients without tumour or inflammatory diseases served as controls. IL‐6 was significantly higher in cancer patients versus control patients (*P* = 0.043). CRC patients revealed higher IL‐6 compared to other tumour entities and controls. Furthermore, IL‐6 was significantly increased in CRC patients with sarcopenia compared to those without (*P* = 0.022). There was a significant negative correlation between plasma IL‐6 and SMAI in CRC male patients (*R* = −0.437; *P* = 0.023). However, no significant differences could be found in IL‐6 plasma levels between cachectic and non‐cachectic patients.

**Conclusions:** In CRC patients, the proinflammatory cytokine IL‐6 may be a suitable marker for sarcopenia, but not for cachexia in general. To link cachexia and sarcopenia to the inflammatory state under tumour burden on a molecular level, further investigations are needed.

**5-21**

**Comparison of EWGSOP2 and EWGSOP1 for their impact on the capability of sarcopenia to predict prognosis after radical gastrectomy: analysis from a large‐scale prospective study**

**Cheng‐Le Zhuang** ^1^, Su‐Lin Wang^2^, Xian Shen^3^ and Zhen Yu^1^

^1^ *Department of Gastrointestinal Surgery, Shanghai Tenth People's Hospital Affiliated to Tongji University, Shanghai, China;* ^2^ *Department of Gastrointestinal Surgery, The First Affiliated Hospital, Wenzhou Medical University, Wenzhou, China;* ^3^ *Department of Gastrointestinal Surgery, The Second Affiliated Hospital, Wenzhou Medical University, Wenzhou, China*

**Introduction:** In 2010, the European Working Group on Sarcopenia in Older People (EWGSOP) reached a consensus on sarcopenia (EWGSOP1). In 2018, the EWGSOP met again (EWGSOP2) to update original definition of sarcopenia. This study aimed to investigate the association of sarcopenia and survival and compare the prognostic effects of sarcopenia as defined by EWGSOP1 and EWGSOP2 after gastrectomy.

**Methods:** We conducted a prospective study including patients who underwent curative gastrectomy for gastric cancer from August 2014 to February 2018. The sarcopenia elements, including skeletal muscle index, muscle attenuation, handgrip strength, and gait speed were measured before surgery. Patients were followed up after gastrectomy to gain the actual clinical outcomes.

**Results:** The prevalence of sarcopenia was 17.0% and 18.9% according to the EWGSOP1 and EWGSOP2, respectively. Sarcopenia was independent risk factor for post‐operative complications. Compared with EWGSOP1‐sarcopenia, EWGSOP2‐sarcopenia had a higher odds ratio (OR) (2.453 vs. 1.550) in multivariate model. Area under the ROC curve of model including EWGSOP2‐sarcopenia was larger than that of the model including EWGSOP1‐sarcopenia (AUC 0.653 vs. 0.634, *P* = 0.021). For both of EWGSOP1 and EWGSOP2, sarcopenia was an independent risk factor for overall survival (OS) and disease‐free survival (DFS), but EWGSOP2‐sarcopenia seemed to have a higher hazard ratio (OS, 1.667 vs. 1.449; DFS, 1.603 vs. 1.563). In addition, severe sarcopenia, as defined by either EWGSOP2 or EWGSOP1, had a strong predictive power (OR 4.909 vs. 3.827) for post‐operative complications. Both versions of severe sarcopenia were significantly predictive of OS and DFS in Cox analysis.

**Conclusions:** Sarcopenia at uniform diagnosis standard was an independent risk factor for survival in patients undergoing radical gastrectomy for gastric cancer. The EWSGOP2 improved the capability of sarcopenia to predict prognosis.

**5-22**

**The impact of computed tomography‐defined sarcopenia on survival in adult patients undergoing radiotherapy ± other treatment modality of curative intent for head and neck cancer: a systematic review and meta‐analysis**

**Merran Findlay** ^1,2,3^, Kathryn White^3^, Natalie Stapleton^4^ and Judith Bauer^5^

^1^ *Cancer Services, Royal Prince Alfred Hospital, Sydney Local Health District, Sydney, NSW, Australia;* ^2^ *The Chris O'Brien Lifehouse, Sydney, NSW, Australia;* ^3^ *Susan Wakil School of Nursing and Midwifery, Faculty of Medicine and Health, University of Sydney, NSW, Australia;* ^4^ *Cancer Council New South Wales, Sydney, NSW, Australia;* ^5^ *School of Human Movement and Nutrition Science, The University of Queensland, QLD, Australia*

**Introduction:** Computed tomography (CT)‐defined sarcopenia is a demonstrated poor prognostic factor for survival in patients with cancer; however, its impact in patients with head and neck cancer (HNC) has not been established. This study aimed to synthesize current knowledge and determine the prognostic impact of CT‐defined sarcopenia on survival in patients with HNC undergoing radiotherapy ± other treatment modality of curative intent.

**Methods:** A systematic review of the literature published between January 2004 and June 2019 was conducted in Medline, Embase, CINAHL, AMED, and PubMed. Empirical studies in adults (≥18 years) who had completed radiotherapy of curative intent ± other treatment modalities that evaluated sarcopenia using the gold standard method at the third lumbar vertebra and applied sex‐specific cut‐offs were included. Outcome of interest was overall survival. Study quality was assessed using the Cochrane Risk of Bias in Non‐Randomized Studies of Interventions (ROBINS‐I). Hazard ratios with 95% confidence intervals derived from multivariate analysis were extracted directly from studies. Random effects meta‐analysis was used to determine the pooled hazard ratio for overall survival in patients with sarcopenia versus those without using RevMan (version 5.3). The certainty of evidence was assessed using the Grading of Recommendations, Assessment, Development, and Evaluation (GRADE) system.

**Results:** The five studies (*n* = 3462) that met the inclusion criteria all defined sarcopenia as low muscle mass but varied in skeletal muscle index threshold (SMI) values applied and ethnicity. Sarcopenia prevalence ranged from 6.6% to 70.9% pre‐treatment and from 12.4% to 65.8% post‐treatment. Pre‐treatment sarcopenia was associated with reduced overall survival (HR 2.47; 95% CI, 1.78–3.43, *P* < 0.001, *I* ^2^ = 26%). The certainty of evidence for overall survival according to GRADE was moderate.

**Conclusions:** CT‐defined sarcopenia impacts negatively on overall survival in patients with HNC and holds a clinically meaningful prognostic value. Consensus regarding sarcopenia assessment and SMI threshold values is warranted.

**Conflict of interest:** Chief Investigator (M Findlay) was supported by a Sydney Research PhD Scholarship. The contribution of all authors is free of any conflict of interest.

**5-23**

**Sarcopenic obesity and metabolic syndrome: what is the impact in the context of liver transplantation?**

Mimosa Nguyen^1^, Mélanie Tremblay^2^, Geneviève Huard^3^, An Tang^3^, Chrisopher F. Rose^2^ and **Chantal Bémeur** ^1,2^

^1^ *Department of Nutrition, Université de Montréal, Montreal, Quebec, Canada;* ^2^ *Centre de Recherche du Centre Hospitalier de l'Université de Montréal, Montreal, Quebec, Canada;* ^3^ *Centre Hospitalier de l'Université de Montréal, Montreal, Quebec, Canada*

**Introduction:** Sarcopenia is associated with a worst prognosis in cirrhotic patients after liver transplantation (LT). As patients gain weight and sarcopenia remains after LT, sarcopenic obesity (SO) develops. Metabolic syndrome (MS), a cluster of factors that increase the risk of heart disease and diabetes, is caused by weight gain. There are limited data about the influence of SO and MS in LT recipients. The goal of this study was to examine the impact of SO and MS on outcomes after LT.

**Method:** In total, 94 cirrhotic patients who underwent LT at the CHUM—Liver Unit were included. Sarcopenia was assessed at the third lumbar level vertebrae using a CT scan. Obesity was determined using BMI whereas MS was diagnosed using the presence of ≥3 modified NCEP ATP III criteria. The prognostic factors were collected 6 months before and during 1 year after LT through medical records and included number of complications, episodes of infections, length of stay, and frequency of readmissions.

**Results:** Most of the patients (~70%) were not obese before LT. Approximately 20% of the patients developed obesity after LT. Among patients who were obese before LT, ~40% of the patients remained obese after LT. SO affected 10% and less of the patients before and after LT. Among patients with MS before LT (64%), ~40% of them was still affected after LT. Among patients who were not affected by MS before LT, 38% developed MS after LT and one patient remained not affected after LT. Prognostic factors were worst in patients with SO and MS before and after LT.

**Conclusions:** SO affected a small proportion of patients while MS was prevalent before and after LT. Nevertheless, these conditions were associated with worst prognosis. Strategies to manage SO and MS could help to improve recovery in patients who have undergone LT.

**6-01**

**Biostatistical approaches for drug repositioning: NUDT3 & KLF5 for lean mass & HLA‐DQB1‐AS1 for handgrip skeletal muscles traits and their associated SNPs as candidate targets**

**Abhishek Narain Singh** ^1,2^, Bili Gasman^2^ and David Karasik^2^

^1^ *University of Eastern Finland;* ^2^ *Bar‐Ilan University, Israel*

**Introduction**: Diseases of muscles, specifically sarcopenia, are of clinical importance as sarcopenia is a common condition of old age. Widening the scope of knowledge in the field of muscle mass/strength genetics is important in the sense that it allows us to identify new genetic markers for musculoskeletal diseases or identify patients with an increased risk to develop a specific condition. It might also allow us to identify drugs that affect muscle in ways unknown before and therefore to reposition drugs to other uses, in accordance to their newly found target.

**Method**: The aim of this project was to show that a mutation in a specific locus is associated with muscle health phenotypes. We identified by statistical, bioinformatic tools loci responsible for regulating relevant genes for muscle health, which can then be a target for downstream lab experimentation validation. SNPs associated with various disease traits for the muscles and specific loci were chosen according to their muscle phenotype association *P*‐value, as traditionally done in the GWASs. We developed and applied a combination of expression quantitative trait loci study (eQTLs) and GWAS summary information, to prioritize causative SNP and point out the unique genes associated in the tissues of interest (muscle).

**Results and Conclusions**: We found NUDT3 and KLF5 for lean mass & HLA‐DQB1‐AS1 for handgrip skeletal muscles traits candidate target genes to target for these phenotypes. SNPs associated with these genes for regulation can then be seen in perspective of TADs and can be targeted for knock out in either C2C12 mouse myoblast cells, adipocytes, or any other relevant cell.

**6-02**

**Spsb1 is involved in inflammation‐induced muscle atrophy**

**Yi Li** ^1^, Melanie Kny^1^ and Jens Fielitz^1,2,3^

^1^ *Experimental and Clinical Research Center, Charité‐Universitätsmedizin Berlin, Max Delbrück Center for Molecular Medicine in the Helmholtz Association, Berlin, Germany;* ^2^ *Department of Internal Medicine B, Cardiology, University Medicine Greifswald, Germany;* ^3^ *DZHK (German Center for Cardiovascular Research), partner site Greifswald, Greifswald, Germany*

**Introduction:** Critically ill intensive care unit (ICU) patients often develop a significant loss of muscle weight leading to muscle weakness during their ICU stay (ICU acquired weakness, ICUAW). Sepsis is one of the major causes of ICUAW. However, exact mechanisms underlying muscle atrophy in ICUAW are not well defined. By RNA sequencing, we observed the up‐regulation of *Spsb1* in the tibialis anterior muscle of septic mice undergoing cecal ligation and puncture surgery. The SPRY domain‐ and SOCS box‐containing protein 1 (Spsb1) was shown to inhibit TGF‐β signalling by targeting TGF‐β receptor type‐2 (TβRII), but its relevance in muscle atrophy is unknown. Our objectives were to investigate the role of Spsb1 in inflammation‐induced muscle atrophy and identify the downstream targets and signalling pathways that contribute to muscle atrophy in cultivated myocytes.

**Methods:** The effects of Spsb1 on undifferentiated and differentiated cultured C2C12 skeletal muscle cells were studied by analysing gene expression, protein content, and the atrophy phenotype. Co‐immunoprecipitation assays were performed to identify downstream targets of Spsb1.

**Results:** Spsb1 over‐expression significantly impaired proliferation, differentiation, and fusion of C2C12 myoblasts, which resulted in the formation of atrophied myotubes. Myogenin, a critical transcription factor regulating myogenic differentiation, was down‐regulated in Spsb1 over‐expressed cells. The transcripts of *Mymk* and *Mymx*, which encode key factors that govern the myoblast fusion, were severely decreased by Spsb1 overexpression. A search for the molecular mechanisms revealed that the expression of TβRII was up‐regulated during differentiation and Spsb1 targeted TβRII for degradation.

**Conclusions:** Spsb1 is involved in myoblast fusion and differentiation which contributes to myotube atrophy.

**6-03**

**Deep learning method for localization and segmentation of abdominal CT**

Setareh Dabiri^1^, Karteek Popuri^1^, Elizabeth M. Cespedes Feliciano^2^, Bette J. Caan^2^, Vickie E. Baracos^3^ and **Mirza Faisal Beg** ^1^

^1^ *School of Engineering Science, Simon Fraser University, Canada;* ^2^ *Division of Research, Kaiser Permanente Northern California, USA;* ^3^ *Department of Oncology, University of Alberta, Canada*

**Introduction:** Computed tomography (CT) imaging is widely used for studying body composition, i.e. the proportion of muscle and fat tissues with applications in areas such as nutrition or chemotherapy dose design. Axial CT slices from the third lumbar (L3) vertebral location are commonly used for body composition analysis. However, selection of the third lumbar vertebral slice and the segmentation of muscle/fat in the slice is a tedious operation if performed manually.

**Methods:** We have designed a deep‐learning based method to automatically find the middle axial slice at L3 level from a full or partial body CT scan volume (network 1, N1) and segment the skeletal muscle (SM), subcutaneous adipose tissue (SAT), visceral adipose tissue (VAT), and intramuscular adipose tissue (IMAT) on that slice (network 2, N2). The localization network N1 is a fully convolutional classifier trained on more than 12 000 images. The segmentation network N2 is a convolutional neural network with an encoder–decoder architecture.

**Results:** Three datasets with CT images taken for patients with different types of cancers are used for training and validation of the networks. The mean slice error of 0.87 ± 2.54 was achieved for L3 slice localization on 1748 CT scan volumes. The performance of four class tissue segmentation network evaluated on two datasets with 1327 and 1202 test samples. The mean Jaccard score of 97% was achieved for SM and VAT tissue segmentation on 1327 images. The mean Jaccard scores of 98% and 83% are corresponding to SAT and IMAT tissue segmentation on the same dataset.

**Conclusions:** The localization and segmentation network performances indicate the potential for fully automated body composition analysis at the L3 level with high accuracy.

**6-04**

**Metabolic‐energetic profiles in myoblast cultures of patients with sarcopenia**

**Michael Drey** ^1^, Lisa Baber^1^, Fabiana Tanganelli^1^, Stefanie Jarmusch^1^, Fabian Hofmeister^1^, Carl Neuerburg^3^, Stefan Mehaffey^3^, Peter Meinke^2^, Uta Ferrari^1^ and Stefan Hintze^2^

^1^ *Department of Medicine IV, University Hospital, LMU Munich;* ^2^ *Friedrich Baur Institute at the Department of Neurology, University Hospital, LMU Munich;* ^3^ *Department of General, Trauma and Reconstructive Surgery, University Hospital, LMU Munich*

**Introduction:** Sarcopenia is a common disease in old age and can lead to falls and fragility fractures. It is unclear to what extent the muscle cell metabolism is affected in patients with sarcopenia with proximal femur fractures.

**Methods:** Muscle biopsies of the vastus lateralis muscle were taken from 38 patients (mean age: 81 years, 26 women) who underwent operative treatment by osteosynthesis or endoprosthesis implantation due to a proximal femur fracture. The myoblasts from primary cell cultures of the extracted biopsies were analysed for mitochondrial respiration and glycolysis using a Seahorse XFp Analyzer. The patients were examined by isometric handgrip strength measurement and bioelectrical impedance analysis for determination of the muscle mass index. According to the criteria of EWGSOP 2, a *z*‐score was calculated as a measure of the degree of sarcopenia. Data were analysed, using regression analysis, considered significant with a *P*‐value <0.05.

**Results:** There was a significant association between the degree of sarcopenia and the glycolytic capacity (β = −0.386, *P* = 0.020) and the glycolytic reserve (β = −0.497, *P* = 0.002). This relationship persisted after adjusting for the age of the patients. The effect was more pronounced for women than for men.

**Conclusions:** Sarcopenia seems to be associated with a limitation of metabolic–energetic glycolysis. A gender‐related effect was shown but requires further investigation due to the low number of men. Confirmation of our results could open a new door for therapeutic interventions of sarcopenia.

**6-05**

**A subset of canonical Wnt and Hippo pathway transcriptional regulators ensure physiological synaptic gene transcription at the neuromuscular junction**

**Said Hashemolhosseini**, Danyil Huraskin and Nane Eiber

*Friedrich‐Alexander‐Universität Erlangen‐Nürnberg, Institut für Biochemie, Erlangen, Germany*

We inquired the neuromuscular role of canonical Wnt/β‐catenin and Hippo signalling pathways and their nuclear effectors β‐catenin, YAP, TAZ, and members of the TCF, TEAD, and Groucho/TLE families. Denervation caused a muscular abrogation of canonical Wnt/β‐catenin and a gain in YAP/TAZ/TEAD signalling activity. Transcriptional profile changes in the myogenic lineage and in response to agrin pointed at potential importance of Tle3, Tle4, Tead1, and Tead4 at the post‐synapse. Knockouts of these genes via CRISPR/Cas9 gene editing led to reduced agrin‐dependent AChR clustering and diminished synaptic gene transcription in differentiated primary muscle cells. *In silico* analysis of previously reported TEAD1/4 genomic occupation sites revealed evolutionary conserved areas with potential TEAD binding motifs in important synaptic genes, which relevance was functionally confirmed by luciferase assays. Overall, our data point to a role of TLE3, TLE4, TEAD1, and TEAD4 in acetylcholine receptor clustering and regulating expression of synaptic genes at the neuromuscular junction.

**6-06**

**Muscle wasting in cancer involves suppression of ribosomal production and increased expression of the ribophagy receptor NUFIP1**

Hyo‐Gun Kim^1^, Joshua R. Huot^3^, Fabrizio Pin^4^, Andrea Bonetto^3^ and **Gustavo A. Nader** ^1,2^

^1^ *Department of Kinesiology;* ^2^ *Huck Institutes of the Life Sciences;* ^3^ *Department of Surgery, The Pennsylvania State University, State College, PA, USA;* ^4^ *Department of Anatomy and Cell Biology, Indiana University School of Medicine, Indianapolis, IN, USA*

**Introduction:** Muscle wasting/cachexia is a major contributor of mortality and morbidity in cancer and is frequently accompanied by functional and metabolic deficits. Muscle mass is determined by the balance between protein synthesis and degradation, yet while previous studies revealed that protein degradation plays an important role in muscle wasting, the contribution of anabolic deficits (i.e. lower ribosomal content) in muscle wasting in cancer remains understudied. Ribosomal content is regulated by transcription of the ribosomal (r)DNA genes and by degradation of ribosomes via ribophagy. We hypothesized that muscle loss in a previously described model of ovarian cancer cachexia is associated with reduced ribosomal mass.

**Methods:** Nod SCID gamma mice were injected with 1 × 10^7^ ES‐2 human high‐grade serous ovarian cancer cells for 14 days. Translational capacity was estimated by quantitating rRNA content, and the mechanisms of ribosomal production and degradation by measuring rDNA gene transcription rates, and expression of the ribophagy receptor Nuclear FMR1 Interacting Protein 1 (NUFIP1), respectively.

**Results:** Gastrocnemius mass was 24% (*P* < 0.0001) lower than control. Muscle wasting was associated with a ~50% (*P* = 0.0003) reduction in ribosomal capacity and a significant suppression in rDNA gene transcription rates (35%, *P* = 0.0086). This anabolic deficit also involved a four‐fold increased expression (*P* < 0.0001) of the ribophagy receptor NUFIP1.

**Conclusions:** In this model of ovarian cancer, a reduction in ribosomal mass may drive muscle wasting and is associated with deficits in ribosomal production. Additionally, the elevation in NUFIP1 expression suggests that during wasting conditions, degradation of muscle ribosomes may also contribute to a lower translational capacity and thereby exacerbate muscle wasting. Overall, these data suggest that in cancer cachexia, the ability of the muscle to synthesize protein is diminished in part due to reduced ribosome production and increased ribosome degradation.

**6-07**

**Skeletal muscle mTORC1 regulates neuromuscular junction stability**

Martina Baraldo^1,5^, Alessia Geremia^1,5^, Marco Pirazzini^5^, Leonardo Nogara^1,5^, Francesca Solagna^1,5^, Clara Türk^2^, Hendrik Nolte^2^, Vanina Romanello^1,5^, Aram Megighian^5^, Simona Boncompagni^4^, Marcus Kruger^2,3^, Marco Sandri^1,5^ and **Bert Blaauw** ^1,5^

^1^ *Venetian Institute of Molecular Medicine (VIMM), Padova, Italy;* ^2^ *Institute for Genetics, Cologne Excellence Cluster on Cellular Stress Responses in Aging‐Associated Diseases (CECAD), Cologne Germany;* ^3^ *Center for Molecular Medicine (CMMC), University of Cologne, Germany;* ^4^ *CeSI‐Met—Center for Research on Ageing and Translational Medicine and DNICS—Department of Neuroscience, Imaging and Clinical Sciences, University G. d'Annunzio, Italy;* ^5^ *Department of Biomedical Sciences, University of Padova, Padova, Italy*

**Background:** Skeletal muscle is a plastic tissue which can adapt to different stimuli. It is well established that mammalian target of rapamycin complex 1 (mTORC1) signalling is a key modulator in mediating increases in skeletal muscle mass and function. However, the role of mTORC1 signalling in adult skeletal muscle homeostasis is still not well defined.

**Methods:** Inducible, muscle‐specific Raptor and mTOR k.o. mice were generated. Muscles at 1 and 7 months after deletion were analysed to assess muscle histology and muscle force.

**Results:** We found no change in muscle size or contractile properties 1 month after deletion. Prolonging deletion of raptor to 7 months, however, leads to a very marked phenotype characterized by weakness, muscle regeneration, mitochondrial dysfunction, and autophagy impairment. Unexpectedly, reduced mTOR signalling in muscle fibres is accompanied by the appearance of markers of fibre denervation, like the increased expression of the neural cell adhesion molecule (NCAM). Both muscle‐specific deletion of mTOR or raptor, or the use of rapamycin, was sufficient to induce 3–8% of NCAM‐positive fibres (*P* < 0.01), muscle fibrillation, and neuromuscular junction (NMJ) fragmentation in 24% of examined fibres (*P* < 0.001). Mechanistically, reactivation of autophagy with the small peptide Tat‐beclin is sufficient to prevent mitochondrial dysfunction and the appearance of NCAM‐positive fibres in raptor k.o. muscles.

**Conclusions:** Our study shows that mTOR signalling in skeletal muscle fibres is critical for maintaining proper fibre innervation, preserving the NMJ structure in both the muscle fiber and the motor neuron. In addition, considering the beneficial effects of exercise in most pathologies affecting the NMJ, our findings suggest that part of these beneficial effects of exercise are through the well‐established activation of mTORC1 in skeletal muscle during and after exercise.

**6-08**

**A new mouse model for dissecting the role of TGF‐β signalling in skeletal muscle**

**Laetitia Mazelin** ^1^, Lola Lessard^†, 1^, Victoire Cardot^2^, Julien Gondin^1^, Colline Sanchez^1^, Nadège Zanou^3^, Véronique Chauvet^2^, Girard Emmanuelle^1^, Stéphanie Sentis^2^, Nicolas Place^3^, Vincent Jacquemond^1^, Laurent Bartholin^2^ and Laurent Schaeffer^1^

^1^ *Institut NeuroMyoGene (INMG), Université Lyon 1, CNRS UMR 5310, INSERMU 1217, Lyon, France;* ^2^ *Université de Lyon, Université Claude Bernard Lyon 1, INSERM 1052, CNRS 5286, Centre Léon Bérard, Centre de Recherche en Cancérologie de Lyon (CRCL), Lyon, France;* ^3^ *Institute of Sport Sciences, Quartier UNIL‐Centre, Faculty of Biology‐Medicine, University of Lausanne, Bâtiment Synathlon, Lausanne, Switzerland*

^†^These authors contributed equally to this work.

**Background:** Transforming growth factor β (TGF‐β) ligands, TGF‐βs, myostatin, GDF11, and activins are major negative regulators of skeletal muscle mass. Dysregulation of TGF‐β proteins and their associated signalling components is increasingly being implicated in muscle wasting associated with chronic diseases such as myopathies, cancer, heart failure, and aging. Myofibre atrophy results from a combination of increased protein degradation, predominantly involving the ubiquitin–proteasome pathway, and decreased protein synthesis. However, underlying molecular mechanisms involved in TGFβ‐mediated myofibre atrophy are not fully elucidated as well as impact of chronic TGF‐β dysregulation on muscle physiology (i.e. muscle energy metabolism, calcium homeostasis, regenerative capacities, and neurotransmission). Studies conducted so far mostly relied on loss‐of‐function approaches and ligand gain‐of‐function, resulting in complex intricated effects not restricted to muscle cells.

**Methods:** A conditional mice model was generated to specifically activate TGF‐β signalling in adult myofibres through inducible expression of a constitutively active TGF‐βI receptor, TGFβ‐RI‐CA (RCA). Consequences of dysregulated TGF‐β signalling on muscle pathophysiology were investigated in RCA muscles to explore the mechanisms involved. Transcriptomic analysis was performed to identify new molecular signatures of TGF‐β‐mediated muscle wasting.

**Results:** Conditional activation of TGF‐β signalling in adult myofibres rapidly resulted in significant muscle atrophy, reduced muscle force, skeletal muscle metabolic modifications, and disturbed calcium homeostasis. A progressive fibre type switch toward more oxidative fibres was observed in both glycolytic and oxidative muscles. Mechanistically, constitutively active TGF‐βI receptor promoted activation of Smad2/3 signalling in myofibres leading to target genes expression and an imbalance between muscle protein synthesis and protein degradation. Crosstalk on AKT/mTOR signalling pathway was observed. Identified molecular signatures were investigated.

**Conclusions:** This study validated a new unbiased muscle‐specific mice model to investigate impact of chronic activation of TGF‐β signalling on skeletal muscle physiology and uncover new potential therapeutic targets for muscle‐wasting disorders.

**6-09**

**Relationship between the severity spectrum of sarcopenia and osteoporosis in elderly men and women in Singapore**

**Ecosse L. Lamoureux** ^1,2^, Alfred T.L. Gan^1^, Ryan E.K. Man^1,2^, Pauline Soh^1^, Amudha Aravindhan^1^, Khairulnizar Azman^1^, Eva K. Fenwick^1,2^ and Preeti Gupta^1^

^1^ *Singapore Eye Research Institute and Singapore National Eye Centre, Singapore;* ^2^ *Duke‐NUS Medical School, Singapore*

**Introduction:** Sarcopenia may increase the risk of osteoporosis in older individuals, but evidence is equivocal in Asia. We examined the association between the severity spectrum of sarcopenia and osteoporosis in elderly Singaporeans.

**Methods:** We included individuals from The **P**opulat**ION** H**E**alth ProfilE in **E**lde**R**ly Singaporeans study (**PIONEER**), a nationally representative, population‐based study of Singaporean Chinese, Malays, and Indians aged ≥60 years. Participants underwent body composition and bone density (dual energy X‐ray absorptiometry), grip strength (hand dynamometer), and habitual 4 m‐walking speed assessments. Pre‐sarcopenia was defined as low appendicular lean mass (LALM; men <7 kg/m^2^, women <5.4 kg/m^2^); sarcopenia as LALM and low muscle strength (LMS; men <26 kg, women <18 kg) or slow walking speed (SWS; ≤0.8 m/s); and severe sarcopenia as LALM, LMS, and SWS. Osteoporosis was defined as a low bone mineral density (BMD; T‐score, ≤ − 2.5) at either the hip, femoral neck, or lumbar spine sites. Modified Poisson regression models were used to determine the cross‐sectional sarcopenia–osteoporosis relationship.

**Results:** Of the 491 included participants [mean age (SD): 73.2 (8.3); 50.7% women], 160 (32.6%); 116 (23.6%); 161 (32.8%); and 54 (11.0%) had no sarcopenia, pre‐sarcopenia, sarcopenia, and severe sarcopenia, respectively. Osteoporosis was present in 31 (19.4%) individuals without sarcopenia and in 133 (40.2%) individuals with any sarcopenia. In multivariable‐adjusted analyses, any sarcopenia was associated with twice the risk of osteoporosis [risk ratio (RR) (95% confidence interval): 2.02 (1.44–2.84) overall, and 2.58 (1.28–5.22), and 1.82 (1.24–2.69) in men and women, respectively]. The RR for osteoporosis increased with the severity of sarcopenia [1.92 (1.29–2.87), 2.06 (1.42–2.98), and 2.16 (1.37–3.41) for pre‐sarcopenia, sarcopenia, and severe sarcopenia, respectively; *P*‐trend <0.001]. Similar trends were observed in gender‐stratified analyses.

**Conclusions:** Sarcopenia is highly prevalent in Singapore, and its severity spectrum is independently associated with increased risk of osteoporosis. Strategies to prevent and delay the progression of sarcopenia are warranted.

**6-10**

**Effects of comprehensive geriatric intervention on muscle quantity, quality, and function in community‐dwelling older adults**

**Yuya Watanabe** ^1,2,3,4^, Yosuke Yamada^2,4^, Tsukasa Yoshida^2,4^, Keiichi Yokoyama^3^, Emi Yamagata^5^, Motoko Miyake^3^, Yasuko Yoshinaka^3^ and Misaka Kimura^2,3^

^1^ *Doshisha University;* ^2^ *Kyoto Prefectural University of Medicine;* ^3^ *Kyoto University of Advanced Science;* ^4^ *National Institutes of Biomedical Innovation, Health and Nutrition;* ^5^ *Doshisha Women's College of Liberal Arts, Japan*

**Introduction:** Muscle quality is related to sarcopenia and/or frailty in older individuals. Echo intensity (EI) on ultrasonography images of skeletal muscle is now believed to reflect muscle quality. We investigated the effects of a self‐monitoring comprehensive geriatric intervention programme (CGIP) on muscle quantity, quality, and strength in community‐dwelling older adults and compared the effects of a CGIP using weekly class‐styled (CS) sessions and a home‐based (HB) programme.

**Methods:** We randomly assigned the 526 participants to two groups (CS 251 and HB 275) based on their residential districts. We conducted a 12 week intervention programme consisting of low‐load resistance exercises, physical activity increments, oral function improvements, and a nutritional guide. We encouraged the participants to increase their daily steps to 2500 steps/day from the baseline) and to follow the programme. While the CS group attended 90 min weekly sessions and independently executed the programme on other days, the HB group did not attend the weekly sessions but received instructions on programme execution. Before and after the intervention, we evaluated muscle thickness (MT) and EI of the anterior compartment of the right thigh using ultrasonography and also measured the knee‐extension strength.

**Results:** We analysed 389 participants' data (CS 194; HB 195) and excluded data of 137 participants whose ultrasonography measurements were missing. MT and EI improved significantly in both groups (MT from 41.3 to 42.6 mm and 42.7 to 44.5 mm; and EI from 22.8 to 21.5 and 23.2 to 21.4, in the CS and HB groups, respectively). Knee‐extension strength also increased in both interventions.

**Conclusions:** Since the results of CGIP revealed that older adults' muscle quantity, quality, and functions improved, we expect this intervention to largely help in preventing sarcopenia and/or frailty in the older population.

**6-11**

**Muscle strength and physical function during and after hospitalization in older adults: a cohort study**

**Peter Hartley** ^1^, Roman Romero‐Ortuno^2^, Ian Wellwood^3^ and Christi Deaton^3^

^1^ *University of Cambridge, Cambridge, UK;* ^2^ *Discipline of Medical Gerontology, Trinity College Dublin, Mercer's Institute for Successful Ageing, St James's Hospital, Dublin, Ireland;* ^3^ *Department of Public Health and Primary Care, University of Cambridge, Cambridge, UK*

**Introduction:** The aim of this study was to investigate changes in knee‐extension strength and physical function in older adults during and after acute hospital admission.

**Methods:** A repeated measures cohort study, using a convenience sample of participants were recruited during the first 24 h of their admission. Measurements of knee‐extension strength and functional mobility were taken at recruitment, on day 7 of admission (or at discharge if earlier) and again at follow‐up 4–6 weeks post‐discharge. Self‐reported general functional ability was scored for 2 weeks before admission and at follow‐up. During the first 7 days of admission, daily measurements of muscle strength were taken.

**Results:** We recruited 70 participants, of which 65 (28 women) had at least one repeated measure in hospital. Median age was 84 years. Whilst in hospital, participants were active for less than 4% of the day. Knee‐extension strength reduced during hospitalization by approximately 11% (*P* < 0.001), but there was no change post‐hospitalization (*P* = 0.458). There was a reduction in general functional ability between 2 weeks before admission and follow‐up (*P* = <0.001). Functional mobility improved during hospitalization (*P* < 0.001), but there was no change post‐hospitalization (*P* = 0.508). A repeated‐measures mixed model including 292 in‐hospital observations from 62 participants showed that greater loss in knee‐extension strength during hospitalization was associated with increased sedentary time on days 2 to 7 of the study, and with increased frailty, higher baseline strength, and lower baseline inflammatory levels.

**Conclusions:** Knee‐extension strength deteriorated during hospitalization and showed no recovery at follow‐up. Although functional mobility improved during hospitalization, it did not continue to improve between discharge and follow‐up, despite general functional ability not having recovered to pre‐hospital levels. Greater loss of knee‐extension strength was associated with increased sedentary time, frailty, lower admission inflammatory levels, and higher baseline strength.

**6-12**

**Clinical determinants of osteoporosis in patients with chronic heart failure**

**Takanori Tsukada** ^1^, Satoshi Katano^2^, Toshiyuki Yano^3^, Suguru Honma^4^, Takuya Inoue^2^, Yuhei Takamura^2^, Katsuhiko Ohori^3,5^, Akiyoshi Hashimoto^3,6^, Masaki Katayose^7^ and Tetsuji Miura^3^

^1^ *Cardiac Rehabilitation Center, Social Welfare Corporation, Hokkaido Social Work Association Obihiro Hospital, Obihiro, Japan;* ^2^ *Division of Rehabilitation, Sapporo Medical University Hospital, Sapporo, Japan;* ^3^ *Department of Cardiovascular, Renal and Metabolic Medicine, Sapporo Medical University School of Medicine, Sapporo, Japan;* ^4^ *Department of Rehabilitation, Sapporo Cardiovascular Hospital, Sapporo, Japan;* ^5^ *Department of Cardiology, Hokkaido Cardiovascular Hospital, Sapporo, Japan;* ^6^ *Division of Health Care Administration and Management, Sapporo Medical University School of Medicine, Sapporo, Japan;* ^7^ *Second Department of Physical Therapy, Sapporo Medical University School of Health Sciences, Sapporo, Japan*

**Background:** The aim of this study was to determine factors that underlie association of chronic heart failure (CHF) with osteoporosis.

**Methods:** We retrospectively enrolled 266 consecutive patients [76 years (interquartile range, IQR 66–82 years); 40% female] who were admitted to our institute for diagnosis and treatment of CHF in this study. Patients received dual energy X‐ray absorptiometry scan to measure bone mineral density. Osteoporosis was diagnosed by a T‐score at the lumber spine or femoral neck being less than −2.5.

**Results:** The prevalence of osteoporosis was 35% in CHF patients. Urine *N*‐telopeptide of type I collagen, a bone resorption marker, was significantly higher in patients with osteoporosis than in those without osteoporosis [7.8 (IQR 3.6–16.2) vs. 4.2 (IQR 2.3–8.1) nmol BCE/mmol Cr]. Patients with osteoporosis was older [79 (IQR 73–85) vs. 73 (IQR 62–81) years] and more frequently female. Patients with osteoporosis had slower gait speed and lower levels of body mass index (BMI 21.2 ± 3.4 vs. 23.8 ± 3.8 kg/m^2^). Multivariate logistic regression analysis after stepwise forward selection showed that female sex [odds ratio (OR) 7.05; 95% confidential interval (CI) 3.51–14.1), lower BMI (OR 4.49; 95% CI 1.58–12.78), slower gait speed (OR 0.76; 95% CI 0.65–0.89), use of loop diuretics (OR 2.74; 95% CI 1.33–5.64) and sodium‐glucose cotransporter 2 (SGLT2) inhibitors (OR 5.00; 95% CI 1.02–24.5), and lack of use of dipeptidyl‐peptidase 4 (DPP4) inhibitors (OR 0.30; 95% CI 0.11–0.77) and direct oral anticoagulants (DOACs; OR 0.47; 95% CI 0.22–0.99) were independent predictors of osteoporosis.

**Conclusions:** The use of loop diuretics and SGLT2 inhibitors and lack of use of DPP4 inhibitors and DOACs, in addition to traditional risk factors (female sex, lower BMI, and lower physical function), are independently associated with osteoporosis in CHF patients.

**6-13**

**11β‐HSD1 mediates muscle atrophy induced by glucocorticoid therapy in chronic inflammatory disease**

**Justine Michelle Webster** ^1,2^, Chloe Fenton^1^, Gareth Lavery^1^, Rachel Jones^1^, Karim Raza^1^, Ramon Langen^2^ and Rowan Hardy^1^

^1^ *University of Birmingham, Birmingham, UK;* ^2^ *Maastricht University, Maastricht, The Netherlands*

**Background:** Therapeutic glucocorticoids (GCs) are used to treat chronic inflammatory disease. Despite their anti‐inflammatory efficacy, chronic exposure to GCs elicits undesirable side effects, including muscle atrophy. 11β‐Hydroxysteroid dehydrogenase 1 (11β‐HSD1) activates GCs within muscle, is induced by inflammation, and has previously shown to drive GC‐induced muscle wasting. We examined the role of 11β‐HSD1 in mediating muscle wasting in chronic inflammatory disease when treated with therapeutic GCs.

**Methods:** Muscle biopsies were taken from patients with osteoarthritis (OA) or rheumatoid arthritis (RA). Cortisol production in the muscle was measured using thin‐layer chromatography (TLC), and catabolic and inflammatory gene expression were assessed. Global 11β‐HSD1 knock out (KO) animals were crossed onto the TNF‐tg murine model of polyarthritis and received vehicle or corticosterone (100 μg/mL) over 3 weeks in drinking water. Muscles were histologically assessed, and anabolic, catabolic and inflammatory gene, and protein expression were examined by RT‐qPCR and western blot. WT and 11β‐HSD1/KO murine muscle cultures were exposed to TNF‐α, dehydrocorticosterone, or both. Catabolic and inflammatory gene expression was measured.

**Results:** Cortisol activation in muscle was increased in RA patients than OA patients, and correlated with serum CRP levels. Local inflammation (IL‐6 mRNA) was increased in RA compared to OA, correlated with 11β‐HSD1 and was accompanied by elevated *Mstn* and *FoxO1* expression. The myopathy previously described in TNF‐tg and TNF‐tg^11β‐HSD1KO^ mice (1, 2), was aggravated by therapeutic GCs in TNF‐tg mice based on reduced muscle weights and fibre size, while their TNF‐tg^11β‐HSD1KO^ counterparts were protected from muscle wasting. This was accompanied by an attenuated GC‐induced elevation of *FoxO1* and *Trim63* mRNA abundance, and alterations in FoxO1 and ribosomal S6 phosphorylation. These data were in part recapitulated in primary muscle cultures. Together, these data suggest that 11β‐HSD1 inhibition may protect against therapeutic GC‐induced muscle wasting in chronic inflammatory disease.

**References**

1. Hayward, MD *et al*., *BMC Physiol.* 2007;**7**(1):13.

2. Hardy, R *et al*., *J Pathol.* 2016;**240**(4):472‐83.

**6-14**

**Association between vitamin D level and muscle strength in patients undergoing haemodialysis**

**Seok Hui Kang** ^1^, Miyeun Han^2^, Su‐Hyun Kim^3^, Ran‐hui Cha^4^ and Jun Chul Kim^5^

^1^ *Division of Nephrology, Department of Internal Medicine, Yeungnam University Medical Center, Daegu, Republic of Korea;* ^2^ *Division of Nephrology, Department of Internal Medicine, Pusan National University Hospital, Pusan, Republic of Korea;* ^3^ *Division of Nephrology, Department of Internal Medicine, Chung‐Ang University Hospital, Seoul, Republic of Korea;* ^4^ *Division of Nephrology, Department of Internal Medicine, National Medical Center, Seoul, Republic of Korea;* ^5^ *Division of Nephrology, Department of Internal Medicine, CHA Gumi Medical Center, CHA University, Gumi, Gyeongsangbuk‐do, Republic of Korea*

**Introduction:** Considering conflicting results or heterogeneity in study design, further investigations are needed to identify the definite association between vitamin D level and muscle health. Our study aimed to address these issues and to evaluate the association between vitamin D level and muscle mass indices, strength, or physical performance through comprehensive measurements in patients undergoing haemodialysis.

**Methods:** This study was performed in a tertiary medical centre. We included patients undergoing haemodialysis with age ≥ 20 years. A total of 84 patients were enrolled. The patients were divided into tertiles based on the 25‐hydroxy (25‐OH) vitamin D level as follows: lowest tertile (Lowest T, *n* = 28), middle tertile (Middle T, *n* = 28), and highest tertile (Highest T, *n* = 28). We evaluated the association between the tertiles and clinical outcomes including nutritional status, muscle mass, muscle function, handgrip strength (HGS), physical performance, and health‐related quality of life scales (HRQoL).

**Results:** There were no significant differences in the muscle mass indices and nutritional markers according to tertiles of 25‐OH vitamin D level. However, 25‐OH vitamin D level as a continuous variable or the tertile of 25‐OH vitamin D level as a categorical variable was positively associated with HGS. Logistic and linear regression analyses showed a consistent superiority of the Highest T in HGS compared with the Lowest or Middle T. Although the statistical significance was weak, the scores of various physical performance tests and the HRQoL scales were the highest in the Highest T, among the three tertiles.

**Conclusions:** The present study demonstrated that serum vitamin D level is associated with HGS in patients undergoing haemodialysis regardless of muscle mass indices or nutritional status.

**6-15**

**Clinical significance of volume status in body composition and physical activity measurements in haemodialysis patients**

**Seok Hui Kang** ^1^, Miyeun Han^2^, Su‐Hyun Kim^3^, Ran‐hui Cha^4^ and Jun Chul Kim^5^

^1^ *Division of Nephrology, Department of Internal Medicine, Yeungnam University Medical Center, Daegu, Republic of Korea;* ^2^ *Division of Nephrology, Department of Internal Medicine, Pusan National University Hospital, Pusan, Republic of Korea;* ^3^ *Division of Nephrology, Department of Internal Medicine, Chung‐Ang University Hospital, Seoul, Republic of Korea;* ^4^ *Division of Nephrology, Department of Internal Medicine, National Medical Center, Seoul, Republic of Korea;* ^5^ *Division of Nephrology, Department of Internal Medicine, CHA Gumi Medical Center, CHA University, Gumi, Gyeongsangbuk‐do, Republic of Korea*

**Introduction:** Recent studies have shown that hypervolaemia is associated with malnutrition. The aim of the study was to evaluate the association between volume status and body composition or physical performance measurements.

**Methods:** A total of 84 patients were enrolled. The participants were divided into tertiles on the basis of oedema index: low, middle, and high tertiles. Serum albumin and hs‐CRP levels were measured. Thigh muscle area index (TMA/Ht^2^, cm^2^/m^2^) was measured using computed tomography. Extracellular and total body water, and phase angle were obtained from the bioimpedance analysis. The oedema index was defined as the ratio of extracellular water to total body water. Subjective global assessment (SGA), handgrip strength (HGS), and gait speed (GS), Short Physical Performance Battery (SPPB), sit‐to‐stand for 30 s (STS30), timed up and go (TUG), sit‐to‐stand test performed 5 times (STS5), and 6‐min walk (6‐MW) tests were also evaluated.

**Results:** The levels of oedema index in low, middle, or high tertile was 0.338 ± 0.010, 0.356 ± 0.003, and 0.370 ± 0.007, respectively. On univariate analysis, SGA score and phase angle in high tertile were lowest among the three groups. On multivariate analysis, TMA/Ht^2^ and phase angle in high tertile were lowest among the three groups. Inverse correlations were observed between oedema index and TMA/Ht^2^, SGA score, phase angle, HGS, GS, SPPB, STS30, or 6 MW. Positive correlations were observed between oedema index and STS5 or TUG. AUROC, sensitivity, and specificity for predicting low GS were 0.641%, 34.5%, and 89.7%, respectively (*P* = 0.025). Those for predicting low SPPB were 0.791%, 68.0%, and 79.7%, respectively (*P* < 0.001). Results from correlation and subgroup analyses showed similar trends.

**Conclusions:** The present study demonstrates that high volume status is associated with decrease in muscle mass and physical performance regardless of inflammatory or nutritional status.

**6-16**

**Association of growth differentiation factor 15, a novel cachexia marker, with body anthropometry in a prospective haemodialysis cohort**

**Amy S. You**, Kamyar Kalantar‐Zadeh, Yoko Narasaki, Alejandra Novoa, Rene Amel Peralta, Tracy Nakata, Frank Zaldivar, Danh V. Nguyen and Connie M. Rhee

*University of California Irvine, Orange, CA, USA*

**Introduction:** In cancer‐associated cachexia, Growth Differentiation Factor 15 (GDF15) has been identified as a novel appetite regulator that is associated with anorexia, weight loss, lower lean body and fat mass, and decreased survival when overexpressed. These findings bear particular relevance to the haemodialysis population, in whom weight loss and protein‐energy wasting are strong predictors of death. We thus sought to examine the relationship between circulating GDF15 levels and body anthropometry measurements in a prospective haemodialysis cohort.

**Methods:** Among 205 haemodialysis patients from the multicentre prospective ‘Malnutrition, Diet, and Racial Disparities in Chronic Kidney Disease’ (MADRAD) study, we examined cross‐sectional associations of serum GDF15 levels with body anthropometry measurements conducted over October 2011 to April 2012. Associations of GDF15 levels categorized as tertiles with lower levels of body mass index (BMI), mid‐arm circumference (MAC), mid‐arm muscle circumference (MAMC), biceps skinfold, triceps skinfold, near‐infrared body fat percentage (%), and waist circumference (defined as the lowest two tertiles of observed values; reference: highest tertile) were examined using case‐mix adjusted logistic regression models.

**Results:** In case‐mix analyses, patients with the highest tertile of GDF15 levels had significantly lower BMI, MAC, and NIR body fat % (ref: lowest GDF15 tertile): ORs (95% CIs): 2.90 (1.24–6.78), 2.51 (1.06–5.94), and 3.33 (1.10–10.1), respectively. There was also a trend between higher GDF15 levels and lower MAC, waist circumference, and biceps skinfold, although associations did not achieve statistical significance.

**Conclusions:** In haemodialysis patients, higher GDF15 levels were associated with lower levels of skeletal muscle, fat, and total body mass. Further studies are needed to determine whether reduction of GDF15 levels improves body composition and ameliorates protein–energy wasting in this population.

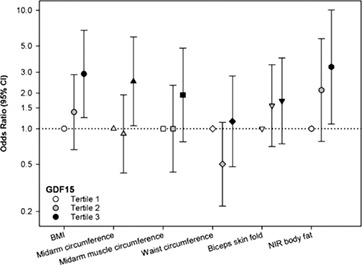

**6-17**

**Thyroid status and body composition in a prospective haemodialysis cohort**

**Yoko Narasaki** ^1^, Amy S. You^1^, Rachelle Bross^2^, Alejandra Novoa^1^, Yalitzi Guerrero^1^, Tracy Nakata^1^, Kamyar Kalantar‐Zadeh^1^ and Connie M. Rhee^1^

^1^ *University of California Irvine, Orange, CA, USA;* ^2^ *Los Angeles Biomedical Research Institute, Torrance, CA, USA*

**Introduction:** Thyroid dysfunction is a highly prevalent metabolic disorder in haemodialysis (HD) patients. In the general population, hypothyroidism is associated with increased body weight due to reductions in energy expenditure. However, little is known about the impact of thyroid status on body composition in end‐stage renal disease patients. We thus sought to examine the relationship between serum thyrotropin (TSH) levels, the most sensitive and specific single biochemical metric of thyroid function, with body composition measured by dual‐energy X‐ray absorptiometry (DXA) scan in HD patients.

**Methods:** Among 103 patients from the Anti‐Inflammatory and Anti‐Oxidative Nutrition in Hypoalbuminemic Dialysis Patients (AIONID) trial, we examined cross‐sectional associations of TSH [categorized as the highest quartile (Q4) versus lowest three quartiles (Q1–3)] with DXA‐ascertained body composition parameters (i.e. fat, bone, and lean mass). Using logistic regression models, we estimated associations between TSH with higher levels of fat, muscle, and bone [defined as the highest three quartiles (Q2–4) versus lowest quartile (Q1)] in analyses adjusted for case‐mix and expanded case‐mix covariates.

**Results:** In case‐mix analyses, the highest quartile of TSH levels was associated with higher levels of whole‐body total fat mass, subtotal fat mass, whole‐body total fat mass index, and whole‐body total mineral content (ref: serum TSH Q1–3): ORs (95% CIs): 8.27 (1.54–44.5), 8.27 (1.54–44.5), 4.67 (1.07–20.3), and 5.78 (1.29–25.9), respectively. While there was a trend toward higher TSH levels and higher levels of lean mass parameters, associations did not achieve statistical significance. A similar pattern of findings was observed in expanded case‐mix analyses.

**Conclusions:** In HD patients, higher TSH levels were associated with increased fat mass and bone mineral content. Further studies are needed to determine whether thyroid‐related alterations in body composition impact the health and survival of HD patients and whether reduction of TSH with treatment influences muscle and bone parameters in this population.

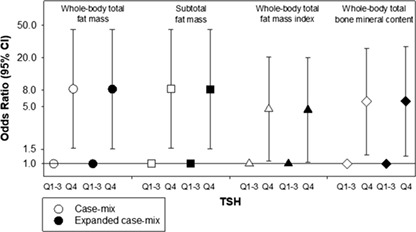

**6-18**

**Skeletal muscle index and tumour aggressiveness in clear cell renal cell carcinoma**

Alejandro Sanchez^1^, Stacey Petruzella^2^, Marguerite Samson^2^, Oguz Akin^3^, Mike Paris^4^, Marina Mourtzakis^4^, Ari Hakimi^1^, Paul Russo^1^ and **Helena Furberg** ^2^

^1^ *Department of Urology, Memorial Sloan Kettering Cancer Center, New York, NY, USA;* ^2^ *Department of Epidemiology & Biostatistics, Memorial Sloan Kettering Cancer Center, New York, NY, USA;* ^3^ *Body Imaging Service, Department of Radiology, Memorial Sloan Kettering Cancer Center, New York, NY, USA;* ^4^ *Department of Kinesiology, University of Waterloo, ON, Canada*

**Introduction:** Clear cell renal cell cancer (ccRCC) patients who have low skeletal muscle index (SMI) at the time of nephrectomy experience worse cancer‐specific and overall survival than patients with high SMI, but potential mechanisms underlying this association are unknown. ccRCC is a heterogeneous disease that can be classified into two molecular subtypes (ccA and ccB) based on gene expression patterns. ccB tumour subtype is considered more aggressive and has a significantly worse prognosis than ccA subtype. We examined how SMI at the time of nephrectomy was associated with molecular tumour subtype while considering age, sex, and stage at diagnosis.

**Methods:** The study cohort consisted of 76 incident ccRCC patients treated by nephrectomy whose tumours were transcriptomically profiled by the Cancer Genome Atlas. The ClearCode34 gene expression classifier categorized patients into either ccA or ccB molecular subtypes. Computerized tomography scans without contrast performed within 60 days of surgery were reviewed using Slice‐O‐Matic software to determine SMI (skeletal muscle cross‐sectional area divided by height in meters squared). SMI was classified into high versus low using the gender‐specific cut points of <55 cm^2^/m^2^ for men and <39 cm^2^/m^2^ for women. Odds ratios and 95% confidence intervals (OR 95% CI) describe associations between SMI and molecular subtype in logistic regression models that adjust for age, sex, BMI, and stage. A subgroup analysis restricted to patients with early stage disease (*n* = 47) was performed to address the possibility of muscle wasting due to advanced stage disease. Statistical significance was regarded as *P*‐value < 0.05.

**Results:** Patients were predominantly male (77%) and had early stage disease (62%). Median age was 59 years (IQR: 51–68). Overall, 40% of patients were classified as having low SMI and 42% of tumours were ccB subtype. Patients with low SMI were significantly more likely to have the aggressive ccB tumour subtype (66%) than patients with high SMI (26%); adjusted OR 9.1 (95% CI: 2.1–40.1). This pattern persisted even when analyses were restricted to patients with early stage disease (*P*‐value = 0.01).

**Conclusions:** While preliminary, our findings suggest that patients with low SMI harbour more aggressive ccB tumours and lend biologic support to the observation that low SMI is associated with poor prognosis. Given the cross‐sectional nature of this analysis, it is not clear whether low SMI is a cause or consequence of tumour aggressiveness, or whether addressing low SMI could improve clinical outcomes among ccRCC patients.

**Acknowledgement:** Chanel grant (H. F.) and Ruth L. Kirschstein Research Service Award T32CA082088 (A. S.).

**6-19**

**Systemic low‐grade inflammation and its association with muscle mass throughout the age‐span—Copenhagen Sarcopenia Study**

**Rikke S. Kamper** ^1,2^, Julian Alcazar^1,3^, M. Gudmundsson^2^, Lars L. Andersen^5^, Bryan Haddock^2^, R. Niklas, Peter Hovind^1,2^ and S.K. Hansen^1^ and Charlotte Suetta^1,2,4^

^1^ *Bispebjerg & Frederiksberg Hospital, Copenhagen, Denmark;* ^2^ *Rigshospitalet Glostrup, Copenhagen, Denmark;* ^3^ *GENUD Toledo Research Group, Universidad de Castilla‐La Mancha, Toledo, Spain;* ^4^ *Herlev & Gentofte Hospital, Copenhagen, Denmark;* ^5^ *The National Research Centre for the Working Environment (NFA), Copenhagen, Denmark*

**Introduction:** Chronic low‐grade inflammation has been associated with sarcopenia [1,2]. However, results regarding the role of systemic low‐grade inflammation on muscle mass are inconsistent, and lack of unequivocal evidence as to whether systemic levels of inflammatory markers could represent biomarkers of sarcopenia. Thus, the aim of this study was to investigate the association between circulating inflammatory biomarkers and appendicular lean mass (ALM/h^2^) throughout the age‐span.

**Methods:** 1177 healthy men and women (range: 22–93 years) were included in the present study. ALM/h^2^ (kg/m^2^) was assessed by DEXA (iDXA, GE Lunar). Blood samples were analysed for IL‐1β, IL‐4, IL‐6, IL‐13, IFN‐γ, and TNF‐α using multiplex bead‐based immunoassays (Bio‐Rad) and for hsCRP using latex particle‐enhanced immunoturbidimetric assays following the manufacturer's instructions (Roche Diagnostics). Biomarkers were log‐transformed, and analyses were stratified by gender and adjusted for age, visceral fat, and assay plate ID.

**Results:** There was an inverse association between hsCRP and ALM/h^2^ in young men (22–39 years), but no other associations were found in subjects under 80 years. In subjects over 80 years, IL‐13, IL‐4, IFN‐γ, and TNF‐α were inversely associated with ALM/h^2^ in men. Women over 60 years with high (>0.71 pg/mL) or intermediate (0.28–0.71 pg/mL) levels of IL‐13 had more than two‐fold greater odds of having lower ALM/h^2^ compared to women with low levels (<0.28 pg/mL).

**Conclusions:** The present study demonstrated an association of low‐grade inflammation with aging in apparently healthy men and women. Except for an inverse association between hsCRP and ALM/h^2^ in young men, there was no association between ALM/h^2^ and inflammatory biomarkers in healthy subjects under the age of 80 years. Of note, an inverse association of both proinflammatory and anti‐inflammatory markers with ALM/h^2^ was observed in men over 80 years. In contrast, increased levels of IL‐13 were associated with having lower ALM/h^2^ in women over 60 years.

**References**

1. Bano, G., Trevisan, C., Carraro, S., et al. (2017). Inflammation and sarcopenia: a systematic review and meta‐analysis. *Maturitas*, **96**, 10–15.

2. Visser, M., Pahor, M., Taaffe, D. R., et al. (2002). Relationship of interleukin‐6 and tumor necrosis factor‐α with muscle mass and muscle strength in elderly men and women: The Health ABC Study. *The Journals of Gerontology: Series A*, **57**(5), M326–M332.

**7-01**

**13 weeks of supplementation of vitamin D and leucine‐enriched whey protein nutritional supplement attenuates chronic low grade inflammation in sarcopenic older adults: the PROVIDE study**

**Ivan Bautmans** ^1^, Rose Njemini^1^, Yvette Luiking^2^, Louis N. Forti^1^, Sjors Verlaan^3^, Jürgen M. Bauer^4^, Robert Memelink^1,5^, Kirsten Brandt^6^, Lorenzo M. Donini^7^, Marcello Maggio^8^, Tony Mets^1^, Sander L.J. Wijers^2^, Cornel Sieber^9^, Tommy Cederholm^10^ and Keliane Liberman^1^

^1^ *Frailty in Ageing research group (FRIA), Vrije Universiteit Brussel (VUB), Brussels, Belgium;* ^2^ *Nutricia Research, Nutricia Advanced Medical Nutrition, Utrecht, The Netherlands;* ^3^ *Department of Internal Medicine, Section of Gerontology and Geriatrics, VU University Medical Center, Amsterdam, The Netherlands;* ^4^ *Center of Geriatric Medicine, Heidelberg University, Heidelberg, Germany;* ^5^ *Faculty of Sports and Nutrition, Amsterdam University of Applied Sciences, Amsterdam, The Netherlands;* ^6^ *Human Nutrition Research Centre, Institute of Cellular Medicine, Newcastle University, Newcastle upon Tyne, UK;* ^7^ *Department of Experimental Medicine, Section of Medical Pathophysiology, Endocrinology and Human Nutrition, “Sapienza” University of Rome, Italy;* ^8^ *Department of Medicine and Surgery, Section of Geriatrics, University of Parma, Italy;* ^9^ *Friedrich‐Alexander‐Universität Erlangen‐, Nürnberg, Germany;* ^10^ *Department of Public Health and Caring Sciences/Clinical Nutrition and Metabolism, Department of Geriatric Medicine, Uppsala University Hospital, Sweden*

**Introduction:** A chronic low‐grade inflammatory profile (CLIP) is associated with sarcopenia in older adults. Protein and vitamin (Vit)D have immune‐modulatory potential, but evidence for effects of nutritional supplementation on CLIP is limited. The aim was to investigate whether 13 weeks of nutritional supplementation of VitD and leucine‐enriched whey protein affected CLIP in subjects enrolled in the PROVIDE‐study, as a secondary analysis.

**Methods:** Sarcopenic adults (low skeletal muscle mass) aged ≥65 years with mobility limitations (Short Physical Performance Battery 4‐9) and a body mass index of 20–30 kg/m^2^ were randomly allocated to two daily servings of active (*n* = 137, including 20 g of whey protein, 3 g of leucine and 800 IU VitD) or isocaloric control product (*n* = 151) for a double‐blind period of 13 weeks. At baseline and after 13 weeks, circulating interleukin (IL)‐8, IL‐1 receptor antagonist (RA), soluble tumour‐necrosis‐factor receptor (sTNFR)1, IL‐6, high‐sensitivity C‐reactive protein, pre‐albumin, and 25‐hydroxyvitamin (OH)D were measured. Data analysis included repeated measures analysis of covariance (corrected for dietary VitD‐intake) and linear regression.

**Results:** IL‐6 and IL‐1RA serum levels showed overall increases after 13 weeks (*P* = 0.006 and *P* < 0.001, respectively). For IL‐6 a significant time*treatment interaction (*P* = 0.046) was observed, with no significant change over time in the active group (*P* = 0.155) compared to control (significant increase *P* = 0.012). IL‐8 showed an overall significant decrease (*P* = 0.03). The change in pre‐albumin was a significant predictor for changes in IL‐6 after 13 weeks.

**Conclusions:** We conclude that 13 weeks of nutritional supplementation with VitD and leucine‐enriched whey protein may attenuate the progression of CLIP in older sarcopenic persons with mobility limitations.

**7-02**

**Intramuscular fat is predictive of hypoglycaemia incidence: results from the MENU study**

**Eyal Leibovitz** ^1^, Tomer Perluk^2^ and Mordechai Shimonov^2,3^

^1^ *Department of Internal Medicine “A”, Yoseftal Hospital, Eilat;* ^2^ *Department of Surgery “A”, Wolfson Medical Center, Holon;* ^3^ *Sackler School of Medicine, Tel Aviv University, Tel Aviv, Israel*

**Background:** To analyse body composition among non‐critically ill patients with high risk of malnutrition, with and without hypoglycaemia.

**Methods:** Included were patients enrolled in the MENU study that underwent CT scanning during their hospitalization. The NRS2002 was used for nutritional screening. Body composition was analysed at the level of L3 using Slice‐O‐matic software (TomoVision, Montreal, Canada). Patients were categorized as hypoglycaemic if they had at least one documented hypoglycaemic event during the hospitalization period (glucose ≤70 mg/dL). Regression analysis was used to examine the association of body composition with incident hypoglycaemia.

**Results:** Included were 155 patients (mean age 69.7 ± 15.7, 51.6% were men, 52.9% had diabetes mellitus). Rate of positive NRS2002 was 57.8%, and 26 patients (16.7%) had at least one documented hypoglycaemic event. Patients at risk of malnutrition had lower muscle mass (44.9 ± 12.5 vs. 49.3 ± 13.6 cm^2^/m^2^, *P* = 0.045) and lower subcutaneous fat (59.4 ± 40.7 vs. 86.8 ± 49.7 cm^2^/m^2^, *P* < 0.001) and visceral fat (64.3 ± 40.2 vs. 93.4 ± 58.1 cm^2^/m^2^, *P* = 0.001). Regression analysis showed that the NRS2002 (OR 4.986, 95% CI 1.052–23.632, *P* = 0.043) and insulin treatment (OR 7.769, 95% CI 1.529–39.461, *P* = 0.013) were predictive of hypoglycaemia in this patient population. Furthermore, intramuscular fat was also indicative of hypoglycaemia incidence (OR 1.091, 95% CI 1.002–1.187, *P* = 0.044). Muscle mass, subcutaneous, and visceral fat mass, as well as sex, albumin, and diabetes mellitus status did not affect incident hypoglycaemia.

**Conclusions:** Our data suggest that intramuscular fat is predictive of hypoglycaemia incidence among patients admitted to internal medicine units, irrespective of malnutrition risk.

**7-03**

**Changes in metabolic, nutritional, and hormonal profile among subjects professionally exposed to urban pollution in a high intensity traffic area**

**Alessio Molfino** ^1^, Maurizio Muscaritoli^1^, Annunziata Germano^3^, Cesarina Ramaccini^1^, Rossella Alfano^3^, Alessandra Spagnoli^4^, Maria Triassi^3^, Maria Ida Amabile^2^ and Silvana Fiorito^5^

^1^ *Department of Translational and Precision Medicine, Sapienza University of Rome, Rome, Italy;* ^2^ *Department of Surgical Sciences, Sapienza University of Rome, Rome, Italy;* ^3^ *Department of Public Health, University Federico II, Naples, Italy;* ^4^ *Department of Public Health and Infectious Diseases, Sapienza University of Rome, Rome, Italy;* ^5^ *Institute of Translational Pharmacology, CNR‐Rome, Italy*

**Introduction:** Evidences showed that exposure to diesel exhaust particles is associated with cardiopulmonary, vascular, and oncologic diseases. Recent data from large cohorts of subjects have highlighted associations between exposure to ambient air particulate matter (PM)_2.5_ pollution and increased cardiovascular morbidity and mortality, as well as increased risk for obesity and diabetes. In this light, we aimed to identify association(s) between nutritional, metabolic, and hormonal derangements and exposure to environmental pollution in an Italian population of traffic policemen professionally exposed to high levels of air PM.

**Methods:** We performed a cross‐sectional study considering adult male municipal policemen of the city of Naples, Italy, professionally exposed to airborne nanoparticles in an urban area at high traffic density (Exposed group) compared to non‐exposed municipal policemen (at least 1 year indoor working) (Non‐exposed group) matched by age and body mass index (BMI). Clinical and laboratory assessments were performed, including serum levels of leptin, adiponectin, and ghrelin by ELISA.

**Results:** A total of 199 participants were consecutively enrolled, 100 adult males in the Exposed‐group and 99 adult males in the Non‐exposed group. No differences were observed between the two groups in terms of body weight, BMI, and lipid profile, whereas plasma glucose levels and HOMA‐IR were higher in the Non‐exposed group compared to the Exposed one (*P* = 0.009 and *P* = 0.03, respectively). Metabolic syndrome was documented in 32% of the Exposed group and in the 52.5% of the Non‐exposed group (*P* = 0.008); no differences were seen between the two groups in terms of adiponectin, ghrelin, and leptin serum levels. In the Exposed group, we found a negative correlation between BMI and serum adiponectin levels (*P* = 0.04) and a positive correlation between BMI and serum leptin concentrations (*P* < 0.0001), whereas in the Non‐exposed group, we found only a positive correlation between BMI and serum leptin (*P* < 0.0001). Subjects in the Exposed‐group with metabolic syndrome showed lower serum adiponectin levels and higher serum leptin levels with respect to those without metabolic syndrome (*P* < 0.0001 and *P* = 0.002, respectively), while in subjects with metabolic syndrome within Non‐exposed group, we found higher serum leptin levels when compared to those without metabolic syndrome (*P* = 0.58 and *P* = 0.01; respectively). Stratifying participants for the presence of metabolic syndrome, when comparing the two groups, we found lower serum adiponectin levels in the Exposed group with respect to the Non‐exposed (*P* = 0.007). Finally, when comparing the two groups, after stratifying participants for HOMA‐IR >2.5, we found lower adiponectin serum levels in the Exposed group with respect to the Non‐exposed (*P* = 0.038).

**Conclusions:** Our results seem to suggest that exposure to air PM pollution is associated with the presence of markers of insulin resistance and metabolic syndrome, likely due to low adiponectin circulating levels, determining impaired protective function against systemic inflammation.

**7-04**

**Association of growth differentiation factor 15 and malnutrition inflammation score in a prospective haemodialysis cohort**

**Amy S. You**, Kamyar Kalantar‐Zadeh, Yoko Narasaki, Alejandra Novoa, Rene Amel Peralta, Tracy Nakata, Frank Zaldivar, Danh V. Nguyen and Connie M. Rhee

*University of California Irvine, Orange, CA, USA*

**Introduction:** Growth differentiation factor 15 (GDF15) is a protein in the transforming growth factor‐β family that acts directly upon the hypothalamus to reduce food intake and energy expenditure. In animal models and clinical studies of cancer‐associated cachexia, higher GDF15 levels have been associated with satiety, weight loss, and death. While weight loss and protein‐energy wasting (PEW) are also known to be strong predictors of mortality in haemodialysis patients, little is known about the relationship between GDF15 levels and nutritional status in this population.

**Methods:** Among the 205 haemodialysis patients from the multicentre prospective ‘Malnutrition, Diet, and Racial Disparities in Chronic Kidney Disease’ (MADRAD) study, we examined cross‐sectional associations of serum GDF15 levels measured over October 2011 to April 2012 with results from their Malnutrition Inflammation Score (MIS) instruments, a validated and quantitative nutritional assessment tool used to estimate PEW in kidney disease patients (i.e. higher levels indicate worse levels of PEW). Associations of GDF15 levels categorized as tertiles with higher MIS levels (defined as >median of observed values; reference: ≤median) were estimated using case‐mix adjusted logistic regression models. In secondary analyses, we examined GDF15 defined as (i) >median versus ≤median and (ii) categorized as quartiles.

**Results:** In case‐mix analyses, patients with incrementally higher GDF15 levels had greater likelihood of higher MIS levels (ref: lowest GDF15 tertile): ORs (95% CIs): 2.70 (1.21–6.03) and 2.87 (1.22–6.74) for the first and second GDF15 tertiles, respectively. In secondary analyses, higher GDF15 levels defined as >median and the highest GDF15 quartile were also associated with higher MIS levels: 2.27 (1.14–4.55) and 3.89 (1.40–10.8), respectively.

**Conclusions:** In a prospective haemodialysis cohort, higher GDF15 levels were associated with poorer nutritional status defined by MIS level. Further studies are needed to determine whether GDF15 may be a novel target for PEW in the haemodialysis population.

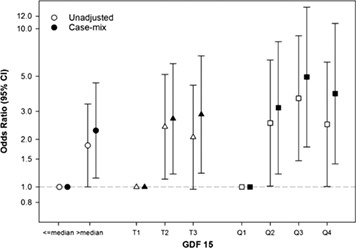

**7-05**

**Dietary fibre intake and mortality risk in a prospective haemodialysis cohort**

**Yoko Narasaki** ^1^, Amy S. You^1^, Linda W. Moore^2^, Sara Colman^3^, Alejandra Novoa^1^, Rene Peralta^1^, Tracy Nakata^1^, Danh V. Nguyen^1^, Kamyar Kalantar‐Zadeh^1^ and Connie M. Rhee^1^

^1^ *University of California Irvine, Orange, CA, USA;* ^2^ *Houston Methodist Hospital, Houston, TX, USA;* ^3^ *DaVita Inc., El Segundo, CA, USA*

**Introduction:** In the general population, epidemiologic data suggest that higher dietary fibre intake is associated with lower mortality, presumably due to favourable effects on nutritional and metabolic health, including decreased glucose absorption, lowering of cholesterol, gut microbiome‐induced production of immunomodulatory and anti‐inflammatory short fatty chain acids, reductions in blood pressure, and trapping of carcinogens. Haemodialysis patients are subject to a number of dietary restrictions (i.e. dietary potassium restriction), which may subsequently limit their fibre intake. We thus sought to examine the relationship between dietary fibre intake and mortality in a prospective cohort of haemodialysis patients.

**Methods:** Among the 415 haemodialysis patients from the multicentre prospective ‘Malnutrition, Diet, and Racial Disparities in Chronic Kidney Disease’ study, information regarding dietary fibre intake was obtained using the Block Food Frequency Questionnaire administered over October 2011–March 2015, with follow‐up through 2019. Associations of baseline dietary fibre intake categorized as tertiles with all‐cause death risk were examined using unadjusted, case‐mix, expanded case‐mix, expanded case‐mix + laboratory, and expanded case‐mix + laboratory + nutrition adjusted Cox models.

**Results:** The mean ± SD age of the cohort was 56 ± 15 years, among whom 45% were female, and 36% and 48% were African‐American and Hispanic, respectively. Across all levels of adjustment, patients with the lowest tertile of dietary fibre intake had higher mortality risk (ref: highest tertile): HRs (95% CIs) 1.77 (1.18, 2.67), 1.80 (1.18, 2.73), 1.86 (1.21, 2.86), 2.19 (1.39, 3.44), and 2.73 (1.47, 5.06) in unadjusted, case‐mix, expanded case‐mix, expanded case‐mix + laboratory, and expanded case‐mix + laboratory + nutrition models, respectively.

**Conclusions:** In a diverse prospective cohort of haemodialysis patients, there was a robust association between lower dietary fibre intake and higher all‐cause death risk. Further studies are needed to determine underlying mechanisms and to define the optimal amount of dietary fibre intake in this population.

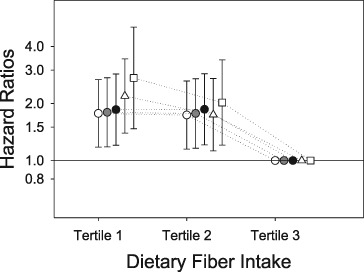

**7-06**

**Effects of β‐hydroxy‐β‐methylbutyrate supplementation in patients with liver cirrhosis: a randomized controlled pilot study**

**Angelo Bruni**, Barbara Lattanzi, Nicoletta Fabrini, Maurizio Muscaritoli, Alessio Molfino, Simone Di Cola, Alessandra Pigliacelli and Manuela Merli

*Dipartimento di Medicina Translazionale e di Precisione, Policlinico Umberto I, La Sapienza, Rome, Italy*

**Introduction:** Frailty is defined as a syndrome of physiological decline in late life. In patients with cirrhosis the dysregulation of catabolic and anabolic muscular pathways cause, an increased risk to anticipate sarcopenia and frailty. We aimed to evaluate 3 g/die β‐hydroxy‐β‐methylbutyrate (HMB) supplementation for 12 weeks (T1), on muscular mass and performance in patients with cirrhosis

**Methods**: For each patient, at the beginning of the study (T0), clinical history and blood parameters were collected; cognitive tests (MMSE, PHES, and ANT) and physical test (6MWT, FCS, and HGT) anthropometric measurements using ‘quadriceps femoris ultrasound’ and bioimpedentiometry and Liver Frailty Index (LFI) were also performed. Patients were randomized into a treatment group (3 g per die HMB supplementation for 12 weeks) and a control group. (3 g per die sorbitol supplementation for 12 weeks). Both groups received nutritional counselling and indications on physical activity to carry out during treatment period. At the end of the 12 weeks (T1), all the data collected at T0 were repeated and compared in each patient.

**Results:** At present, 22 cirrhotic patients were enrolled, and preliminary results are shown. Patients receiving HMB showed a statistically significant improvement in muscular performance at FCS ranging from 14.5 s (±5.4 DS) to 11.6 s (±2.7 DS), at 6MWT rising from 346.8 m (±66 DS) to 416 m (±57 DS) and LFI, decreasing from 4.0 (±0.4 DS) to 3.7 (±0.4 DS) with *P* value = 0.008. No significant variation, between T0 and T1, were reported in patients assigned to the control group.

**Conclusions:** The preliminary results of this controlled randomized study suggests efficacy of HMB supplementation in enhancing muscular performance and, consequently, reducing frailty in cirrhotic patients.

**7-07**

**Nutritional status affects the risk of contrast induced nephropathy after percutaneous coronary intervention**

**Miyeun Han** ^1^, Seok Hui Kang^2^, Jun Chul Kim^3^, Su‐Hyun Kim^4^, Ran‐hui Cha^5^, Eun Young Seong^1^, Hyewon Lee^6^ and Sang Heon Song^1^

^1^ *Division of Nephrology, Department of Internal Medicine, Pusan National University Hospital, Pusan, Republic of Korea;* ^2^ *Division of Nephrology, Department of Internal Medicine, Yeungnam University Hospital, Daegu, Republic of Korea;* ^3^ *Division of Nephrology, Department of Internal Medicine, CHA Gumi Medical Center, CHA University, Gumi, Gyeongsangbuk‐do, Republic of Korea;* ^4^ *Su‐Hyun Kim, Department of Internal Medicine, Chung‐Ang University Hospital, Seoul, Republic of Korea;* ^5^ *Ran‐hui Cha, Department of Internal Medicine, National Medical Center, Seoul, Republic of Korea;* ^6^ *Division of Cardiology, Department of Internal Medicine, Pusan National University Hospital, Pusan, Republic of Korea*

**Introduction:** Contrast induced nephropathy (CIN) is the major common cause of hospital acquired acute kidney injury and associated with longer hospital stay, increased morbidity, and mortality. Various risk factors for CIN after coronary artery angiography are known; however, the risk of malnutrition is not validated well. The aim of this study is to evaluate the effect of nutrition for CIN after coronary intervention.

**Method:** A total of 1913 subjects who got percutaneous coronary intervention (PCI) in Pusan National University hospital from January 2014 to November 2018 were included in this study. The subjects who already got haemodialysis, had eGFR under 15 mL/min/1.73 m^2^, or had no baseline creatinine were excluded. CIN is defined as an elevation of serum creatinine (Scr) of more than 50% or ≥0.3 mg/dL from baseline within 48 h. Patients with a serum albumin less than 3.5 g/dL and/or a TLC less than 1500 cells per mm^3^ were classified as having protein energy malnutrition (PEM).

**Results:** 95 (4.9%) developed CIN after PCI. The subjects with CIN were older, had higher proportion of female, diabetes, and hypertension. The level of haemoglobin, total lymphocyte count, albumin, estimated glomerular filtration rate was lower in CIN group, whereas the level of creatinine and C‐reactive protein was higher in CIN group. In multivariate logistic regression analysis for CIN, body mass index, heart rate, albumin, estimated glomerular filtration rate, proteinuria, and contrast volume were significant related. When we combine serum albumin level and TLC for PEM, PEM was still significant risk factors in multivariate logistic regression analysis (HR 3.947, 95% CI 1.522, 10.235, *P* = 0.005).

**Conclusions:** PEM raised the risk of CIN after coronary intervention. Clinicians should be aware of nutritional status and increased risk of CIN.

**7-08**

**Assessment of sarcopenia and malnutrition in patients with head and neck cancer undergoing treatment of curative intent—implications for practice**

Teresa Brown^1,2^, Merrilyn Banks^2^, Louise Campbell^2^, Brett Hughes^1,2^, Elizabeth Ahern^2^, Charles Lin^1,2^, Liz Kenny^1,2^ and **Judy Bauer** ^1^

^1^ *The University of Queensland, Australia;* ^2^ *Royal Brisbane and Women's Hospital, Brisbane, Australia*

**Background:** Malnutrition and sarcopenia are poor prognostic factors for survival in patients with head and neck cancer (HNC). Assessments of malnutrition and sarcopenia are important factors to consider in the management of these patients. To determine the prevalence of CT‐defined sarcopenia and malnutrition in patients with HNC undergoing radiotherapy ± other treatment modality of curative intent.

**Methods:** Nutritional status and body composition of patients with HNC undergoing radiotherapy of curative intent ± other treatment modality participating in a RCT were included. Outcomes were measured at baseline and 3 months post‐treatment. Malnutrition was assessed using the Patient Generated Subjective Global Assessment (PG‐SGA). Sarcopenia was derived from PET‐CT scan L3 tissue density data (Slice‐O‐Matic software, Version 5.0, TomoVision) and defined using threshold values for skeletal muscle index, stratified by BMI and gender.

**Results**: Of the 108 patients [90% male; 60.7 years (SD 10.2); stage III 11%, IV 89%, BMI 27.9 kg/m^2^ (SD 5.3); 86 well nourished], 99 had available baseline CT images and 89 at follow‐up. At baseline, 54% (53/99) had sarcopenia (44 well‐nourished: 9 moderately malnourished). There was no difference in sarcopenia across BMI categories: 67% underweight, 54% healthy, 58% overweight, and 45% obese; *P* = 0.688). At 3 months post‐treatment, 65% (58/89) had sarcopenia (48 well‐nourished, 9 moderately malnourished). Overall mean (SD) weight change was −11.4 (6.0)% with no difference based on sarcopenia at baseline (−10.9% sarcopenic vs. −12.0% not sarcopenic, *P* = 0.365). Mean (SD) change in skeletal muscle index was −12.0% (8.8) and total fat −31.7% (17.4).

**Conclusions:** There is a high prevalence of sarcopenia in patients with HNC undergoing treatment of curative intent that is not adequately detected by current methods of nutrition assessment. Implementation of assessment of sarcopenia prior to treatment and assessment of body composition change during treatment is warranted.

**Conflict of interest:** There is no conflict of interest to declare.

**8-01**

**Exercise and dietary switch differentially resolve NASH in mice with sarcopenic obesity**

**Anita M. van den Hoek** ^1^, Lars Verschuren^2^, Martien P.M. Caspers^2^, Dide A.O. Reijmer^2^, Nicole Worms^1^, Marijke Voskuilen^1^, Aswin L. Menke^1^ and Robert Kleemann^1^

^1^ *Department of Metabolic Health Research, The Netherlands Organization for Applied Scientific Research (TNO), Leiden, The Netherlands;* ^2^ *Department of Microbiology and Systems Biology, The Netherlands Organization for Applied Scientific Research (TNO), Zeist, The Netherlands*

**Introduction:** Age‐related abdominal fat mass accumulation and muscle weakness have been associated with several adverse health effects, including non‐alcoholic steatohepatitis (NASH). Lifestyle recommendations remain the primary treatment for NASH patients, although the specific effects of exercise and dietary changes on NASH and underlying molecular pathways involved remain elusive. We therefore investigated the effects of exercise, dietary change, and the combination thereof on already manifest NASH in mice with sarcopenic obesity.

**Methods:** Ldlr^−/−^.Leiden mice received a high fat diet for 30 weeks to induce NASH. After 30 week mice were left untreated (control group) or received a running wheel, were switched to an isocaloric healthy chow diet, or the combination thereof, for another 20 weeks. Effects on body composition, grip strength, plasma and liver biochemical variables, and liver histology were assessed.

**Results:** Both exercise and dietary change significantly reduced body weight and fat mass, improved grip strength, and the combination treatment had additive effects. While exercise significantly reduced micro‐vesicular steatosis, dietary change significantly reduced macro‐vesicular steatosis. The combination treatment revealed additive effects for both macro‐vesicular and micro‐vesicular steatosis. Hepatic inflammation was almost fully reduced by each monotreatment. Hepatic fibrosis was significantly reduced by dietary switch, tended to be reduced by exercise, and combined treatment had no additive resolving effect.

**Conclusions:** Both exercise and dietary lifestyle interventions showed beneficial effects in mice with clear additive effects of the combination treatment for body weight, fat mass, grip strength and steatosis, and non‐additive effects on hepatic inflammation and fibrosis. This suggests that most effects of exercise and dietary switch are mechanistically complementary and improve sarcopenic obesity, while anti‐inflammatory and anti‐fibrotic effect may involve similar pathways that do not add up.

**8-02**

**Exercise pre‐conditioning diminishes solute carrier protein expression and doxorubicin accumulation in the diaphragm**

**Ashley J. Smuder**, Andres Mor Huertas and Aaron B. Morton

*Department of Applied Physiology and Kinesiology, University of Florida, Gainesville, FL, USA*

**Introduction:** Doxorubicin (DOX) is an anthracycline antibiotic used in cancer treatment. Unfortunately, the clinical use of this highly efficacious anticancer drug is limited due to the development of respiratory and diaphragm muscle dysfunction in patients. DOX‐induced ventilatory impairment is a debilitating condition that promotes the onset of dyspnoea, fatigue,and exercise intolerance. While the mechanisms responsible for DOX‐induced respiratory insufficiency are unclear, previous work demonstrates that the incidence of ventilatory dysfunction greatly correlates to the concentration of DOX taken up by the diaphragm. In this regard, we recently demonstrated that endurance exercise performed prior to DOX administration is sufficient to reduce the accumulation of DOX within the diaphragm and prevent ventilatory dysfunction. While the mechanisms for the exercise‐induced reduction in diaphragm DOX levels are unknown, we hypothesize that endurance exercise alters the expression of solute carrier proteins (SLCs) required for the influx of DOX into the diaphragm.

**Methods:** To determine if exercise training can alter SLC protein expression in the diaphragm, animals underwent 2 weeks of treadmill running (5 days/week; 60 min/day; 30 m/min). Twenty‐four hours following the exercise training period, animals received either saline or DOX (20 mg/kg, i.p.) treatment.

**Results:** DOX administration resulted in a significant increase in diaphragm expression of SLC28A3, a transport protein associated with DOX import. Importantly, exercise training mitigated the DOX‐induced increase in SLC28A3 in the diaphragm. Exercise training also reduced the DOX‐induced increase in the expression of the mitochondria‐localized SLC transporters SLC25A1 and SLC25A37. Finally, the DOX‐induced increase in diaphragm SLC22A2 was attenuated in exercise trained animals treated with DOX.

**Conclusions:** These data demonstrate that DOX enhances the expression of several SLCs in the diaphragm and that exercise may ameliorate diaphragm DOX accumulation by preventing the up‐regulation of proteins with the potential to promote DOX cellular influx.

**8-03**

**Role of dynapaenia for risk of incident metabolic syndrome and diabetes mellitus in male adults**

**Wei‐Liang Chen** ^1,2^, Hui‐Fang Yang^1,2^, Tung‐Wei Kao^1,2^ and Chung‐Ching Wang^1,2^

^1^ *Division of Family Medicine, Department of Family and Community Medicine, Tri‐Service General Hospital; and School of Medicine, National Defense Medical Center, Taipei, Taiwan, Republic of China;* ^2^ *Division of Geriatric Medicine, Department of Family and Community Medicine, Tri‐Service General Hospital; and School of Medicine, National Defense Medical Center, Taipei, Taiwan, Republic of China*

**Introduction:** Emerging evidence elucidated that muscle mass was significantly correlated strength, and loss of strength was a direct aetiology of loss of muscle mass. However, little study focused on the effect of low muscle power (dynapaenia) on the adult population. The aim of our study was to investigate the effect of dynapaenia on future metabolic health in the young and middle‐aged men in Taiwan.

**Methods:** From the Healthy Examinations Registers and Fitness Registers of Taiwanese military service, our cohort study included a total of 20 670 eligible male participants who aged 20 years and older during the period 2013 to 2015. All enrolled participants underwent these three fitness tests, including 2 min push‐up, 2 min sit‐up, and 3000 m none weight‐bearing running exercise test. The performance of each fitness tests was classified into quartiles, and the lowest quartile group was categorized as dynapaenia. During 2 years follow‐up, longitudinal analyses were performed to examine whether the numbers of dynapaenia predicted the incident metabolic syndrome (MetS), hypertension (HTN), and diabetes mellitus (DM).

**Results:** In the multivariate regression model, the unadjusted odds ratio of the severe dynapaenia compared to the normal status was 1.745 (*P* < 0.001) in MetS, 5.935 (*P* < 0.001) in DM, and 1.171 (*P* = 0.133) in HTN. After further adjusting for pertinent variables, an inverse association between the degree of dynapaenia and metabolic health remained essentially unchanged in MetS and DM, but not in HTN.

**Conclusions:** Increased the severity of dynapaenia associated with the higher risks of MetS and DM in male adults, which was stratified based on three fitness tests. For the Risk stratification of adult population, fitness tests may be a useful assessment to examine the presence of low muscle strength or dynapaenia.

**8-04**

**Plasma IGF‐1 levels are decreased after body weight gain with increasing food intake together with additive exercise**

**Tetsuo Yamada** ^1^, Shin‐ichi Kurasawa^1^, Masami Matsuzaki^1^, Yoko Momose‐Sato^1^ and Akira Tanaka^2^

^1^ *Department of Nutrition and Dietetics, College of Nutrition, Kanto Gakuin University, Japan;* ^2^ *Laboratory of Clinical Nutrition and Medicine, Kagawa Nutrition University, Japan*

**Introduction:** Exercise and enhancing nutrient intakes are expected for improvement in muscle wasting, and insulin‐like growth factor‐1 (IGF‐1) is known to be involved in muscular hypertrophy. We investigated the effects of 5‐day of endurance exercise programme on protein metabolism including changes in plasma IGF‐1 levels under dietary conditions of positive energy balance.

**Methods:** Seven healthy male volunteers (age, 21 ± 1 years; body mass index, 21.7 ± 2.2 kg/m^2^; means ± SD) participated in a 5‐day body weight gain programme preceded by a 5‐day period which was set for stabilization of nutritional status. During the stabilization period, subjects were fed a diet based on estimated energy requirement, recommended dietary allowance, or adequate intake published in the Dietary Reference Intakes for Japanese. During body weight gain programme, energy intake was increased by 30% of the level during the stabilization period, and all foods were increased in proportion to the energy intake. In addition, the subjects exercised on a bicycle ergometer to consume an additional energy correspond to 20% of the level during the stabilization period. Thus, energy balance was positively set by 10% of the energy intake level during the stabilization period.

**Results:** Body weight significantly (*P* < 0.05) increased during body weight gain programme (before vs. after: 63.46 ± 7.85 vs. 63.78 ± 7.75 kg). Basal metabolic expenditure also significantly increased after the programme. Serum urea nitrogen (UN) levels tended to increase (*P* = 0.063), and the ratio of urinary UN excretion levels to energy intake levels was significantly lower in weight gain programme than in stabilization period. On the other hand, plasma IGF‐1 levels were significantly decreased, and testosterone levels were unchanged.

**Conclusions:** At the beginning of the exercise period, a decrease in fasting plasma IGF‐1 levels early in the morning is induced even under the dietary conditions of positive energy balance.

**8-05**

**Determinants of worsening in the chair‐stand‐test in older men—the prospective STRAMBO study**

**Pawel Szulc**, Dominique Foesser and Roland Chapurlat

*INSERM UMR 1033, Unviersity of Lyon, Hospices Civils de Lyon, Lyon, France*

**Introduction:** The five‐time chair‐stand‐test (CST) is a measure of lower body strength in the elderly. Our aim was to assess the risk factors of incident worsening in CST in older men.

**Methods:** Eight hundred and one men aged 60–87 and able to perform CST were followed up prospectively for 8 years. During this period, 641 men had at least one follow‐up test. The worsening in CST was defined by an increase in the time necessary to perform CST by >1 s/year or an incident incapacity to perform CST.

**Results:** The worsening was found in 147 men and increased with age (OR = 1.39 per 5 years, 95% CI: 1.18–1.63, *P* < 0.001). Compared with men having BMI 21–25 kg/m^2^, worsening was more frequent in 9 men with BMI < 21 kg/m^2^ (OR = 6.22, 95% CI: 1.36–28.47, *P* < 0.05) and in 43 men with BMI > 33 kg/m^2^ (OR = 2.59, 95% CI: 1.06–6.30, *P* < 0.05). Longer time to perform a 10‐step tandem walk (>16 s, upper quartile) was associated with higher risk of worsening (OR = 2.29 vs. three lower quartiles combined, 95% CI: 1.48–3.54, *P* < 0.001). The risk of worsening was higher in men self‐reporting fragility fracture of the lower limbs (OR = 3.77, 95% CI: 1.37–10.33, *P* = 0.01) and of upper limbs (OR = 3.33, 95% CI: 1.36–8.17, *P* < 0.01). It increased with the number of limb fractures (trend *P* < 0.001) being the highest in men with ≥2 fractures (OR = 16.08 vs. no fracture, 95% CI: 2.42–180.94, *P* < 0.05). The risk of worsening was higher in men with parathyroid hormone (PTH) level > 72 pg/mL (>mean + 3SD in young men) versus men with PTH < 36 pg/mL (<mean in young men): OR = 2.33, 95% CI: 1.13–4.79, *P* < 0.05. The worsening in CST was not related to the lifestyle, co‐morbidities, or testosterone deficit.

**Conclusions:** In older men, worsening in CST is predicted by higher age, extreme BMI, poor dynamic balance, prior fragility fracture of the limbs, and high PTH concentration.

**8-06**

**Three months of strength training changes the gene expression of inflammation‐related genes in PBMC of older women**

**Keliane Liberman** ^1^, Rose Njemini^1^, Louis Nuvagah Forti^1^, Wilfried Cools^2^, Florence Debacq‐Chainiaux^3^, Ron Kooijman^4^, Ingo Beyer^1,5^ and Ivan Bautmans^1,2^

^1^ *Frailty in Ageing research group (FRIA), Vrije Universiteit Brussel (VUB), Brussels, Belgium;* ^2^ *Interfaculty Center Data Processing and Statistics (ICDS), Vrije Universiteit Brussel (VUB), Brussels, Belgium;* ^3^ *URBC, NAmur Research Institute for Life Science (NARILIS), University of Namur, Namur, Belgium;* ^4^ *Center for Neurosciences (C4N), Vrije Universiteit Brussel, Brussels, Belgium;* ^5^ *Geriatrics Department, Universitair Ziekenhuis Brussel, Brussels, Belgium*

**Introduction:** Exercise can counter chronic low‐grade inflammatory profile (CLIP). The involvement of peripheral blood mononuclear cells (PBMC) remains unclear. We investigate changes of inflammation‐related gene‐expression in PBMC by strength training at different modalities.

**Methods:** Fourteen women aged ≥65 years were randomized into 3 months of either 3×/week intensive strength training (IST: 3 × 10 reps at 80% 1RM), strength endurance training (SET: 2 × 30 reps at 40% 1RM), or control (CON: 3 × 30 s stretching). RNA was extracted from isolated PBMC from blood samples taken before and after 3 months training. Targeted RNA sequencing including 407 inflammation‐related genes was performed, and differentially expressed genes were identified. Fold changes ≤0.5 or ≥1.5 were considered as significant. Pathway analysis was performed using IPA, *z*‐scores ≥2 or ≤ −2 were considered as significantly enriched.

**Results:** Eighty‐five genes, mostly pro‐inflammatory (*n* = 56), showed significant exercise‐induced changes in expression. IST and SET altered only 9 genes in similar direction (e.g. MXRA5 FC _IST_ = 23.54 and FC _SET_ = 6.78) whereas 26 genes were altered in opposite direction (e.g. IL1A FC _IST_ = 0.15 and FC _SET_ = 2.27). Compared to CON, IST induced changes in expression of 5 genes in the same direction, and for 15 genes in the SET group (e.g. ILTRAPL2 FC _IST_ = 20.52, FC _SET_ = 6.94 and FC _CON_ = 5.54). Likewise, 13 and 7 genes were oppositely expressed for respectively IST and SET compared to CON (e.g. MXRA5 FC _CON_ = 0.29). For IST and SET, pro‐inflammatory pathways were inhibited such as dendritic cell maturation pathway (IST *z*‐score = −2.94) and Sirtuin pathway (SET *z*‐score = −2.52). None of the enriched pathways overlapped between IST and SET. LXR/RXR and TREM1 pathways were enriched oppositely in both groups (respectively IST *z*‐score = 2.04 and *z*‐score = −2.04, SET *z*‐score = −2.04 and *z*‐score = 2.41).

**Conclusions:** Three months strength training at high and at moderate external load can both induce changes in CLIP‐related gene expression in PBMC, but by affecting different genes and related pathways.

**8-07**

**Tele‐rehabilitation to prevent sarcopenia and disability in advanced cancer: a randomized controlled trial**

**Andrea L. Cheville** ^1^ and Jeph Herrin^2^

^1^ *Department of Physical Medicine and Rehabilitation, Care Experience Program, Robert D. and Patricia E. Kern Center for the Science of Health Care Delivery, Mayo Clinic, Rochester, MN, USA;* ^2^ *Yale University Medical Center, New Haven, CT, USA*

**Introduction:** The majority of patients with advanced cancer develop progressive debility driven, in part, by sarcopenia. The trial was conducted to determine whether tele‐rehabilitation and pharmacological pain management improve physical function, fatigue, and utilization and whether preserved muscle endurance mediates these effects.

**Methods:** The study was a three‐arm randomized trial conducted at three academic medical centres within one health system. Participants (*N* = 516) were low‐level community or household ambulators with stage IIIC or IV solid or haematologic malignancies. Participants were randomly assigned to (i) control, (ii) tele‐rehabilitation, and (iii) tele‐rehabilitation + pharmacological pain management. Participants in Arms 2 and 3 received 6 months of centralized tele‐rehabilitation provided by a physical therapist (PT)‐physician team. Those in Arm 3 also received nurse‐coordinated pharmacological pain management. The main outcome measures included blinded assessments at baseline and months 3 and 6 for function [Activity Measure for Post Acute Care (AM‐PAC)] and fatigue [Brief Fatigue Inventory (BFI)]. Muscle endurance was assessed by repeated sit‐to‐stand at baseline and PT visits.

**Results:** Compared with the control group, the tele‐rehabilitation Arm 2 group had improved function (1.3; 95% confidence interval [CI] 0.08 to 2.35; P = 0.03). Tele‐rehabilitation was associated with higher odds of home discharge in Arms 2 (OR 4.3; 95% CI 1.3 to 14.3; P = 0.02) and 3 (OR 3.8; 95% CI 1.1 to 12.4; P = 0.03) and shorter hospitalizations in Arm 2 (difference ‐3.9 days; 95% CI ‐2.4 to ‐4.6; P = 0.01). Tele‐rehabilitation was not associated with changes in BFI scores. Change in sit‐to‐stand repetitions was significantly associated with improvement in AM‐PAC scores.

**Conclusions:** Tele‐rehabilitation improved physical function and reduced utilization among patients with stage IIIC or IV solid or haematologic malignancies. These effects were partly mediated by improved muscle endurance.

**8-08**

**Improved gait speed and clinical outcome in cardiovascular disease patients with slow gait speed**

**Kana Kawai** ^1^, Masakazu Saitoh^1^, Akihiro Sakuyama^1^, Reina Uewaki^1^, Junko Sakamoto^1^, Kazuki Kon^1^ and Masatoshi Nagayama^2^

^1^ *Department of Cardiac Rehabilitation;* ^2^ *Department of Cardiology, Sakakibara Heart Institute, Japan Research Promotion Society for Cardiovascular Disease, Japan*

**Background:** We examined the prognostic significance of increased in gait speed (GS) in cardiovascular disease patients with slow GS.

**Methods and Results:** A total of 202 cardiovascular patients (mean age: 74.8 ± 9.3 years, 46% of female) with slow GS at the beginning of outpatient cardiac rehabilitation (phaseIICR) programme between January 2015 and March 2018 were enrolled in this study. Slow GS and improved GS were defined as <1.0 m/s at the baseline, and improved GS was defined as increase in 0.1 m/s of GS through 3‐month phaseIICR programme. The primary outcome of this study was composed endpoint of all‐cause death on readmission. A total of 7 patients died, and 35 patients were readmitted over a mean follow‐up periods of 56 ± 28.9 months. Atotal of 138 patients (68%) improved GS through phaseIICR programme. Improved GS showed significant inverse association with composed endpoint of all‐cause death on readmission (hazard ratio: 0.390; 95% CI: 0.202–0.750; *P* = 0.005) after adjusted for age, gender, and left ventricular ejection fraction.

**Conclusions:** Improved GS through 3‐month phaseIICR programme was associated with reduced risk of composed endpoint of all‐cause death on readmission.

**8-09**

**Determinant of functional status in elderly heart failure patients: muscle function versus muscle mass**

**Suguru Honma** ^1^, Satoshi Katano^2^, Toshiyuki Yano^3^, Katsuhiko Ohori^3,4^, Takuya Inoue^2^, Yuhei Takamura^2^, Ryohei Nagaoka^2^, Akiyoshi Hashimoto^5,6^, Nobuhiro Yoshioka^6^ and Tetsuji Miura^3^

^1^ *Department of Rehabilitation, Sapporo Cardiovascular Hospital, Sapporo, Japan;* ^2^ *Division of Rehabilitation, Sapporo Medical University Hospital, Sapporo, Japan;* ^3^ *Department of Cardiovascular, Renal and Metabolic Medicine, Sapporo Medical University School of Medicine, Sapporo, Japan;* ^4^ *Department of Cardiology, Hokkaido Cardiovascular Hospital, Sapporo, Japan;* ^5^ *Division of Health Care Administration and Management, Sapporo Medical University School of Medicine, Sapporo, Japan;* ^6^ *Department of Cardiology, Sapporo Cardiovascular Hospital, Sapporo, Japan*

**Introduction:** The extent of contributions of muscle mass (MM) and muscle function (MF) on functional status in elderly heart failure patient has not been characterized.

**Methods:** We retrospectively analysed data for 208 patients [median age, 78 years (interquartile range, 72–83 years); females, 48%] who were admitted to our institute for diagnosis and treatment of heart failure. Each patient underwent a dual energy X‐ray absorptiometry (DEXA) scan to measure appendicular skeletal mass index (ASMI) as an indication of MM and a five‐times sit‐to‐stand test (FTSS) as an index of MF. In the FTSS, the time a patient took to consecutively stand up 5 times from a seated position on a chair was recorded. Low ASMI and low MF were defined as follows: low ASMI, <7.0 kg/m^2^ in men and < 5.4 kg/m^2^ in women; low MF, ≥11 s in men and ≥ 12 s in women. Functional dependence (FD) was defined as a Barthel Index score of 85 points or lower.

**Results:** Fifty‐four patients (26%) were diagnosed as having functional dependence. ASMI was significantly lower (5.25 vs. 5.90 kg/m^2^, *P* < 0.01), and FTSS time was significantly longer (15.0 vs. 10.6 s, *P* < 0.01) in patients with (FD) than in those without FD. In multivariate logistic regression analysis with adjustments for potential confounders, low MM, and low MF were independent predictors of FD. An increase in adjusted odds ratio for predicting the presence of FD was observed across subgroups in the following order: patients with low MM (5.42), those with low MF (9.48), and those with low MM and low MF (18.58). There was no significant association between ASMI and FTSS time (*r* = −0.11, *P* = 0.11).

**Conclusions:** Reduction in MM and decline in MF independently and incrementally predict the presence of FD in elderly heart failure patients.

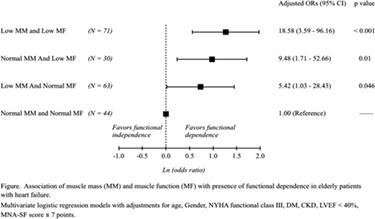

**8-10**

**Anaerobic threshold‐based exercise training programme stimulates genes that control skeletal muscle differentiation and function in heart failure patients**

**Renata I. Dmitrieva** ^1^, Oksana A. Ivanova^1,2^, Tatiana A. Lelyavina^1^, Victoria L. Galenko^1^, Elena V. Ignatieva^1^, Margarita Y. Komarova^3^, Natalia V. Khromova^1^, Maria Y. Sitnikova^1^, Alexey Sergushichev^2^ and Anna A. Kostareva^1^

^1^ *National Almazov Medical Research Centre, Saint‐Petersburg, Russia;* ^2^ *ITMO University, St. Petersburg, Russia;* ^3^ *Peter the Great St. Petersburg Polytechnic University, Saint‐Petersburg, Russia*

**Background:** Heart failure is associated with skeletal muscle wasting and limited ability to exercise leading to worsening of heart failure‐induced metabolic disorders. The aim of this study was to evaluate the signalling response to personalized exercise therapy in heart failure patients in order to determine the molecular mechanisms behind therapeutic effects and to provide the background for the search of new therapeutic targets for treatment of muscle wasting.

**Methods:** 234 patients (176 male, 61 female) with HF NYHA III class were enrolled. The individual training programme was determined at 90% of anaerobic threshold; all patients underwent cardiopulmonary exercise testing (VO2 peak), echocardiography (LVEF), quality of life, and exercise tolerance assessment before and after 24 weeks of training. The muscle biopsies were taken from subgroup of eight patients before and after of 12 weeks of training: histology analysis was used to evaluate changes in muscle morphology; RNA‐Seq analysis was employed to uncover gene expression changes that may contribute to beneficial effects of exercise training programmes in HF patients.

**Results:** 95% of patients responded positively to physical rehabilitation programme and 30% of those showed the improvement of all indicators used in the study: LVEF, VO2 peak, exercise tolerance, and quality of life. RNA‐Seq analysis revealed training‐induced coordinated up‐regulation of expression of signalling pathways and genes that involved in control of skeletal muscle differentiation and function including: significant up‐regulation of RYR1, a calcium release channel; FLNC, the muscle‐specific filamin critical for maintaining the structural integrity of the muscle fibers; CAVIN4, that facilitate myofibrillar organization; GSN, calcium‐regulated, actin‐modulating protein that promote the assembly of monomers into filaments; NACA, involved in regulation of post‐natal skeletal muscle growth and regeneration, as well as PLEC, involved in regulation of filaments network; CYR61 that promotes angiogenesis and regeneration.

**Conclusions:** Our data reveal the specific genes that may be considered as the potential contributors to the therapeutic response to personalized exercise therapy in heart failure patients, as well as prognostic markers and therapeutic targets.

**Acknowledgement:** This work was funded by Russian Science Foundation grant #16‐15‐10178‐П.

**9-01**

**Effects of exercise and β‐hydroxy‐β‐methylbutyrate supplements on muscle mass, muscle strength, and physical performance in older women with muscle atrophy: a randomized, double‐blind, placebo‐controlled trial**

**Yosuke Osuka** ^1^, Narumi Kojima^1^, Kyohsuke Wakaba^2^, Daiji Miyauchi^3^, Kiyoji Tanaka^2^ and Hunkyung Kim^1^

^1^ *Research Team for Promoting Independence and Mental Health, Tokyo Metropolitan Institute of Gerontology, Japan;* ^2^ *Faculty of Health and Sport Sciences, University of Tsukuba, Japan;* ^3^ *Kyowa Co., Ltd., Japan*

**Introduction:** The effects of exercise and β‐hydroxy‐β‐methylbutyrate (HMB) on muscle outcomes and physical performance and their residual effects are unclear among individuals with muscle atrophy. This randomized, double‐blind, placebo‐controlled trial aimed to examine the chronic and residual effects of exercise and/or HMB supplementation on muscle mass, muscle strength, and physical performance among older women with muscle atrophy.

**Methods:** A total of 156 older women with skeletal muscle mass index <5.7 kg/m^2^ were randomly allocated to undergo one of the four interventions (exercise + HMB, exercise + placebo, education + HMB, and education + placebo) for 12 weeks and were followed up for further 12 weeks to observe their residual effects. The participants were provided exercise programmes twice weekly or education programmes every 2 weeks and HMB (1200 mg) or placebo supplements once daily. The primary outcome was change in muscle mass. The secondary outcomes included change in muscle strength, physical performance, blood parameters, and functional capacity.

**Results:** Among the 156 participants, 149 and 144 were followed up at weeks 12 and 24, respectively, and included in the intention‐to‐treat analyses. Analyses of variance showed no significant exercise × HMB interactions on any of the outcomes, but significant effects of exercise were seen on knee extensor and hip adductor strength, usual and maximal gait speed, timed up‐and‐go, sit‐to‐stand, functional capacity, and HbA1c, while the effects of HMB on usual gait speed and triglyceride levels were observed. However, the significant effects on such outcomes except for timed up‐and‐go and sit‐to‐stand disappeared after the next 12 weeks.

**Conclusions:** Exercise plus HMB supplementation would provide more favourable effects on muscle strength, mobility, glucose and lipid metabolism, and general functional condition. However, most of these effects were lost after the next 12 weeks of observation, indicating the importance of continuing the exercise and HMB supplementation programme.

**9-02**

**Oral DHA supplementation modulates serum epoxydocosapentaenoic acid levels in a cohort of women with breast cancer**

**Alessio Molfino** ^1^, Maria Ida Amabile^2^, Cesarina Ramaccini^1^, Luana Lionetto^3^, Alessandra Spagnoli^4^, Maurizio Simmaco^3^, Alessandro Laviano^1^, Massimo Monti^2^ and Maurizio Muscaritoli^1^

^1^ *Department of Translational and Precision Medicine, Sapienza University of Rome, Rome, Italy;* ^2^ *Department of Surgical Sciences, Sapienza University of Rome, Rome, Italy;* ^3^ *Department NESMOS, Sapienza University of Rome, Analytical Laboratory Unit, Sant'Andrea Hospital, Rome, Italy;* ^4^ *Department of Public Health and Infectious Diseases, Sapienza University of Rome, Rome, Italy*

**Introduction:** The omega‐3 polyunsaturated fatty acids, as docosahexaenoic acid (DHA), are considered mediators for resolution of inflammation during cancer and possibly associated with better outcomes. Metabolites of the DHA, as the epoxydocosapentaenoic acids (EDPs), are hypothesized to be responsible for some of DHA beneficial effects. We aimed to assess the circulating 19,20‐EDP levels in breast cancer (BC) patients and in healthy controls before and after oral DHA supplementation and the potential differences in the DHA conversion in 19,20‐EDPs between patients with different BC presentations.

**Methods:** BC patients and healthy controls were supplemented with DHA in form of algal oil for 10 days (2 g/day). Blood samples were collected at baseline (T0) and after supplementation (T1) to assess 19,20‐EDP serum levels by liquid chromatography spectrometry.

**Results:** 33 BC patients and 10 healthy controls were studied. EDP values at T0 were not different between patients and controls. At T1, we found an increase in 19,20‐EDP levels in BC patients (*P* < 0.00001) and in controls (*P* < 0.001), whereas no differences in 19,20‐EDPs were present between the two groups; when considering the type of BC presentation, patients with BRCA1/2 mutation showed lower 19,20‐EDPs levels with respect to BC patients without the mutation (*P* = 0.03). According to immunohistochemical subtype, luminal A‐like BC patients showed at T1 higher 19,20‐EDP levels compared to nonluminal A (*P* = 0.02).

**Conclusions:** Oral DHA supplementation was associated with increased 19,20‐EDP serum levels in BC patients, independent of the type of BC presentation, and in controls. Patients carrier of BRCA1/2 mutation seemed to possess lower ability of DHA epoxidation, whereas luminal A‐like BC patients showed higher EDP conversion. This pattern should be further investigated in a larger population.

**9-03**

**Leucine supplementation does not decrease cancer‐induced sarcopenia in cancer older patients**

**Jéssika D.P. Soares** ^1^, Jéssika M. Siqueira^1^, Izabella C.L. Oliveira^1^, Alessandro Laviano^2^ and Gustavo D. Pimentel^1^

^1^ *Laboratory of Research in Clinical Nutrition and Sports (Labince), Faculty of Nutrition, Federal University of Goiás, Goiânia, Brazil;* ^2^ *Department of Translational and Precision Medicine, Sapienza University, Rome, Italy*

**Introduction:** Cancer patients undergoing chemotherapy show cancer‐induced weight loss and sarcopenia. However, whether l‐leucine supplementation alleviates the cancer‐mediated wasting is not clear. In this study, we sought to evaluate the effects of leucine supplementation in cancer‐induced sarcopenia in older patients.

**Methods:** An 8 week randomized, double‐blind clinical trial enrolling 35 male GI cancer patients ≥60 years receiving chemotherapy was completed. The patients were divided into two groups: l‐leucine (LG: 7.2 g/day leucine, *n* = 17) and control (CG: 7.2 g/day collagen, *n* = 18). Body weight (kg), height (m), and BMI (kg/m^2^) were assessed before and at the end of the experimental period. Skeletal muscle mass (SMM) was assessed by bioelectrical impedance (BIA) and SMM index was calculated using the formula SMM/height (SMI; kg/m^2^): handgrip strength (HGS) and functional capacity were measured by dynamometer (kg) and by gait speed (m/s), respectively. Sarcopenia according to the criteria set by the European Working Group on Sarcopenia in Older People (EWGSOP2). Results were statistically analysed using Student's *t*‐test and Fisher's exact tests. Data are presented as M ± SD; statistical significance was set at *P* < 0.05.

**Results:** Body weight (ΔLG = +2.67% vs. ΔCG = +0.85%; *P* = 0.003) and BMI (ΔLG = 2.62% vs. ΔCG = 1.98%; *P* = 0.01) increased in LG patients when compared to CG patients. No differences were found for SMM (ΔLG = +4.69% vs. ΔCG = +1.79%; *P* = 0.17), SMI (ΔLG = +4.69% vs. ΔCG = +1.79%; *P* = 0.16), HS (ΔLG = +2.76% vs. ΔCG = +3.55%; *P* = 0.22) and gait speed (ΔLG = −2.54% vs. ΔCG = 3.19%; *P* = 0.27). Also, the degree of sarcopenia did not improve significantly in LG versus CG patients (pre‐intervention versus post‐intervention).

**Conclusions:** Eight weeks of l‐leucine supplementation promotes higher gain body weight and BMI than collagen. However, it was not able to decrease the cancer‐induced sarcopenia in older cancer patients undergoing chemotherapy.

**9-04**

**High‐protein diet, not BCAA, is associated with skeletal muscle mass in gastrointestinal cancer patients**

Jéssika D.P. Soares^1^, Jéssika M. Siqueira^1^, Izabella C.L. Oliveira^1^ and Alessandro Laviano and **Gustavo D. Pimentel** ^1,2^

^1^ *Laboratory of Research in Clinical Nutrition and Sports (Labince), Faculty of Nutrition, Federal University of Goiás, Goiânia, Brazil;* ^2^ *Department of Clinical Medicine, Sapienza University, Rome, Italy*

**Introduction:** Cancer patients are susceptible to lose skeletal muscle mass. Thus, the purpose of this study was to evaluate whether a high‐protein diet (HPD) or isolated BCAA intake is associated with skeletal muscle mass index (SMI) in gastrointestinal tract cancer patients.

**Methods:** Cross‐sectional observational study included 106 gastrointestinal tract tumours patients. Food consumption was estimated using the 24 h food recall. Patients were divided into two groups: low‐protein diet (LPD): ≤1.2 g/kg/day and high‐protein diet (HPD): >1.2 g/kg/day. The appendicular muscle mass (ASM) was calculated using Lee's formula, and its values were divided by the square of the height to obtain the SMI. Any significant differences was set at 5% (*P* < 0.05).

**Results:** Out of 106 patients assessed, 69 (65%) had LPD ingestion (LPD ≤ 1.2 g/kg/day) and 37 (35%) had HPD intake (>1.2 g/kg/day). Logistic regression after adjusting by sex, caloric, and carbohydrate consumption showed association between SMI and HPD [OR: 4.19, 95% CI (1.06–16.56), *P* < 0.001], but not with branched chain amino acids (BCAA). Daily total protein intake, but not isolated BCAA or leucine, was able to predict an increase in SMI in 43% of patients (*P* = 0.0069).

**Conclusions:** HPD was associated with SMI, and total protein intake was a better predictor of SMI than BCAAs. Thus, HPD seems to be more cost‐effective in clinical practice in predicting SMI than the recurrent concern with the ingestion of isolated BCAAs.

**9-05**

**Results of a randomized controlled trial evaluating safety and feasibility of a low to moderate intensity exercise regimen in patients with GI cancers and cachexia**

**Richard F. Dunne** ^1^, Aminah Jatoi^2^, Supriya G. Mohile^1^, Nicholas Gerbino^1^, Javier Bautista^1^, Michelle C. Janelsins^1^, Marcus S. Noel^1^, Erika Ramsdale^1^, Aram F. Hezel^1^ and Karen M. Mustian^1^

^1^ *University of Rochester‐Wilmot Cancer Institute, Rochester, NY, USA;* ^2^ *Mayo Clinic, Rochester, MN, USA*

**Introduction:** Strong scientific rationale supports the use of exercise as an intervention for cancer cachexia, yet few randomized trials (RCTs) exist. We conducted an RCT evaluating the safety and feasibility of a home‐based exercise intervention in patients with pre‐cachexia and cachexia.

**Methods:** University of Rochester patients >18 years with incurable gastrointestinal cancers with pre‐cachexia or cachexia starting first‐line chemotherapy were eligible. Pre‐cachexia was defined as 2–5% weight loss over the prior 6 months with hypoalbuminemia, anaemia, or impaired glucose tolerance and cachexia was defined as >5% weight loss. Subjects were randomized to usual care (UC) or EXCAP, a 12‐week, structured, individually tailored home exercise programme consisting of a progressive walking programme and resistance‐band exercises. Pre‐changes/post‐changes in steps measured by activity tracker, fat free mass (FFM) by bioelectrical impedance, and 6‐min walk test distance (6MWT) were analysed by two‐sided Wilcoxon.

**Results:** Nineteen patients (12 male) with an average age of 67 years were randomized; 17 met criteria for cachexia (two pre‐cachexia). No deaths and no exercise‐related adverse events were reported on study. Exercisers engaged in 2.8 resistance sessions per week. Pre‐change/post‐change in average steps per day was significantly improved in exercisers compared to UC (*P* = 0.003, exercisers +1344 steps/day vs. UC −1153, difference of 2497 steps/day or approximately 1 mile). No significant difference in FFM was demonstrated (*P* = 0.709, mean change score in exercisers +0.82 lbs, +1.27 in UC). Pre‐6MWT/post‐6MWT was significantly improved in exercisers compared to UC (*P* = 0.019, Exercisers +156.2 ft vs. −101.25 ft in UC).

**Conclusions:** Our RCT is among the first to demonstrate the safety and feasibility of a tailored exercise programme in strictly pre‐cachectic/cachectic gastrointestinal cancer patients. Although exercise did not significantly improve lean mass, it significantly improved physical performance in an ill population. Cachexia trials investigating multi‐modality interventions including exercise are ongoing at our institution.

**9-06**

**Derangements of amino acids in cachectic skeletal muscle are caused by mitochondrial dysfunction**

**Thomas Kunzke** ^1^, Achim Buck^1^, Verena M. Prade^1^, Annette Feuchtinger^1^, Olga Prokopchuk^2^, Marc E. Martignoni^2^, Simone Heisz^3,4^, Hans Hauner^3,4^, Klaus‐Peter Janssen^2^, Axel Walch^1^ and Michaela Aichler^1^

^1^ *Research Unit Analytical Pathology, Helmholtz Zentrum München, Oberschleißheim, Germany;* ^2^ *Department of Surgery, Klinikum Rechts der Isar, TUM, Munich, Germany;* ^3^ *Else Kroener‐Fresenius‐Center for Nutritional Medicine, Klinikum Rechts der Isar, TUM, Munich, Germany;* ^4^ *ZIEL‐Institute for Food and Health, Nutritional Medicine Unit, TUM, Freising, Germany*

**Introduction:** Muscle proteins are massively degraded in cachexia; nevertheless, the molecular mechanisms related to this process are poorly understood. Previous studies have reported conflicting results regarding the amino acid abundances in cachectic skeletal muscle tissues. There is a clear need to identify the molecular processes of muscle metabolism in the context of cachexia, especially how different types of molecules are involved in the muscle‐wasting process.

**Methods:** New *in situ* omics techniques were used to produce a more comprehensive picture of amino acid metabolism in cachectic muscles by determining the quantities of amino acids, proteins, and cellular metabolites. Using MALDI mass spectrometry imaging, we determined the *in situ* concentrations of amino acids and proteins, as well as energy and other cellular metabolites, in skeletal muscle tissues from genetic mouse cancer models and from patients with cancer. Immunohistochemistry staining for mitochondrial proteins and myosin heavy chain expressions, digital image analysis, and transmission electron microscopy complemented the MALDI mass spectrometry imaging results.

**Results:** Metabolic derangements in cachectic mouse muscle tissues were detected, with significantly increased quantities of lysine, arginine, proline, and tyrosine and significantly reduced quantities of glutamate and aspartate. A majority of altered amino acids was released by the breakdown of proteins involved in oxidative phosphorylation. Decreased energy charge was observed in cachectic muscle tissues, which was related to the breakdown of specific proteins. Additionally, expression of the cationic amino acid transporter CAT1 was significantly decreased in the mitochondria of cachectic mouse muscles; this decrease may play an important role in the alterations of cationic amino acid metabolism and decreased quantity of glutamate observed in cachexia.

**Conclusions:** Our results suggest that mitochondrial dysfunction has a substantial influence on amino acid metabolism in cachectic skeletal muscles, which appears to be triggered by diminished CAT1 expression, as well as the degradation of mitochondrial proteins. These findings provide new insights into the pathobiochemistry of muscle.

**9-07**

**Serum amyloid A1 mediates myotube atrophy via toll‐like receptors**

Alexander Hahn^1^, **Melanie Kny** ^1^, Cristina Pablo‐Tortola^1^, Mihail Todiras^2,3^, Michael Willenbrock^4^, Sibylle Schmidt^1^, Katrin Schmoeckel^5^, Ilka Jorde^5^, Marcel Nowak^1,6^, Ernst Jarosch^6^, Thomas Sommer^6,7,8^, Barbara M. Bröker^5^, Stephan B. Felix^9,10^, Claus Scheidereit^4^, Steffen Weber‐Carstens^11,12^, Christian Butter^13^, Friedrich C. Luft^1^ and Jens Fielitz^1,9,10,12^

^1^ *Experimental and Clinical Research Center, Charité‐Universitätsmedizin Berlin, Max Delbrück Center for Molecular Medicine in the Helmholtz Association, Berlin, Germany;* ^2^ *Cardiovascular Hormones, Max Delbrück Center for Molecular Medicine in the Helmholtz Association, Berlin, Germany;* ^3^ *Nicolae Testemițanu State University of Medicine and Pharmacy, Chișinău, Moldova;* ^4^ *Signal Transduction in Tumor Cells, Max Delbrück Center for Molecular Medicine in the Helmholtz Association, Berlin, Germany;* ^5^ *Department of Immunology, Institute of Immunology and Transfusion Medicine, University Medicine, Greifswald, Germany;* ^6^ *Intracellular Proteolysis, Max Delbrück Center for Molecular Medicine in the Helmholtz Association, Berlin, Germany;* ^7^ *Institute of Biology, Humboldt‐University Berlin, Berlin, Germany;* ^8^ *DZHK (German Center for Cardiovascular Research), partner site Berlin, Berlin, Germany;* ^9^ *Department of Internal Medicine B, Cardiology, University Medicine Greifswald, Germany;* ^10^ *DZHK (German Center for Cardiovascular Research), partner site Greifswald, Greifswald, Germany;* ^11^ *Department of Anesthesiology and Intensive Care Medicine, Campus Virchow‐Klinikum and Campus Charité Mitte, Charité‐Universitätsmedizin Berlin, Berlin, Germany;* ^12^ *Berlin Institute of Health (BIH), Berlin, Germany;* ^13^ *Department of Cardiology, Heart Center Brandenburg and Medical University Brandenburg (MHB), Bernau, Germany*

**Background:** Critically ill patients frequently develop muscle atrophy and weakness in the intensive care unit (ICU acquired weakness, ICUAW). Sepsis, systemic inflammation, and acute‐phase response are the major risk factors for ICUAW. We reported earlier that the acute‐phase protein serum amyloid A1 (SAA1) is increased and accumulates in muscles of ICUAW patients, but its relevance was unknown. Our objectives were to investigate the role of SAA1 in inflammation‐induced muscle atrophy and to identify SAA1 receptors and their downstream signalling pathways in myocyte cultures and skeletal muscle.

**Methods:** We performed cell‐based *in vitro* and animal‐based *in vivo* experiments. The atrophic effect of SAA1 on differentiated C2C12 murine myotubes was investigated by analysing gene expression, protein content, and the atrophy phenotype. We used the cecal ligation and puncture (CLP) model to induce polymicrobial sepsis in wild type mice, which were treated with the IкB kinase inhibitor (BMS‐345541) or vehicle.

**Results:** Treatment of differentiated C2C12 myotubes with recombinant SAA1 caused myotube atrophy and increased interleukin 6 (IL‐6) gene expression. These effects were mediated by toll‐like receptor‐2 (TLR2) and TLR4. SAA1 increased the activity of the transcription factor NF‐κB p65 via TLR2 and TLR4 leading to an elevation of NF‐κB‐dependent gene expression. In polymicrobial sepsis of mice, skeletal muscle mass, tissue morphology, gene, and protein expression were associated with the atrophy response. Inhibiting NF‐κB signalling by BMS increased survival, reversed the inflammation‐induced atrophy programme and diminished skeletal muscle atrophy of septic mice.

**Conclusions:** SAA1 activates the TLR2/TLR4//NF‐κB p65 signalling pathway to cause myocyte atrophy. Inhibition of this atrophy pathway could have utility in combatting ICUAW.

**9-08**

**Iodide reduces intramuscular inflammation following hindlimb ischaemia in mice**

**Michael A. Insko** ^1^ and Mark B. Roth^2^

^1^ *Faraday Pharmaceuticals, Seattle, WA, USA;* ^2^ *Division of Basic Sciences, Fred Hutchinson Cancer Research Center, Seattle, WA, USA*

**Introduction:** Faraday Pharmaceuticals is focused on the research and development of elemental reducing agents (ERAs). These agents have potential applications for the treatment of critical care diseases. Inflammation and damage induced by reactive oxygen species (ROS) are well known to impair muscle leading to wasting. In this study, we investigated the utility of FDY‐5301 (sodium iodide) to reduce intramuscular inflammation in the mouse hindlimb following ischaemia.

**Methods:** Male C57BL/6 mice were anaesthetized and subject to 2.5 h of bilateral hindlimb ischaemia using an O‐ring placed above the thigh. Five minutes prior to O‐ring removal, a 1 mg/kg intravenous bolus of FDY‐5301 was given retro‐orbitally. The next day (24 h post‐O‐ring removal), the gastrocnemius muscle was removed and snap frozen in liquid nitrogen. Following homogenization, the levels of various cytokines were assessed using a MILLIPLEX® MAP magnetic bead‐based analysis on a Luminex MAGPIX® system. The following biomarkers were measured: interferon‐γ (IFN‐γ), interleukin 1β (IL‐1β), IL‐2, IL‐6, IL‐10, KC (a.k.a. CXCL1), lipopolysaccharide‐induced CXC chemokine (LIX), macrophage inflammatory protein‐2 (MIP‐2, a.k.a. CXCL2), and tumour necrosis factor alpha (TNF‐α).

**Results:** Administration of FDY‐5301 lead to a statistically significant reduction in muscle levels of: IL‐6, IL‐10, KC, and MIP‐2, and also reduced LIX and TNF‐α. A significant reduction in systemic IL‐6 in the plasma was also observed.

**Conclusions:** Iodide administration reduces intramuscular inflammation following bilateral hindlimb ischaemia.

